# Divergent organ-specific isogenic metastatic cell lines identified using multi-omics exhibit differential drug sensitivity

**DOI:** 10.1371/journal.pone.0242384

**Published:** 2020-11-16

**Authors:** Paul T. Winnard, Farhad Vesuna, Sankar Muthukumar, Venu Raman

**Affiliations:** 1 Division of Cancer Imaging Research, The Russell H Morgan Department of Radiology and Radiological Sciences, The Johns Hopkins University School of Medicine, Baltimore, Maryland, United States of America; 2 Department of Oncology, The Johns Hopkins University, School of Medicine, Baltimore, Maryland, United States of America; 3 Department of Pathology, University Medical Center Utrecht, Utrecht, The Netherlands; University of Nebraska Medical Center, UNITED STATES

## Abstract

**Background:**

Monitoring and treating metastatic progression remains a formidable task due, in part, to an inability to monitor specific differential molecular adaptations that allow the cancer to thrive within different tissue types. Hence, to develop optimal treatment strategies for metastatic disease, an important consideration is the divergence of the metastatic cancer growing in visceral organs from the primary tumor. We had previously reported the establishment of isogenic human metastatic breast cancer cell lines that are representative of the common metastatic sites observed in breast cancer patients.

**Methods:**

Here we have used proteomic, RNAseq, and metabolomic analyses of these isogenic cell lines to systematically identify differences and commonalities in pathway networks and examine the effect on the sensitivity to breast cancer therapeutic agents.

**Results:**

Proteomic analyses indicated that dissemination of cells from the primary tumor sites to visceral organs resulted in cell lines that adapted to growth at each new site by, in part, acquiring protein pathways characteristic of the organ of growth. RNAseq and metabolomics analyses further confirmed the divergences, which resulted in differential efficacies to commonly used FDA approved chemotherapeutic drugs. This model system has provided data that indicates that organ-specific growth of malignant lesions is a selective adaptation and growth process.

**Conclusions:**

The insights provided by these analyses indicate that the rationale of targeted treatment of metastatic disease may benefit from a consideration that the biology of metastases has diverged from the primary tumor biology and using primary tumor traits as the basis for treatment may not be ideal to design treatment strategies.

## Introduction

Breast cancer is the most common malignant neoplasm among women in the United States. Recently, the American Cancer Society reported a 5-year survival rate near 86–99% for regional and local breast cancer, respectively [1]. On the other hand, the 5-year survival for metastatic breast cancer that involves distant organs is only 27% [1]. This latter low survival rate is likely due in part to a difference in clonal divergence of the metastatic tumor growth as well as to the use of primary tumor characteristics as the rationale for treatment strategies of metastatic disease (e.g., [2]). Thus, there is a growing consensus that matched primary breast tumor and metastatic lesion biopsy samples often exhibit divergent expression of markers, for example, hormone receptors (HR: estrogen (ER) & progesterone (PR)) as well as HER2, which influences outcomes [3–8]. In addition, genomic sequencing studies are providing strong corroborating evidence that metastatic progression represents evolutionary processes that results in distinct biological entities at metastatic sites that have greatly diverged from the primary tumor [9–11]. Consequently, the current practice of primary tumor-based selection of chemotherapy is limited with respect to patient specific precision therapeutic targeting of a patient’s metastatic lesions as well as general therapeutic resistance.

Notably, monitoring and treating metastatic progression remains a formidable task due to many gaps in our knowledge including an inability to monitor specific differential molecular adaptations that allow the cancer to survive and thrive within different tissue types. This is a consequence of the fact that visceral organs differ vastly from one another with unique attributes of metabolism, developmental programs, microenvironments, and function resulting in defined physiological identities. Hence, in order to develop optimal treatment strategies for metastatic disease, an important consideration is the divergence of the metastatic cancer growing in visceral organs from the primary tumor [2, 4, 5, 11]. Accordingly, in order to gain insight into new treatment regimens aimed at controlling and ablating metastatic progression, there is an urgent need to evaluate the distinct molecular differences that exist between isogenic tumor cells growing at different metastatic sites and their sensitivity to different chemotherapeutic agents. To address this issue, as previously reported [12, 13], we established isogenic (analogous to patient metastatic cells) human metastatic breast cancer cell lines and have now included two additional cell lines starting from a well-established aggressive breast cancer cell line (MDA-MB-231). In all cases, these metastases spontaneously arose through dissemination from the primary mammary fat pad tumor site in a mouse model system. The resulting six metastatic cell lines are representative of the common metastatic sites of lymph node, lung, bone, liver, and brain observed in breast cancer patients [14]. This model system has provided excellent data that supports our principal hypothesis that organ-specific growth of malignant lesions is a selection process that results in cancer cells that have adapted distinct biochemical and molecular attributes that allow them to thrive at each site outside of the primary tumor site [12, 13].

Dissecting metastatic cancers based on objective molecular markers and metabolic pathways remains an outstanding challenge. Standard histochemical techniques are limited to identification of relatively few markers in, most frequently, primary tumor sections that may no longer be present at metastases [4–8]. Consequently, a goal of the presented study was to obtain proteomic and RNAseq data sets for our isogenic metastatic cell lines and analyze the resulting proteomes and transcriptomes with pathway analysis tools. Analyses of both data sets revealed that the metastatic cell lines had diverged from the primary tumor, consistent with our previous studies [12, 13]. We found, relative to the primary tumor, overlapping general common metastatic associated pathways, but importantly unique organ-specific pathways have also been uncovered. We hypothesize that the latter reflect processes of adaptation, i.e., the gaining of site (organ) specific attributes that reflects local microenvironmental influences resulting in selected gene expression and protein pathway preferences for each organ. Similar, to previous studies of comparisons of proteomic and RNAseq data [15–18], we discovered discordance between proteomes and transcriptomes as well as some common characteristic pathways. Consistent with the RNAseq data, RT-PCR, in general, only provided confirmation of very few of the expressed proteins discovered during proteomic screening. In addition, pathway analyses of metabolomes provided confirmations corresponding to associations to the proteomic-based and transcriptomic-based pathways. Finally, *in vitro* drug efficacy assays showed significant differential responses of ten cell lines, i.e., two parental cell lines, two primary tumor cell lines, and six metastatic cell lines, to the drugs that were tested. This latter data has provided evidence that chemotherapeutic regimes based on primary tumor markers may result in ineffective control of metastatic tumors due to the changes that have occurred within the tumor cells at those metastatic sites that affect a drug’s killing ability at the site.

## Results

### Phenotypic characterization of isogenic cell lines

**Fig 1:**
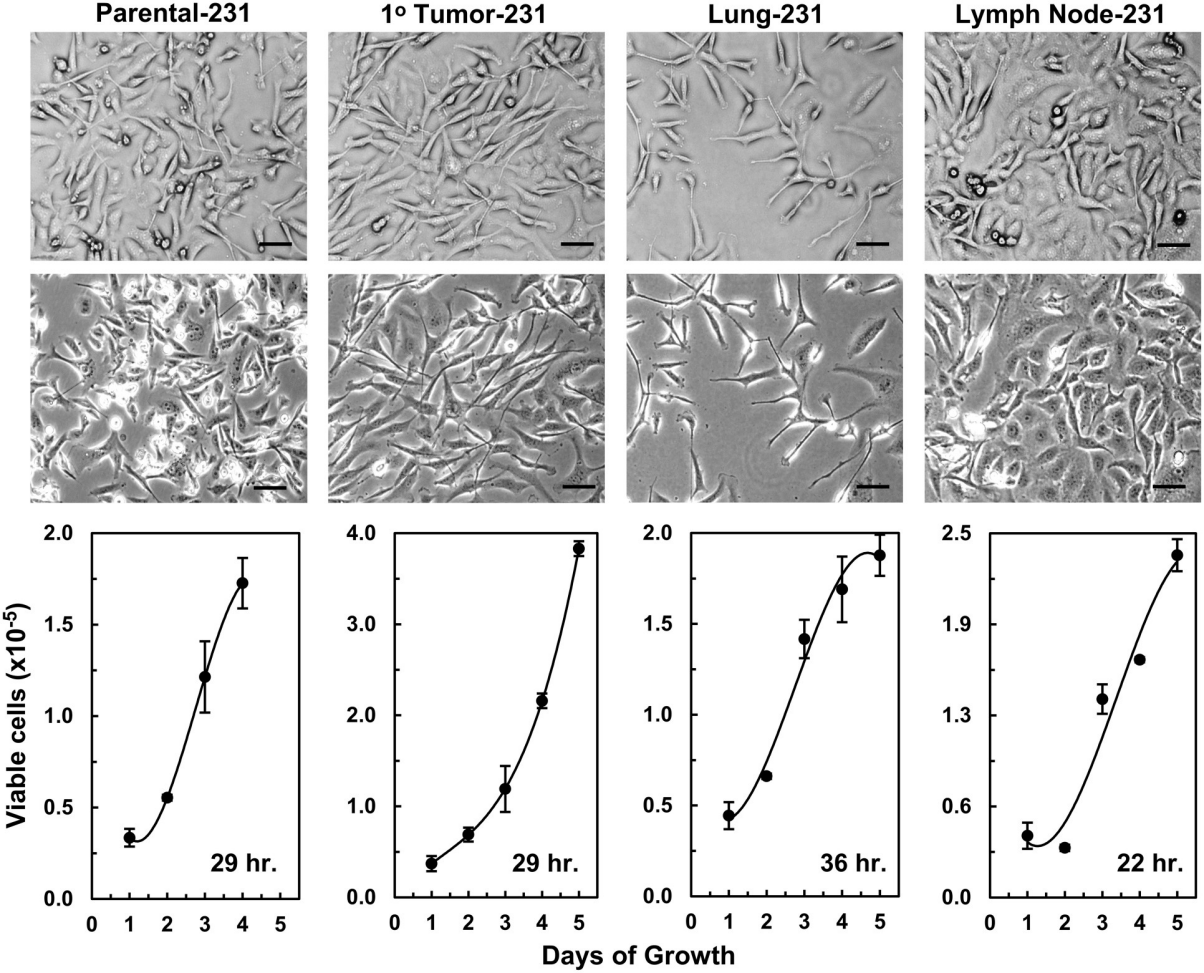
Phenotypic characterization of MDA-MB-231 isogenic cell lines. Phase-contrast images of the parental-231, primary tumor (1° tumor)-231, lung-231, and lymph node-231 cell lines are shown in the top two rows. The top row images were photographed using a X10 objective coupled with a X4 phase-contrast ring while the second-row images were duplicate images obtained with a X10 objective coupled with a X10 phase-contrast ring. The optical configuration of the top row gave 3D images. The black scale bars = 50 μM. Growth curves of each cell line are presented below the images of the corresponding cell line with the mean growth-rate given in the bottom right-hand corner of the curves.

As we previously reported for the isogenic cell lines generated from the MDA-MB-435 cells: parental-435 and its primary-tumor (1°-tumor), brain, liver, lung, and spine isogenic cell lines (S1 Fig) [12], the parental MDA-MB-231 (parental-231) and its isogenic cell lines (1°-tumor, lung, and lymph node) exhibited somewhat different morphologies when grown on plastic (All bright-field images: Fig 1 and S2 Fig). As has been reported [19], parental-231 cultures had a mixture of several cell morphologies that differed in shape and size and that grew in a chaotic overlapping manner (Far-left bright-field images: Fig 1 and S2 Fig). 1°-tumor-231 cells grew in a similar manner but appeared to have fewer cell morphologies with the majority of the cells in these cultures being relatively large, broad, and elongated with some extended spindle characteristics and few or no small cells (Middle-left bright-field images: Fig 1 and S2 Fig). In contrast, lung-231 cell cultures had even fewer small cells and the majority were medium sized spindly cells with some broadly larger elongated cells (Middle-right bright-field images: Fig 1 and S2 Fig). On the other hand, the majority of lymph node-231 cells appeared to be relatively more epithelial-like and formed “cobble-stone”-like monolayers with some overlapping board-spindly cells (Right bright-field images: Fig 1 and S2 Fig). Also, reflected in the bright-field images of the four cultures, which were plated at the same time and in the same numbers, were differences in growth-rates between the lung-231 and lymph node-231 cell lines and between these two cell lines relative to the parental and 1°-tumor cell line (Lower panels of Fig 1). Thus, the lung-231 cells had the lowest growth-rate (36 hr: Lower panels of Fig 1), which is visible as a sparser covering of the plastic in the bright-field images, along with an apparently higher death-rate/senescence as indicated by the stable or plateauing/declining growth-rate by day 4 and 5. In contrast, the lymph node-231 cells had the fastest growth rate (22 hr: Lower panels of Fig 1) and these cultures (in the bright-field images) appeared to be nearly confluent relative to the other three cultures. We previously reported similar differential growth-rates in growth-rate comparisons for cultures of 435 isogenic cell lines (S3 Fig) [12]. Overall, the characteristics of the *in vitro* growth of all the isogenic cell lines has indicated that fundamental underlining inherent molecular changes had occurred during *in vivo* growth and, at least during these early passages on plastic, these differential characteristics endured as reflected in differing morphologies and growth-rates.

### General “omics”

Other than for the principle component analysis (PCA) and hierarchical clustering analyses (indicated below), we focused on the expression level fold changes (FCs: range -1.25 to +1.25) of the individual proteins or transcripts (genes) or aqueous metabolites from each isogenic metastatic cell line relative to their cognate counterparts from the 1° tumors. Our hypothesis is that metastatic progression, starting from the 1° tumor, is an evolutionary process that evolves *in situ* at each specific tissue site under the influence of inherent microenvironmental signaling and site-specific selection pressures. As such, we have been interested in understanding the fundamental molecular (proteomic and genetic) and associated metabolic (biochemical) changes that metastatic cells have undergone relative to their 1° tumor. Consequently, for proteomics (S1–S6 Spreadsheets) we attained, from a total of 6500 FCs for all isogenic cell lines, the following numbers of proteins with FCs ≤ -1.25 of: 1189 for brain-435, 805 for liver-435, 940 for lung-435, 606 for spine-435, 1076 for lung-231, and 1563 for lymph node-231 and with FCs ≥ +1.25 of: 680 for brain-435, 255 for liver-435, 735 for lung-435, 316 for spine-435, 1414 for lung-231, and 1197 for lymph node-231 cell lines. Similarly, from RNAseq (transcriptomic) analyses we had a total of ~16000 (range: 15945–16375) for all isogeneic metastatic cell lines of which transcripts (S7–S12 Spreadsheets) with FCs ≤ -1.25 were: 541 for brain-435, 1285 for liver-453, 3075 for lung-435, 1125 for spine-435, 2484 for lung-231, and 3103 for lymph node-231 cell lines and FCs ≥ +1.25 were: 1205 for brain-435, 1823 for liver-435, 3061 for lung-435, 1108 for spine-435, 2374 for lung-231, and 3785 for lymph node-231 cell lines. For aqueous metabolites we found metabolites with FCs ≤ -1.25 of: 277 for brain-435, 309 for liver-435, 249 for lung-435, and 303 for spine-435 cell lines and FCs ≥ +1.25 of: 56 for brain-435, 109 for liver-435, 151 for lung-231, and 647 for spine-435 cell lines.

### Proteomic-based PCA and proteomic- transcriptomic-based hierarchical clustering

**Fig 2:**
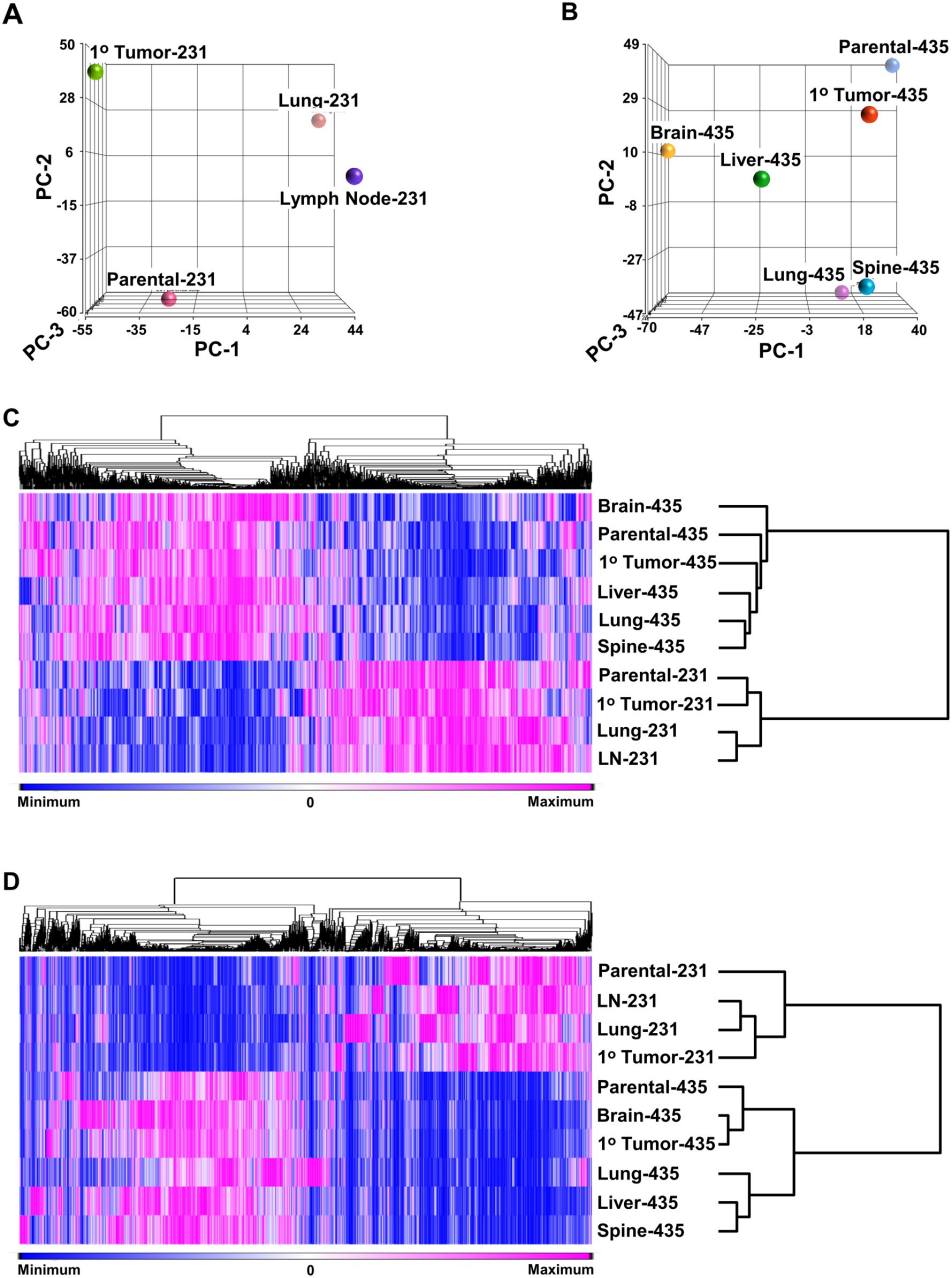
Principle component analyses (PCAs) and hierarchical clustering of all cell lines. (A) Proteomic-based PCA plots of 231 isogenic cell lines (PC-1, PC-2, and PC-3 represented 44.1, 37.0, and 18.9% of the respectively) and (B) 435 isogenic cell lines (PC-1, PC-2, and PC-3 represented 29.0, 25.7, and 21.5% of the respectively). (C) Proteomic-based hierarchal clustering (heat map) of six 435 isogenic cell lines along with four 231 isogenic cell lines. (D) Transcriptomic-based hierarchal clustering of all cell lines. All analyses indicated that each cell line had distinct proteomic/transcriptomic signatures, which resulted in the cell lines’ clustering into separate groups/clades. As shown beneath the heat maps, proteins (C) or transcripts (D) distributed across rows have been presented as gradations of color from dark blue-to-dark pink, i.e., relative minimal-to-maximal expression levels. Thus, each row of proteins (C) or transcripts (D) has been placed the left of the cell line designations and the associated trees is at the right.

Similar to our previous metabolomic and Raman spectroscopic based PCA and hierarchical clustering analyses, proteomic-based PCA indicated that 231 isogenic cell lines (Fig 2A) as well as 435 isogenic cell lines (Fig 2B) were separated into distinct tissue defined clusters, which was an indication that each isogenic cell line differs from its isogenic counterparts at the proteome level. The heat map shown in Fig 2C is complementary evidence that all isogenic cell lines have distinct proteomes. Thus, similar to the relative distances indicated in Fig 2B and 2C indicates that the proteome of parental-435 cells was most closely related to that of the 1° tumor-435 with the latter being more closely related to the liver-435 cell line, while the proteomes of the spine-435 and lung-435 cell lines formed a subclade and that brain-435 cells had a proteome that was least related to the other isogenic family of cell lines. However, Fig 2C shows that the parental-231 and 1° tumor-231cells formed a subclade, which was not apparent from Fig 2A, but, as in Fig 2A, the lymph node-231 (LN-231) and lung-231 cell lines were closely related relative to their distance from the parental-231 and 1° tumor-231 cell lines’ subclade. From Fig 2C it was found that the proteomes of the 435 cell lines and 231 cell lines formed two distinct general separate clades that was likely due to their parental cells being from different individuals. This is also likely why the two lung proteomes were not closely related, which provided some evidence that proteomes from distinct individuals remain generally discrete even after growth within very similar microenvironments.

The transcriptomic-based clustering analysis depicted in Fig 2D is complementary to Fig 2C and again indicates that the 435 cell lines and 231 cell lines formed two distinct general separate clades. In addition, the two 231 isogenic metastatic cell lines (lung-231 and lymph node (LN)-231) had, similar to their proteomes, transcriptomes that were closely related while in this case, unlike the proteomic-based analysis (Fig 2C), the parental-231 and 1° tumor-231 cell line transcriptomes were more distantly related. A similar distinction between proteomic-based and transcriptomic-based clustering was exhibited by the parental-435 and 1° tumor-435 cell lines with the latter forming a subclade with brain-435 and these three grouped separately from the lung-, liver- and spine-435 cell lines with the latter two of these forming a subclade. Hence, although proteomic-based and transcriptomic-based PCA/hierarchical analyses gave consistent complementary result with respect to providing evidence that each cell line display distinct transcriptomes and proteomes there was not an exact match in clustering patterns between to the two data sets. This was consistent with the known phenomena that proteomic and transcriptomic data sets do not generally exhibit large expression overlaps between transcripts with their protein products [17, 18, 20], which has been described as being due to a variety of regulatory distinctions associated with mRNAs and proteins [15, 16, 18, 20–25].

### Pathway discovery analyses

#### Proteome-based pathway discovery

The lists of proteins from each isogenic metastatic cell line with FCs ≤ -1.25 and ≥ +1.25, relative to their 1° tumors (S13–S18 Spreadsheets) were loaded into the ConsenusPathDB online interactive pathway discovery tool. The ConsensusPathDB integrates a total of 11 human databases for the pathway discovery analyses and we used the default setting of 2 interacting proteins to define a pathway.

**Fig 3:**
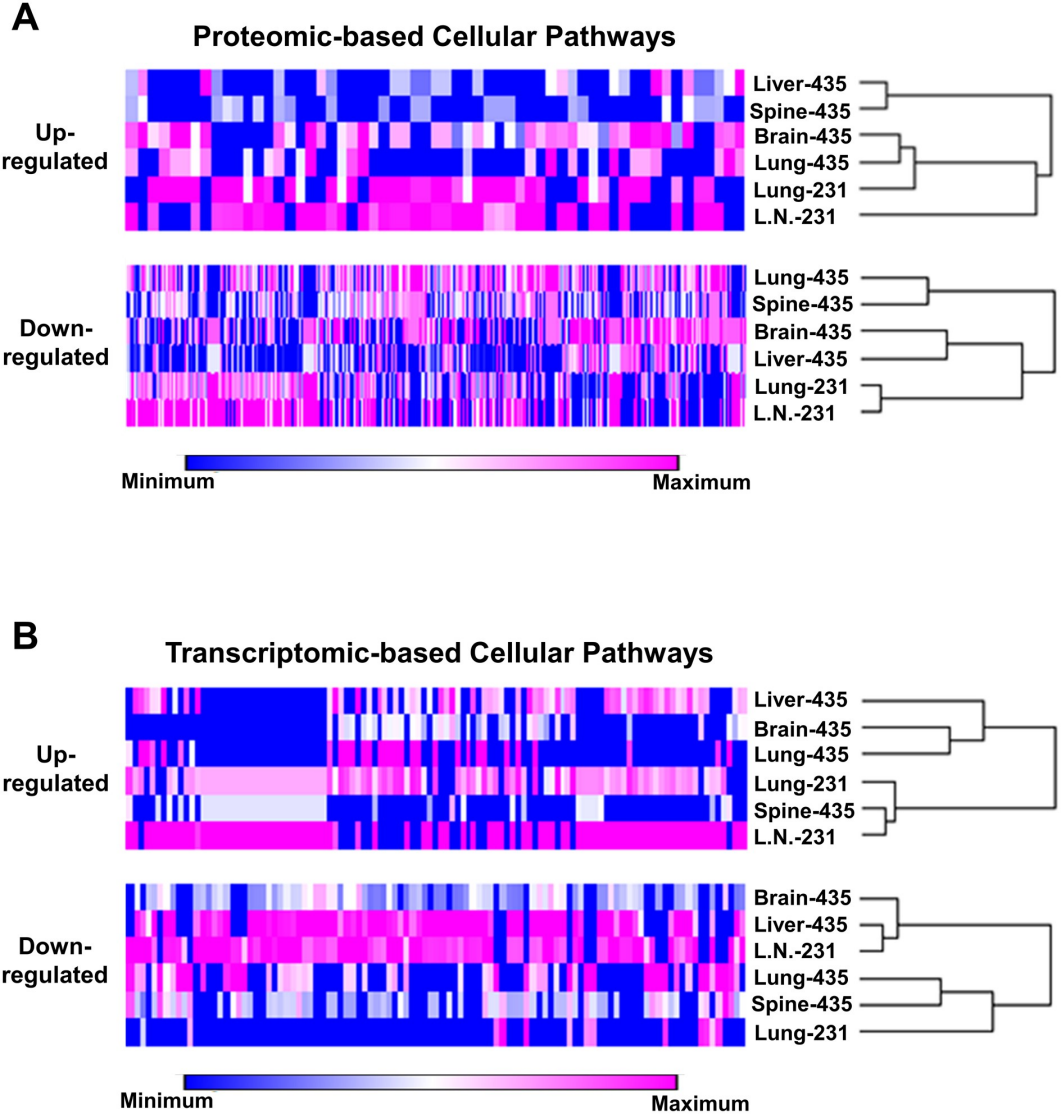
Cellular pathway hierarchical clustering’s of isogenic metastatic cell lines. (A) Proteomic-based up- and down-regulated pathway clustering’s. (B) Transcriptome-based up- and down-regulated pathway clustering’s. As shown beneath the heat maps (colored bar), pathways were distributed across rows and shown as gradations of color from dark blue-to-dark pink, i.e., relative minimal-to-maximal expression levels. Each row of pathways is to the left of the cell line designations and the associated tree is at the right.

In order to find out how the proteomic-based pathway analyses could be used to find how the isogenic metastatic cell lines were related, we submitted the lists of up- and down-regulated pathways to hierarchical clustering analysis and the resulting heat maps are shown in Fig 3A. These analyses showed that both the up- and down-regulated pathway data sets, separated the isogenic cell lines into two, in broadest terms, clades of 435 and 231 metastatic cell lines with the lung-231 and lymph node-231 cell lines closely related, although in the up-regulated set the lymph node-231 cells formed a somewhat ‘outside’ separate grouping while in the down-regulated set the lung-231 and lymph node-231 cell lines were highly related. In the case the 435 cell lines, based on the up-regulated pathways, the liver-435 and spine-435 were closely related and grouped into one subclade while the brain-435 and lung-435 cell lines formed another subclade. However, this patterned differed in the down-regulated heat map where lung-435 grouped with spine-435 while brain-435 and liver-435 were grouped together.

Abridged data sets, i.e., the top 10 up- and down-regulated pathways for each isogenic metastatic cell line, ranked on the lowest to highest q-values, i.e., adjusted p-values, (q ≤ 0.05) are provided in: S1–S6 Tables with the complete pathway lists given in S19 Spreadsheet. The abridged pathway lists provided a means of exploring examples of trends found in the complete pathway lists. The integration of 11 Source databases used in ConsensusPathDB analyses provided built-in consistency/verification controls in that 2 or more Sources (databases) often identified the same pathways even though the discovery of the pathway by each Source is based on different protein list processing algorithms and different statistical criteria [26]. For example, in: S1 Table, within the top 10 up-regulated pathways the ‘citric acid cycle’ (Source: Reactome) was also given as the ‘TCA cycle’ (Source: Wikipathways). Similarly, in the same table within the top 10 down-regulated pathways, ‘pyrimidine metabolism’ (Source: Wikipathways) was repeated (Source: KEEG). Another example is exhibited in the 10 top down-regulated pathways of: S4 Table, where ‘glycolysis’ (Source: HumanCyc) is repeated (Source: Reactome) as well as, under different descriptive titles, twice more (Sources: INOH & Wikipathways). A disadvantage is that all the Sources include very ill-defined vague general pathway terms. For example, in S1 Table the designated: ‘amino acid metabolism’ pathway (in the top 10 up-regulated list) leads one to consider a variety of metabolic pathways; e.g., ranging from catabolism, to several types of modifications as well as the incorporation into nascent protein chains. Similarly (same table), in the top 10 down-regulated list is the ‘cell cycle’ pathway with its set of 564 proteins, which was also represented as several more specific sub-pathways: ‘cell cycle, mitotic’, ‘cell cycle checkpoints’, ‘mitotic spindle checkpoints’, and ‘mitotic anaphase’. Other examples of such broad pathway designations include: ‘vesicle-mediated transport’ (S2 Table), ‘metabolism’ as well as ‘hemostasis’ (S3 Table), ‘cellular responses to stress’ along with ‘metabolism of carbohydrates’ (S4 Table), and ‘metabolism of RNA’, ‘ribosome’ and ‘innate immune system’ (S5 Table). Nevertheless, many pathways listed (S1–S6 Tables) were relatively specific. Moreover, the pathway analyses allowed for an inspection of up- and down-regulated pathways that were common to 2 or more metastatic sites and thus, provided a means to assess those pathways that promote general metastatic processes, regardless of being up- or down-regulated. The abridged datasets already include several examples of shared pathways, such as up-regulated ‘spliceosome’ of brain-435 and lymph node-231 and very closely related ‘mRNA splicing—major pathway’ along with ‘mRNA splicing’ of lung-231, up-regulated ‘lysosome’ brain-435 and liver-435, down-regulated ‘cell cycle’ of brain-435 and liver-435, down-regulated ‘pyrimidine metabolism’ brain-435 and liver-435, up-regulated ‘TCA cycle’ of brain-435 and the very closely related ‘TCA cycle & respiratory electron transport’ of lung-435, up-regulated ‘metabolism of RNA’, ‘cell cycle’, and ‘cell cycle, mitotic’ of lung-231 and lymph node-231, and down-regulated ‘EGFR1’, ‘neutrophil degranulation’, ‘metabolism’, ‘vesicle-mediated transport’, ‘membrane trafficking’ as well as ‘post-translational protein phosphorylation’ of lung-231 and lymph node-231. Also, there were examples of pathways that were up-regulated at one site while being down-regulated at other sites. Examples, (S1–S6 Tables) are: up-regulation of ‘insulin-like growth factor (IGF) transport & uptake by IGF binding proteins (IGFBPs)’ and ‘neutrophil degranulation’ in liver-435 and the down-regulation of both pathways in lung-435 and the former is in lymph node-231 and the latter is in spine-435, lung-231, and lymph node-231.

At the same time, pathway analysis allowed for the discovery of up- and down-regulated pathways that are unique to each metastatic site, which provides insights into how the cells may have evolved by adapting attributes of their site of growth that would have been induced by signaling cascades that were inherent to each tissue. Similar to the analysis of all pathways given above, the top 10 unique up-regulated and top 10 unique down-regulated proteome based pathways for each isogenic metastatic cell line, ranked on the lowest to highest q-values (q ≤ 0.05), are provided in Tables 1–6 with the complete pathway lists given in S19 Spreadsheet. The general top 10 summaries shown in Tables 1–6 indicate that: in brain-435 cells the ‘mitochondrial TCA’ pathway was up-regulated, which may have occurred either as a response to energy needs, i.e., as an energy associated pathway via metabolism of glucose to pyruvate (see the listed superpathway) and then to acetyl-CoA for use in the TCA cycle or was up-regulated in response to an anaplerosis need, the ‘mitochondrial fatty acid β-oxidation’ (energy generating) pathway was also up-regulated while a down-regulation of mitochondrial biogenesis and DNA repair pathways was found for brain-435; in the liver-435 cell line relatively liver-specific pathways were up-regulated including ‘fibrin clot formation’, general ‘hemostasis’, heparan sulfate-glcNAc-glcA (HS-GAG) degradation along with scavenger pathways while ‘mitotic checkpoint’, glucose uptake, apoptosis, and ‘RNA metabolism’ pathways were down-regulated; in lung-435 cells components of the innate immune system (lung, similar to skin, must safe-guard against airborne pathogens), i.e., interferon signaling pathways, were up-regulated along with oxidative phosphorylation pathways, which were likely induced by the high O_2_ tension of the lung, as well as disease pathways, such as Parkinson’s, Alzheimer’s, *etc*., which have been associated with mitochondrially generated reactive oxygen species while down-regulations included the translation (EIF-4e) but proapoptotic (p70s6) pathway, the hypoxia driven VEGF pathway, which was likely due to the high O_2_ tension of the lung as well as estrogen and androgen signaling; the spine-435 cell line had an up-regulation of histone modification, amino acid and oligopeptide solute transport, ‘terpenoid backbone biosynthesis’, ‘FOXA1 transcription factor network’, with a down-regulation of several toll-like receptor (innate immune response) pathways; the pathways up-regulated in lung-231 cells were several involved with translation including mitochondrial translation and DNA repair, with down-regulated pathways involved with extracellular matrix degradation, apoptosis, IL-7 (hematopoietic growth factor), epidermal growth factor (EGF) signaling, and stress induced heat shock protein; in the lymph node-231 cell line RUNX3 transcriptional regulation and HDAC Class I signaling were up-regulated along with up-regulated ‘hematopoietic stem cell regulation by GABP-α/β complex’, which might reflect influences on the cells during lymph node site growth as these pathways are involved with regulation of hematopoietic lineages, RHO GTPases, ‘EPHA-mediated growth cone collapse’, ‘TGF-β receptor’, and ‘glucocorticoid receptor regulatory network’ were also up-regulated with ‘mitochondria β-oxidation of short chain saturated fatty acids’, ‘urea cycle and metabolism of arg, pro, glu, asp, & asn’, and one-carbon metabolism were down-regulated.

**Table 1:** Proteomic-based unique pathways for the metastatic brain-435 cell line.

Source	Up Pathways	# of Proteins in Set	# of Obs. Proteins	Obs. Proteins (%)	q-value
Reactome	Citric Acid Cycle	22	7	31.8	0.0022
Wikipathways	TCA Cycle	17	6	35.3	0.0030
SMPDB	Malonyl-CoA Decarboxylase Deficiency	14	5	35.7	0.0034
SMPDB	Malonic Aciduria	14	5	35.7	0.0034
SMPDB	Methylmalonic Aciduria Due to Cobalamin-Related Disorders	14	5	35.7	0.0034
Wikipathways	Metabolic Reprogramming in Colon Cancer	42	8	19.0	0.0035
HumanCyc	Superpathway of Conversion of Glucose to Acetyl CoA & Entry into the TCA Cycle	48	8	17.0	0.0064
BioCarta	IGF-1 Receptor & Longevity	17	5	29.4	0.0071
Reactome	Mitochondrial Fatty Acid β-Oxidation	39	7	18.4	0.0085
Reactome	Clathrin-mediated Endocytosis	138	14	10.1	0.0090
	**Down Pathways**				
PID	ATR Signaling Pathway	37	15	40.5	1.57E-06
PID	PLK1 Signaling Events	44	16	36.4	2.26E-06
Reactome	Mitochondrial Translation Termination	89	22	25.0	6.10E-06
Reactome	Mitochondrial Translation Elongation	89	22	25.0	6.10E-06
Reactome	Mitochondrial Translation Initiation	89	22	25.0	6.10E-06
Reactome	Mitochondrial Translation	95	22	23.4	1.79E-05
PID	Fanconi Anemia Pathway	46	14	30.4	8.58E-05
KEGG	Hepatitis C	155	27	17.4	0.000209
Reactome	DNA Repair	320	43	13.6	0.000269
Reactome	Interleukin-27 Signaling	10	6	60.0	0.000451

**Table 2:** Proteomic-based unique pathways for the metastatic liver-435 cell line.

Source	Up Pathways	# of Proteins in Set	# of Obs. Proteins	Obs. Proteins (%)	q-value
Reactome	Regulation of IGF Transport & Uptake by IGFBPs	127	11	8.7	0.000201
Reactome	Formation of Fibrin Clot	39	6	15.4	0.000953
Reactome	Post-translational Protein Phosphorylation	110	9	8.3	0.001048
Reactome	Hemostasis	668	22	3.3	0.003980
PID	Arf1 pathway	20	4	20.0	0.004781
Reactome	HS-GAG Degradation	21	4	19.0	0.005157
Reactome	Intrinsic Pathway of Fibrin Clot Formation	22	4	18.2	0.005891
Reactome	Binding and Uptake of Ligands by Scavenger Receptors	41	5	12.2	0.006087
Reactome	Platelet Degranulation	129	8	6.2	0.008542
PID	FOXA2 and FOXA3 Transcription Factor Networks	45	5	11.1	0.008542
	**Down Pathways**				
Reactome	Cds1 Mediated Inactivation of Cyclin B:Cdk1 Complex	13	8	61.50	8.10E-06
PID	Regulation of Nuclear β-Catenin Signaling & Target Gene Transcription	80	17	21.20	2.46E-05
PID	Insulin-mediated Glucose Transport	29	10	34.50	5.01E-05
Reactome	Activation of BAD & Translocation to Mitochondria	15	7	46.70	0.0001761
PID	p38 Signaling Mediated by MAPKAP Kinases	21	8	38.10	0.000203
Reactome	Protein Folding	103	17	16.50	0.000364
Reactome	Translocation of GLUT4 to the Plasma Membrane	32	9	28.10	0.000560
Reactome	Metabolism of RNA	586	51	8.70	0.000747
KEGG	Drug Metabolism—Other Enzymes	79	14	17.70	0.000747
PID	LKB1 Signaling Events	43	10	23.30	0.000910

**Table 3:** Proteomic-based unique pathways for the metastatic lung-435 cell line.

Source	Up Pathways	# of Proteins in Set	# of Obs. Proteins	Obs. Proteins (%)	q-value
Reactome	Interferon Signaling	158	31	19.6	1.41E-10
KEGG	Parkinson disease	142	27	19.0	3.12E-09
KEGG	Nonalcoholic Fatty Liver Disease	149	26	17.4	3.78E-08
Wikipathways	Nonalcoholic Fatty Liver Disease	155	26	16.8	7.95E-08
KEGG	Alzheimer Disease	171	26	15.2	4.94E-07
KEGG	Oxidative phosphorylation	133	22	16.5	1.39E-06
Wikipathways	Electron Transport Chain (OXPHOS)	103	19	18.4	2.07E-06
KEGG	Epstein-Barr Virus Infection	201	27	13.5	2.42E-06
KEGG	Huntington Disease	193	26	13.5	4.32E-06
Reactome	Interferon-α/β Signaling	70	15	21.4	6.76E-06
	**Down Pathways**				
BioCarta	Regulation of EIF-4e & p70s6 Kinase	25	10	40.0	2.11E-05
Reactome	Signaling by VEGF	100	18	18.0	0.0002624
KEGG	Estrogen Signaling Pathway	137	21	15.4	0.0004490
Wikipathways	4-Hydroxytamoxifen, Dexa-methasone, & Retinoic Acids Regulation of p27 Expression	17	7	41.2	0.000475
PID	AMB2 Integrin Signaling	31	9	29.0	0.000701
Reactome	Regulation of PTEN Stability & Activity	25	8	32.0	0.000819
BioCarta	Corticosteroids & Cardioprotection	27	8	29.6	0.001382
KEGG	Prostate Cancer	97	16	16.5	0.001407
Wikipathways	Androgen Receptor Signaling Pathway	89	15	16.9	0.001709
BioCarta	VEGF Hypoxia & Angiogenesis	30	8	26.7	0.002542

**Table 4:** Proteomic-based unique pathways for the metastatic spine-435 cell line.

Source	Up Pathways	# of Proteins in Set	# of Obs. Proteins	Obs. Proteins (%)	q-value
Wikipathways	Ethanol Effects on Histone Modifications	31	5	16.1	0.0095
Reactome	Amino Acid Transport Across the Plasma Membrane	32	5	15.6	0.0095
KEGG	Terpenoid Backbone Biosynthesis	22	4	18.2	0.0095
Wikipathways	mRNA, Protein, & Metabolite Inducation Pathway by Cyclosporin A	6	3	50.0	0.0095
PID	FOXA1 Transcription Factor Network	44	5	11.4	0.0154
HumanCyc	Superpathway of Geranyl- geranyldiphosphate Biosynthesis I (*via* Mevalonate)	12	3	25.0	0.0169
HumanCyc	Eumelanin Biosynthesis	4	2	50.0	0.0279
Reactome	Amino Acid & Oligopeptide SLC Transporters	52	5	9.6	0.0282
Wikipathways	Type 2 Papillary Renal Cell Carcinoma	34	4	11.8	0.0363
Reactome	LRR FLII-Interacting Protein 1 Activates Type I IFN Production	5	2	40.0	0.0371
	**Down Pathways**				
Reactome	MyD88 Cascade Initiated on Plasma Membrane	87	12	13.8	0.001695
Reactome	Toll Like Receptor 10 (TLR10) Cascade	87	12	13.8	0.001695
Reactome	TLR5 Cascade	87	12	13.8	0.001695
Reactome	TRAF6 Mediated NFκB & MAP Kinases *via* TLR7/8 or 9	88	12	13.6	0.001829
Reactome	TLR9 Cascade	94	12	12.8	0.002692
Reactome	MyD88:Mal Cascade	97	12	12.4	0.003160
Reactome	TLR1:TLR2 Cascade	97	12	12.4	0.003160
Reactome	TLR6:TLR2 Cascade	97	12	12.4	0.003160
Reactome	TLR2 Cascade	97	12	12.4	0.003160
Reactome	TLR4 Cascade	127	14	11.0	0.003303

**Table 5:** Proteomic-based unique pathways for the metastatic lung-231 cell line.

Source	Up Pathways	# of Proteins in Set	# of Obs. Proteins	Obs. Proteins (%)	q-value
Reactome	Translation	310	71	23.1	4.28E-15
KEGG	Ribosome	153	45	29.4	2.92E-13
Reactome	Mitochondrial translation	95	32	34.0	2.91E-11
Reactome	Nonsense Mediated Decay (NMD) Enhanced by the Exon Junction Complex (EJC)	118	33	28.2	2.33E-09
Reactome	Nonsense-Mediated Decay	118	33	28.2	2.33E-09
Reactome	Eukaryotic Translation Elongation	106	31	29.5	2.46E-09
Wikipathways	Cytoplasmic Ribosomal Proteins	88	27	30.7	1.34E-08
Reactome	Eukaryotic Translation Termination	104	29	28.2	2.63E-08
Reactome	Selenoamino Acid Metabolism	130	33	25.6	2.63E-08
Reactome	NMD Independent of the EJC	106	29	27.6	4.13E-08
	**Down Pathways**				
Reactome	Degradation of the Extracellular Matrix	105	17	16.2	0.005843
PID	p75(NTR)-Mediated Signaling	71	13	18.6	0.007076
BioCarta	Inhibition of Matrix Metalloprotein-ases	8	4	50.0	0.012534
Signalink	EGF-Core	105	16	15.	0.012969
KEGG	Apoptosis	136	19	14.0	0.013516
Wikipathways	Nanomaterial Induced Apoptosis	20	6	30.0	0.015117
NetPath	IL-7	27	7	25.9	0.015360
BioCarta	Stress Induction of HSP Regulation	14	5	35.7	0.015729
Reactome	Retinoid Metabolism & Transport	45	9	20.0	0.020881
SMPDB	Pyruvate Dehydrogenase Complex Deficiency	22	6	27.3	0.021846

**Table 6:** Proteomic-based unique pathways for the metastatic lymph node-231 cell line.

Source		Up Pathways		# of Proteins in Set		# of Obs. Proteins		Obs. Proteins (%)		q-value	
Reactome		Transcriptional Regulation by RUNX3		52		15		29.4		4.86E-07	
PID		Signaling Events Mediated by HDAC Class I		56		15		26.8		1.82E-06	
BioCarta		Information Processing Pathway at the IFN-β Enhancer		29		10		34.5		8.25E-06	
Reactome		Generic Transcription Pathway		1107		107		9.7		1.14E-05	
Reactome		RHO GTPases Activate CIT		19		8		42.1		1.23E-05	
NetPath		TGF-β Receptor		174		27		15.6		1.88E-05	
Reactome		EPHA-mediated Growth Cone Collapse		15		7		46.7		1.95E-05	
PID		Glucocorticoid Receptor Regulatory Network		80		16		20.0		4.68E-05	
Wikipathways		Hematopoietic Stem Cell Gene Regulation by GABP-α/β Complex		19		7		36.8		0.000121	
Reactome		RHO GTPases Activate ROCKs		19		7		36.8		0.000121	
		**Down Pathways**									
SMPDB		MIT β-Oxidation of Short Chain Saturated Fatty Acids		8		6		75.0		0.000302	
SMPDB		Short-chain 3-hydroxyacyl-CoA Dehydrogenase Deficiency		8		6		75.0		0.000302	
Reactome		COPI-mediated Anterograde Transport		83		19		22.9		0.001986	
EHMN		Urea Cycle & Metabolism of Arg, Pro, Glu, Asp & Asn		106		22		21.0		0.002350	
Wikipathways		One Carbon Metabolism & Related Pathways		52		14		26.9		0.002435	
EHMN		3-Oxo-10R-octadecatrienoate β-oxidation		11		6		54.5		0.002649	
SMPDB		3-Methylglutaconic Aciduria Type I		30		10		33.3		0.002710	
SMPDB		2-Methyl-3-Hydroxybutryl CoA Dehydrogenase Deficiency		30		10		33.3		0.002710	
SMPDB		Isovaleric Aciduria		30		10		33.3		0.002710	
SMPDB		3-Methylcrotonyl CoA Carboxylase Deficiency Type I		30		10		33.3		0.002710	

**Fig 4:**
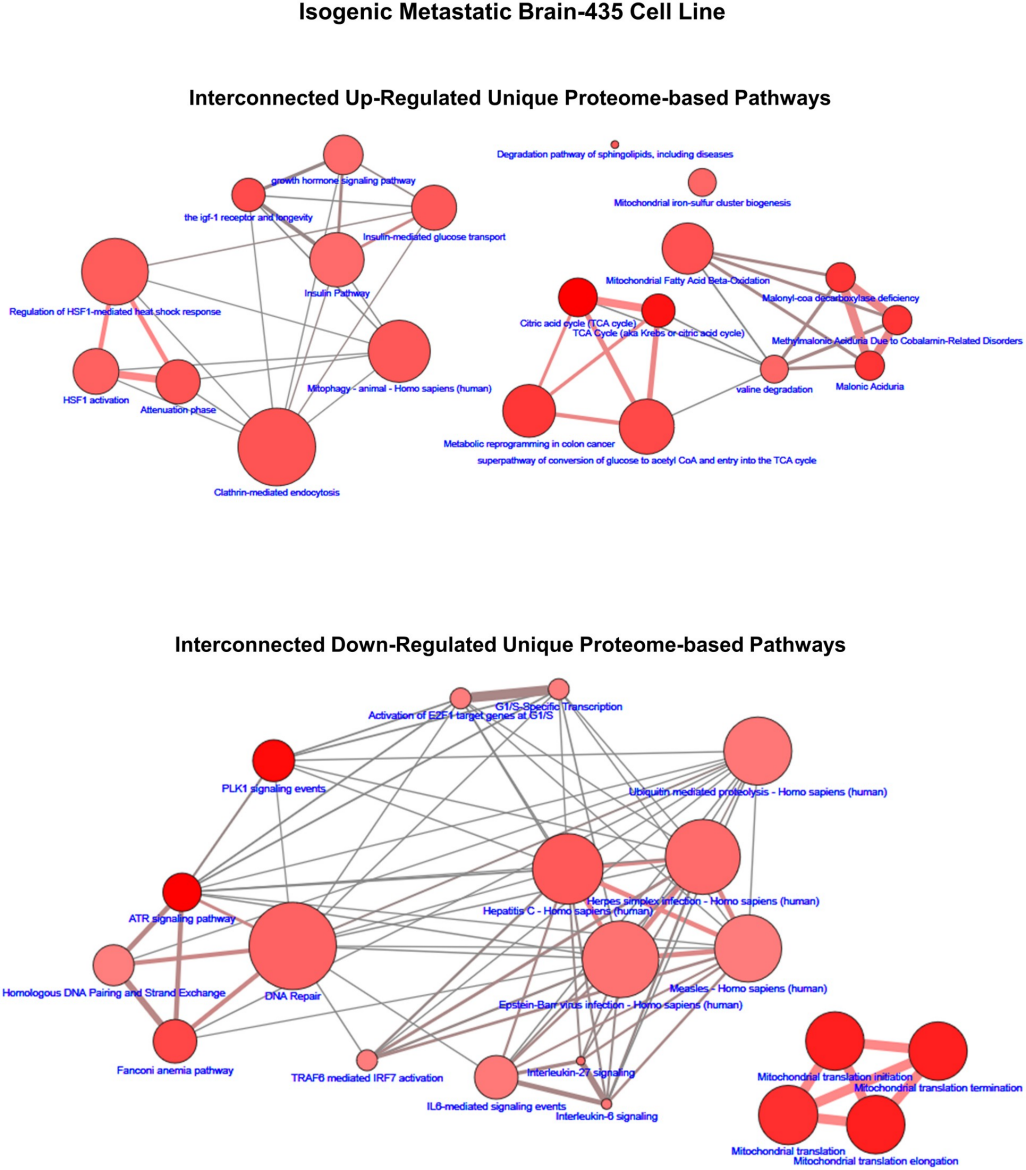
The up- and down-regulated proteomic-based interconnected network maps of pathways unique to the brain-435 cell line. The size range of the nodes correlates to the size of the protein sets while the range of hues of the nodes correlates with the q-values, which is correlated to the size of the number of observed proteins. The edges represent the overlap of shared proteins between the connected nodes with the width of the edges representative of the size of the overlap and their color denoting the number of the observed proteins that are shared.

**Fig 5:**
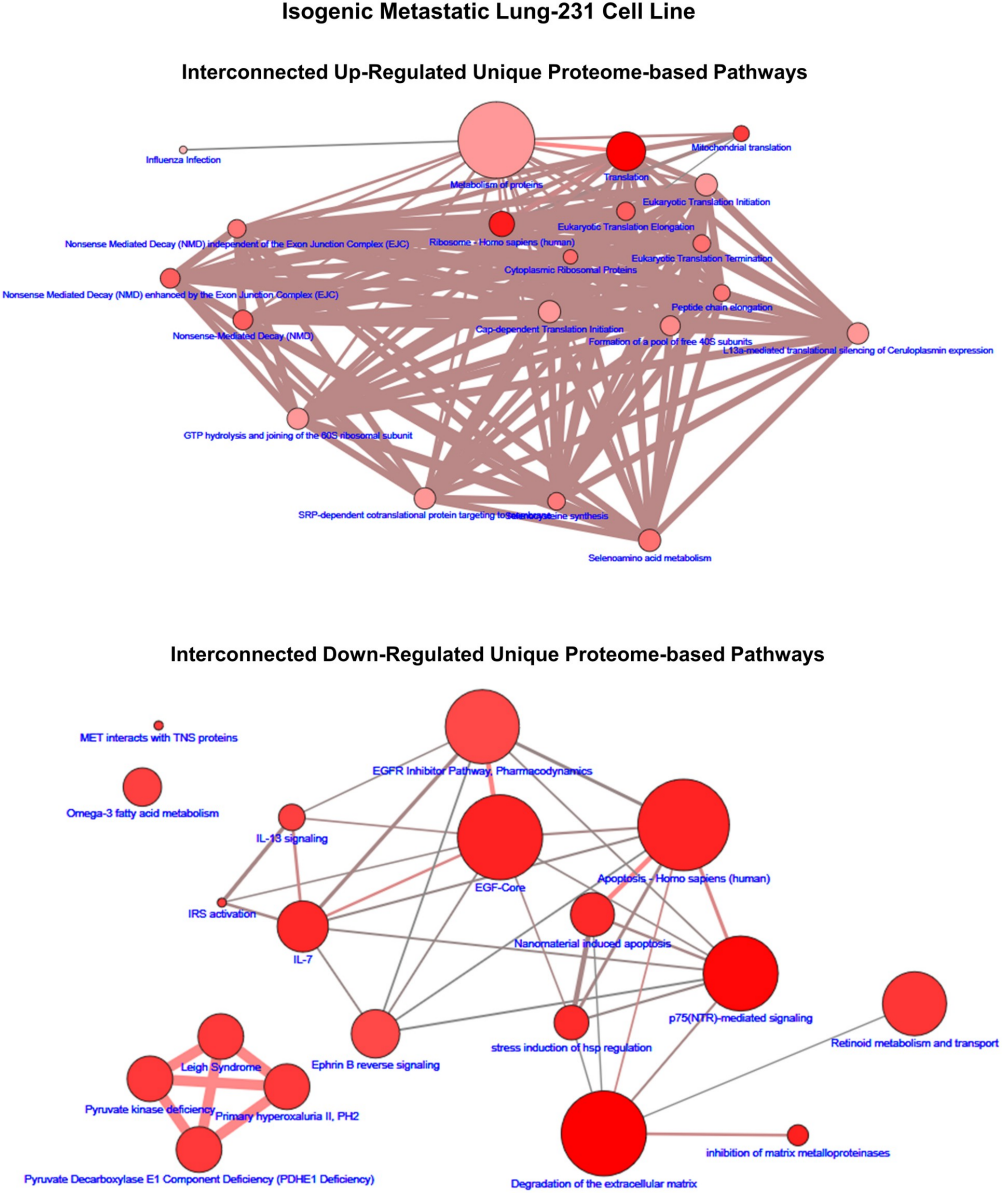
The up- and down-regulated proteomic-based interconnected pathway network maps of unique to the lung-231 cell line. The size range of the nodes correlates to the size of the protein sets while the range of hues of the nodes correlates with the q-values, which is correlated to the size of the number of observed proteins. The edges represent the overlap of shared proteins between the connected nodes with the width of the edges representative of the size of the overlap and their color denoting the number of the observed proteins that are shared.

In order to gain an appreciation that the lists of proteome pathways are not isolated independent biochemical reaction entities but rather are interconnected systems, we leveraged the ConsensusPathDB analysis program to construct overlapping pathway interconnection maps [26, 27]. This analysis revealed that due to the multifunctional attribute of one or more of the proteins of a pathway two or more pathways have shared proteins that connect pathways into larger networks of biochemical reaction systems with overlapping functions. Examples of such maps are shown in Fig 4 (brain-435) and Fig 5 (lung-231) along with Supplemental Information: S4–S7 Figs that include, for visualization purposes, only the top 20 up- and down-regulated unique pathways (q ≤ 0.05) for each isogenic metastatic cell line. Figs 4 and 5 are representative of the examples presented in S4–S7 Figs and depict, in example Fig 4 of the unique up-regulated proteomic-based pathways of brain-435 two interconnected mappings of 18 pathways with two pathways left disconnected from either of these, i.e.,‘orphan’ pathways. In this example, one of the interconnected maps (Left-hand side, Fig 4) was dominated by heat shock factor (HSF1) associated pathways with a relatively high amount of overlapping protein components and is loosely connected with an insulin/growth hormone signaling group of pathways that have fewer overlapping protein partners. On the right-hand side of the upper portion of Fig 4 there emerged a clustered network of strongly associated TCA pathways connected via a valine degradation pathway to a group of malonate/vitamin B12 tightly associated pathways and a relatively high amount of proteins with overlapping functions in a mitochondrial fatty acid β-oxidation pathway. In the down-regulated unique protein network map of Fig 4 two independent networks emerged: a mitochrondrial translation network (lower right-hand side) along with a complex network composed of least 3–4 relatively strongly overlapping pathway networks: the interleukin associated pathways (lower right), the DNA repair pathways (lower left), the disease/infection associated pathways (central right), and the G1/S pathways (upper center). Similar examples of interconnected pathway relationships can be discerned in the pathways shown in Fig 5 as well as in the S4–S7 Figs.

#### Transcriptome-based pathway discovery

Analogous to the proteome-based analyses, lists of transcripts from each isogenic metastatic cell line with FCs ≤ -1.25 and ≥ +1.25, relative to their 1° tumors (S7–S12 Spreadsheets) were analyzed with ConsenusPathDB. Abridged data sets, i.e., the top 10 up- and down-regulated pathways for each isogenic metastatic cell line, (q ≤ 0.05) are provided in S7–S12 Tables with the complete pathway lists given in S20 Spreadsheet. Although transcriptome-based pathway discovery analyses uncovered pathways common with those of the proteome-based pathways (see below), overall, the majority of transcriptome-based pathways differed from the above proteome-based pathways.

Similar to the proteomic based pathway lists, we submitted the transcriptomic based lists of up- and down-regulated pathways to hierarchical clustering analysis and the resulting heat maps are shown in Fig 3B. Unlike the proteomic-based heat maps, both the up- and down-regulated pathway data sets of these analyses separated the lung-231 and lymph node-231 cell lines. In the case of the up-regulated heat map the lung-231 cell line was linked to a clade made up of closely related spine-435 and lymph node-231 cell lines while at the same time being closer to the lung-435 cell line that formed a clade with the brain-435 cell line with this clade being more distantly linked to the liver-435 cell line. In the case of the down-regulated heat map, lung-231 was linked to the clade of closely related spine-435 and liver-435 cell lines while the lymph node-231 cell line was linked in a separate clade to the liver-435 cell line and more distantly, both were linked to the brain-435 cell line.

As with the ConsensusPathDB analyses of the proteomic data sets, several of the integrated databases of ConsensusPathDB uncovered the identical or overlapping similar pathways from the submitted gene lists. For example, in metastatic brain-435 (S7 Table) the ‘ECM-receptor interaction’ pathway (Source: KEGG) was up-regulated and confirmed as the ‘extracellular matrix organization’ pathway (Source: Reactome) and (S7 Table) the down-regulated ‘DNA replication’ pathway (Source: Reactome) was replicated (Source: Wikipathways). Several of these types of examples can be seen in the S7–S12 Tables. General vague pathway designations were also again observed, such as ‘extracellular matrix organization’, ‘cell cycle’, ‘cytokine signaling in immune system’, ‘metabolism of RNA’, ‘gene expression (transcription)’, ‘RNA polymerase II transcription’, generic transcription pathway’, ‘chromatin modifying enzymes’, ‘axon guidance’, ‘muscle contraction’, and ‘neutrophil degranulation’.

The top 10 up- and down-regulated transcriptome-based unique pathways for each isogenic metastatic cell line are listed in the S13–S18 Tables. Pathway duplications or lists of similar pathways from different integrated Sources along with vague pathways designations were again obvious. Pathways that provided possible examples of metastatic cell lines acquiring tissue specific assimilations included: for brain-435 up-regulated ‘presynaptic depolarization & calcium channel opening’, ‘NCAM1 interactions’, and phenylethylamine degradation I’ pathways; for liver-435 up-regulated ‘interferon-α/β signaling’ and ‘chondroitin sulfate/dermatan sulfate metabolism’ pathways; for spine-435 (given its neuronal component) up-regulated ‘striated muscle contraction’, ‘val, leu, and Ile degradation’, ‘ketogenesis’, and ‘ion channel transport’ pathways; and, for lymph node-231 up-regulated ‘non-genomic actions of 1,25-dihydroxyvitamine D3’.

**Fig 6:**
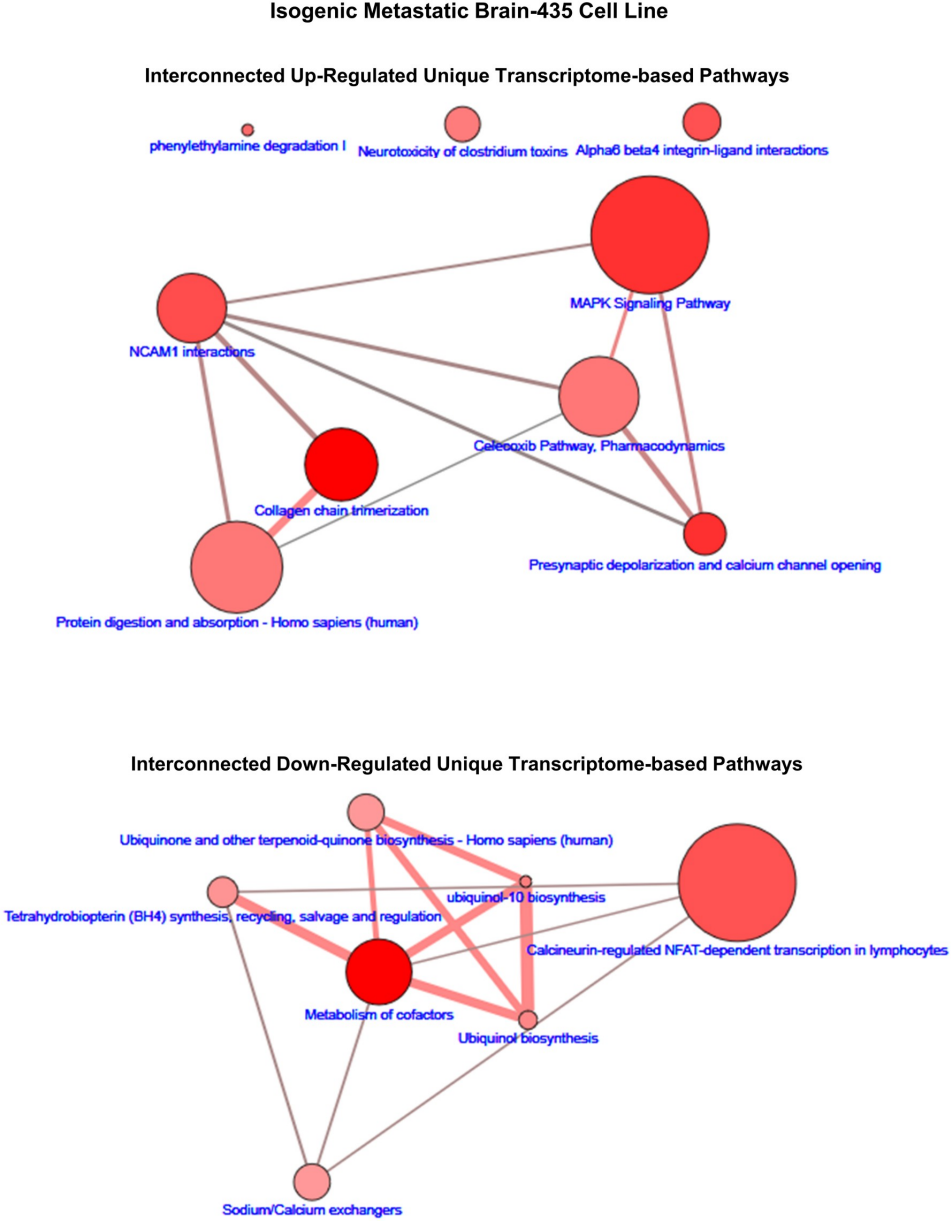
The up- and down-regulated transcriptomic-based interconnected pathway network maps of unique to the brain-435 cell line. The size range of the nodes correlates to the size of the transcript (gene) sets while the range of hues of the nodes correlates with the q-values, which is correlated to the size of the number of observed transcripts. The edges represent the overlap of shared transcripts of the connected nodes with the width of the edges representative of the size of the overlap and their color denoting the number of the observed transcripts that are shared.

**Fig 7:**
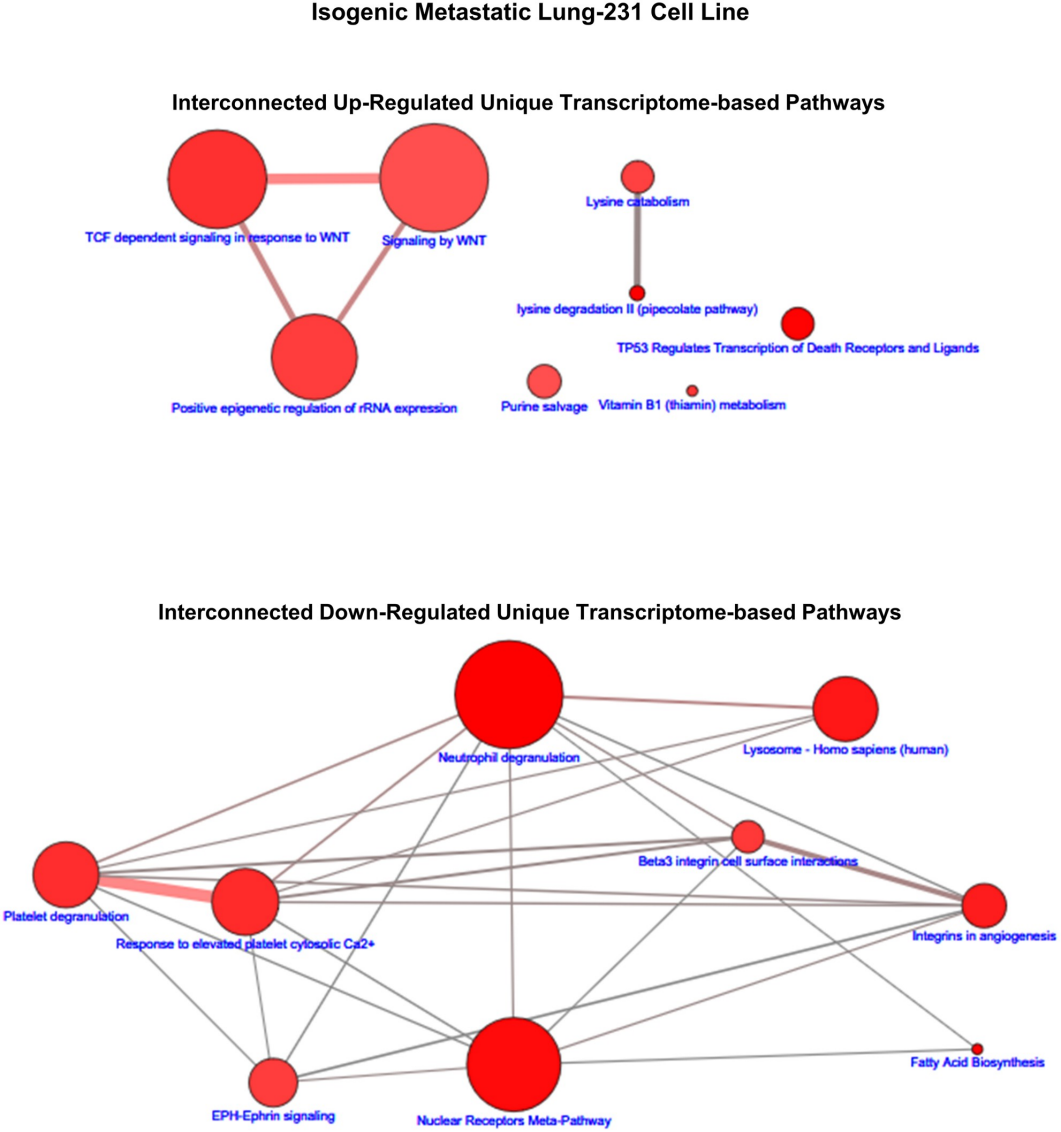
The up- and down-regulated transcriptomic-based interconnected pathway network maps of unique to the lung-231 cell line. The size range of the nodes correlates to the size of the transcript (gene) sets while the range of hues of the nodes correlates with the q-values, which is correlated to the size of the number of observed transcripts. The edges represent the overlap of shared transcripts of the connected nodes with the width of the edges representative of the size of the overlap and their color denoting the number of the observed transcripts that are shared.

Similar to the case of the proteome pathways, we explored the interconnected pathway networks formed by those transcriptome-based pathways that were unique to each isogenic cell line. Examples of such networks are shown in Fig 6 (brain-435) and Fig 7 (lung-231) as well as in S8–S11 Figs. However, in these cases the number of pathways that were significantly up- or down-regulated, i.e., q ≤ 0.05, was not always greater-than or equal to 20. Thus, for those cases with less-than 20 pathways: brain-435 cells had 9 up- and 7 down-regulated pathways (Fig 6), lung-435 cells had 11 down-regulated pathways (S9 Fig), spine-435 cells had 13 up-regulated pathways (S10 Fig), lung-231 cells had 8 up- and 9 down-regulated pathways (Fig 7), and lymph node-231 cells had 8 down-regulated (and 5 of these were trending down with q = 0.0.55; S11 Fig). Not only were there often fewer significant (q ≤ 0.05) transcriptomic pathways in the networks but, in all cases, the connections between the pathways within the networks were weaker, that is, the connections consisted of fewer shared transcript products. Moreover, several more ‘orphan’, i.e., non-connected pathways emerged during these analyses.

#### Common proteomic and transcriptomic pathways

As a means of confirming the proteome-based and transcriptome-based pathway discovery analyses, we compared the protein lists to their corresponding transcript lists (FCs ≤ -1.25 and ≥ 1.25) to match the proteins to their identical transcripts. This showed that the resulting matched protein-transcript lists were generally only a small percentage of either of their source lists, i.e., RNAseq-based transcripts had relatively few matches to the corresponding proteomic-based proteins. Hence, relative to their source protein or transcript lists, the up-regulated matched lists overlapped by only: 6.2 or 3.3%, 22.2 or 3.0%, 24.3 or 5.4%, 10.1 or 2.9%, 19.4 or 11.3%, and 25.6 or 8.1% for brian-435, liver-435, lung-435, spine-435, lung-231, and lymph node-231 respectively. Similarly, relative to their source protein or transcript lists, down-regulated matched lists overlapped by only: 6.9 or 15.4%, 10.0 or 6.3%, 17.8 or 5.3%, 12.7 or 6.8%, 38.9 or 16.9%, and 34.5 or 17.4% for brain-435, liver-435, lung-435, spine-435, lung-231, and lymph node-231 respectively. These resulting lists were submitted to ConsensusPathDB to find those pathways that were common to both “omics” analyses. Representatives of these common pathways are given as the top 10 up- and down-regulated pathways (q ≤ 0.05) in S19–S24 Tables with the complete common pathway lists given in SPT1 Spreadsheet. In the case of the lung-435 cell line we found only 5 up-regulated pathways that trended (S21 Table—gray shading: q = 0.055) as common. In addition, during all pathway discovery analyses, we used the default settings of ConsensusPathDB, which used ≥ 2 proteins to define a ‘pathway’. Consequently, several of the listed common pathways in S19–S24 Tables were composed of only a few (2–4) proteins/genes and yet remained statistically significant, i.e., q ≤ 0.05, and therefore, have been included but their relevant biological significance will require further investigations.

#### Common as well as unique proteomic-transcriptomic pathways

The top 10 up- and down-regulated pathways (q ≤ 0.05) that were common as well as unique proteome-transcriptome pathways of each isogenic metastatic cell line are presented in Tables 7–12. Examples of possible tissue specific associated pathways were those of liver-435: the up-regulated general ‘immune system’ and especially the ‘innate immune system’, ‘neutrophil degranulation’, ‘MHC class II antigen presentation’, and general ‘hemostasis’, as well as with ‘metabolism of fat-soluble vitamins’. The immune system and its sub-pathways that are involved with exogenous pathogen threats are prevalent components of the liver, which receives the largest portion of its blood supply from the portal vein and hence the gut where pathogen levels are relatively high [28]. In addition, the liver processes and stores fat-soluble vitamins [29]. For spine-435 cells such a pathway was the up-regulated ‘cholesterol biosynthesis, regulation, and transport’ pathway, which is important for bone growth hemostasis as well as neuronal function [30, 31]. Surprisingly, reported tumor/metastatic suppressor pathways: ‘transcriptional regulation by RUNX3’, ‘death receptor signaling’, and ‘regulation of PTEN stability and activity’ were up-regulated in lymph node-231 cells and as such deserve further investigation as to the function of these pathways in normal lymph nodes/lymphocytes/hematopoiesis, and reticular cells.

**Table 7:** Unique pathways found to be common in both proteomic and transcriptomic analyses for the metastatic brain-435 cell line.

Source	Up Pathways	# of Gs-Ps^ǂ^ in Set	# of Obs. Gs-Ps	Obs. Gs-Ps (%)	q-value
Wikipathways	MAPK Signaling Pathway	246	4	1.6	0.020124
KEGG	MAPK Signaling Pathway	295	4	1.4	0.020124
Reactome	Integrin Cell Surface Interactions	67	3	4.5	0.020124
KEGG	C-Type Lectin Receptor Signaling Pathway	104	3	2.9	0.020124
KEGG	Apelin Signaling Pathway	137	3	2.2	0.020124
KEGG	Phospholipase D Signaling Pathway	146	3	2.1	0.020124
Wikipathways	Inhibition of Exosome Biogenesis & Secretion by Manumycin A in CRPC Cells	18	2	11.1	0.020124
PID	Plexin-D1 Signaling	24	2	8.3	0.020124
Reactome	Ca-Dependent Events	28	2	7.1	0.020124
Wikipathways	p38 MAPK Signaling Pathway	34	2	5.9	0.020124
	**Down Pathways**				
PID	E2F Transcription Factor Network	75	6	8.0	1.46E-05
Reactome	M Phase	340	9	2.7	0.000163
Reactome	Removal of the Flap Intermediate	14	3	21.4	0.000182
Reactome	Polymerase Switching on the C-Strand of the Telomere	14	3	21.4	0.000182
Reactome	Polymerase Switching	14	3	21.4	0.000182
Reactome	Leading Strand Synthesis	14	3	21.4	0.000182
Reactome	Processive Synthesis on the Lagging Strand	15	3	20.0	0.000220
Reactome	Mitotic Anaphase	140	6	4.3	0.000239
Reactome	Mitotic Metaphase & Anaphase	141	6	4.3	0.000242
PID	PLK1 Signaling Events	44	4	9.1	0.000242

^**ǂ**^Gs-Ps denotes Genes-Proteins, i.e., the input dataset was a common (combined) genes-proteins dataset.

**Table 8:** Unique pathways found to be common in both proteomic and transcriptomic analyses for the metastatic liver-435 cell line.

Source	Up Pathways	# of Gs-Ps^ǂ^ in Set	# of Obs. Gs-Ps	Obs. Gs-Ps (%)	q-value
KEGG	Lysosome	123	9	7.3	2.51E-08
Reactome	Neutrophil Degranulation	490	11	2.3	2.11E-05
Reactome	Innate Immune System	1077	13	1.2	0.001011
Reactome	MHC Class II Antigen Presentation	59	4	6.8	0.001519
KEGG	Antigen Processing & Presentation	77	4	5.2	0.003464
Reactome	Hemostasis	668	9	1.3	0.006099
Reactome	Immune System	1840	15	0.8	0.008389
Reactome	Metabolism of Fat-soluble Vitamins	49	3	6.1	0.010501
Reactome	Trafficking & Processing of Endosomal TLR	13	2	15.4	0.011678
Reactome	TP53 Regulates Transcription of Several Additional Cell Death Genes Whose Specific Roles in p53-dependent Apoptosis Remain Uncertain	14	2	14.3	0.012152
	**Down Pathways**				
Reactome	Resolution of Abasic Sites (AP Sites)	37	3	8.1	0.001607
Reactome	Base Excision Repair	37	3	8.1	0.001607
Reactome	Dual Incision in GG-NER	41	3	7.3	0.001991
KEGG	Nucleotide Excision Repair (NER)	47	3	6.4	0.002742
Reactome	Apoptotic Execution Phase	52	3	5.8	0.003478
INOH	DroToll-like	65	3	4.6	0.005417
Reactome	Gap-filling DNA Repair Synthesis & Ligation in TC-NER	68	3	4.4	0.005837
Reactome	Dual incision in TC-NER	69	3	4.3	0.005970
INOH	Hedgehog	72	3	4.2	0.006618
Reactome	Transcription-Coupled Nu-NER	81	3	3.7	0.008149

^**ǂ**^Gs-Ps denotes Genes-Proteins, i.e., the input dataset was a common (combined) genes-proteins dataset.

**Table 9:** Unique pathways found to be common in both proteomic and transcriptomic analyses for the metastatic lung-435 cell line.

Source	Up Pathways	# of Gs-Ps^ǂ^ in Set	# of Obs. Gs-Ps	Obs. Gs-Ps (%)	q-value^1^
Wikipathways	miR-targeted Genes in Muscle Cell—TarBase	400	12	3.0	0.055336
KEGG	Herpes Simplex Infection	185	8	4.3	0.055336
Reactome	rRNA Modification in the Nucleus & Cytosol	59	5	8.6	0.055336
Reactome	rRNA Processing in the Nucleus & Cytosol	59	5	8.6	0.055336
Reactome	rRNA Processing	65	5	7.8	0.055336
	**Down Pathways**				
Reactome	Post-translational Protein Phosphorylation	110	8	7.3	0.000940
Reactome	Collagen Chain Trimerization	44	5	11.4	0.002614
KEGG	ECM-receptor Interaction	82	6	7.3	0.004541
Reactome	N-Glycan Antennae Elongation	15	3	20.0	0.009370
Wikipathways	Senescence & Autophagy in Cancer	106	6	5.7	0.010738
Reactome	Chylomicron Clearance	5	2	40.0	0.013331
Reactome	Sulfide Oxidation to Sulfate	5	2	40.0	0.013331
KEGG	Protein Digestion & Absorption	90	5	5.6	0.020575
Reactome	N-Glycan Antennae Elongation in the Medial/Trans-Golgi	26	3	11.5	0.020575
Reactome	Sulfur Amino Acid Metabolism	27	3	11.5	0.020575

^**ǂ**^Gs-Ps denotes Genes-Proteins, i.e., the input dataset was a common (combined) genes-proteins dataset.

^**1**^Gray shading of values indicates that the pathways are trending to significance.

**Table 10:** Unique pathways found to be common in both proteomic and transcriptomic analyses for the metastatic spine-435 cell line.

Source	Up Pathways	# of Gs-Ps^ǂ^ in Set	# of Obs. Gs-Ps	Obs. Gs-Ps (%)	q-value
Wikipathways	Cholesterol Biosynthesis, Regulation & Transport	9	3	33.3	2.64E-05
SMPDB	Simvastatin Action Pathway	22	3	13.6	2.64E-05
SMPDB	Hyper-IgD Syndrome	22	3	13.6	2.64E-05
SMPDB	Cholesteryl Ester Storage Disease	22	3	13.6	2.64E-05
SMPDB	Lysosomal Acid Lipase Deficiency (Wolman Disease)	22	3	13.6	2.64E-05
SMPDB	Mevalonic Aciduria	22	3	13.6	2.64E-05
SMPDB	Wolman Disease	22	3	13.6	2.64E-05
SMPDB	Smith-Lemli-Opitz Syndrome	22	3	13.6	2.64E-05
SMPDB	Chondrodysplasia Punctata II, X Linked Dominant (CDPX2)	22	3	13.6	2.64E-05
SMPDB	CHILD Syndrome	22	3	13.6	2.64E-05
	**Down Pathways**				
INOH	Citrate cycle	32	4	12.5	0.000135
Reactome	Dissolution of Fibrin Clot	13	3	23.1	0.000221
PID	β3-Integrin Cell Surface Interactions	44	4	9.1	0.000447
Wikipathways	Hereditary Leiomyomatosis & Renal Cell Carcinoma Pathway	20	3	15.0	0.000751
Wikipathways	Interleukin-4 & Interleukin-13 Signaling	97	5	5.2	0.000751
Reactome	Basigin Interactions	27	3	11.5	0.001521
Wikipathways	Macrophage Markers	9	2	22.2	0.004074
Wikipathways	miR-targeted Genes in Leukocytes—TarBase	154	5	3.2	0.005464
Reactome	eNOS Activation	11	2	18.2	0.005929
Wikipathways	Prostaglandin Synthesis & Regulation	44	3	6.8	0.006368

^**ǂ**^Gs-Ps denotes Genes-Proteins, i.e., the input dataset was a common (combined) genes-proteins dataset.

**Table 11:** Unique pathways found to be common in both proteomic and transcriptomic analyses for the metastatic lung-231 cell line.

Source	Up Pathways	# of Gs-Ps^ǂ^ in Set	# of Obs. Gs-Ps	Obs. Gs-Ps (%)	q-value
Reactome	Vitamin B5 Metabolism	14	4	28.6	0.010842
Reactome	RHO GTPase Effectors	301	13	4.3	0.017389
Reactome	Metabolism of Nucleotides	105	8	7.6	0.017389
KEGG	Pantothenate & CoA Biosynthesis	19	4	21.1	0.017389
SMPDB	UMP Synthase Deiciency (Orotic Aciduria)	23	4	17.4	0.017389
SMPDB	MNGIE (Mitochondrial Neurogastrointestinal Encephalopathy)	23	4	17.4	0.017389
SMPDB	β-Ureidopropionase Deficiency	23	4	17.4	0.017389
SMPDB	Dihydropyrimidinase Deficiency	23	4	17.4	0.017389
SMPDB	Mercaptopurine Action Pathway	47	5	10.6	0.019771
SMPDB	Azathioprine Action Pathway	47	5	10.6	0.019771
	**Down Pathways**				
Reactome	Golgi Associated Vesicle Biogenesis	56	9	16.1	0.001709
KEGG	Mucin Type O-glycan Biosynthesis	31	6	19.4	0.006683
PID	α6-β4-Integrin-Ligand Interactions	11	4	36.4	0.006683
KEGG	Apoptosis	136	12	8.8	0.010760
HumanCyc	Ethanol Degradation IV	6	3	50.0	0.012092
PID	p75(NTR)-Mediated Signaling	71	8	11.4	0.015730
HumanCyc	Oxidative Ethanol Degradation III	7	3	42.9	0.016350
Wikipathways	Pentose Phosphate Metabolism	7	3	42.9	0.016350
Wikipathways	VEGFA-VEGFR2 Signaling Pathway	236	16	6.8	0.016480
EHMN	Phytanic Acid Peroxisomal Oxidation	16	4	25.0	0.016570

^**ǂ**^Gs-Ps denotes Genes-Proteins, i.e., the input dataset was a common (combined) genes-proteins dataset.

**Table 12:** Unique pathways found to be common in both proteomic and transcriptomic analyses for the metastatic lymph node-231 cell line.

Source	Up Pathways	# of Gs-Ps^ǂ^ in Set	# of Obs. Gs-Ps	Obs. Gs-Ps (%)	q-value
Reactome	EPH-Ephrin Signaling	74	8	10.8	0.007998
PID	Regulation of RhoA Activity	48	6	13.0	0.016774
NetPath	EGFR1	457	20	4.4	0.017595
Wikipathways	VEGFA-VEGFR2 Signaling Pathway	236	13	5.5	0.019780
Reactome	Transcriptional Regulation by RUNX3	52	6	11.8	0.019780
Wikipathways	TGF-β Signaling Pathway	132	9	6.	0.028468
Reactome	mRNA 3-End Processing	58	6	10.3	0.028468
Reactome	Death Receptor Signaling	141	9	6.5	0.037155
PID	PAR1-mediated Thrombin Signaling Events	44	5	11.6	0.037493
Reactome	Regulation of PTEN Stability & Activity	25	4	16.0	0.037493
	**Down Pathways**				
EHMN	Dimethyl-branched-chain Fatty Acid MIT β-Oxidation	12	5	41.7	0.001962
KEGG	Fc-γ R-mediated Phagocytosis	91	12	13.3	0.002325
PID	Stabilization & Expansion of the E-cadherin Adherens Junction	42	8	19.0	0.003070
INOH	Val, Leu, & Ile Degradation	32	7	21.9	0.003113
SMPDB	MIT β-Oxidation of Short Chain Saturated Fatty Acids	8	4	50.0	0.003113
SMPDB	Short-chain 3-Hydroxyacyl-CoA Dehydrogenase Deficiency	8	4	50.0	0.003113
KEGG	Val, Leu, and Ile Degradation	48	8	16.7	0.004706
HumanCyc	Rapoport-Luebering Glycolytic Shunt	5	3	75.0	0.004706
Reactome	Mitochondrial Protein Import	65	9	13.8	0.006831
EHMN	3-Oxo-10R-Octadecatrienoate β-Oxidation	11	4	36.4	0.008750

^**ǂ**^Gs-Ps denotes Genes-Proteins, i.e., the input dataset was a common (combined) genes-proteins dataset.

#### Metabolomic-based pathway discovery

As a further means of confirming the pathways revealed by ConsensusPathDB when using the protein or transcript lists from the proteomic and RNAseq studies we submitted lists of aqueous phase metabolites that were acquired from an earlier metabolomics study of the 435 cell lines, which included hierarchical clustering and principle component analyses but no pathway analyses [12], to ConsensusPathDB. The resulting complete metabolomic pathway discovery and comparison pathways along with the metabolite CAS numbers are given in S21–S28 Spreadsheets. The top 10 up- and down-regulated pathways (q ≤ 0.05) for each of the isogenic metastatic cell lines are presented in S25–S28 Tables. For the brain-435 and liver-435 cell lines the up-regulated pathway lists were generally based on a small number of metabolites (2–4 metabolites) as defining the pathways, which, were statistical significant, but as noted above (**Common proteomic and transcriptomic pathways**) their biological relevance needs further investigation. Also, these analyses revealed a possible limitation to the CensusPathDB platform as it was found; e.g., in the up-regulated pathway list of lung-435 cells that the SMPDB Source gave 5 apparent independent pathways, but these were based on the same 6 metabolites (S25 Spreadsheet). Thus, as noted above during the proteomic-based pathway discovery analyses, online pathway designations can result in lists of similar related or identical pathways that have been given different labels, which appears to have been the case with this example of SMPDB designations. The latter was again apparent for all 4 lists of down-regulated pathways shown in S25–S28 Tables.

#### Metabolomic-based unique pathways

In these cases (S29–S32 Tables), the majority of pathways were either considered as being weakly defined (2–4 metabolites/pathway) or the different pathway designations were based on identical metabolite lists; e.g., Source SMPDB in the down-regulated list for the liver-435 cell line (S30 Table and S24 Spreadsheet) and in the up- and down-regulated lists of lung-435 (S31 Table and S25 Spreadsheet).

#### Common metabolomic and proteomic pathways

An analysis of these common pathways revealed only two (e.g., in brain-435, lung-435, and spine-435) or no (liver-435) up-regulated pathways but those that were found were unique to each cell line (S33–S36 Tables). In addition, the two up-regulated transport pathways (S36 Table) of spine-435 may indicate some specific influence of the neuronal component of spine during growth in the spine. On the other hand, most of the top 15 common down-regulated pathways (S33–S36 Tables) were shared between cell lines. For example, the ‘cell cycle, mitotic’ (Source: Reactome) pathway was down in brain-, liver-, and spine-435 cell lines while the ‘pyrimidine metabolism” (Source: KEGG) pathway was down in brain-, liver-, and lung-435 cell lines. Those pathways that were down-regulated in brain-435 and liver-435 cell lines included: ‘cell cycle’, ‘translation’, as well as ‘S phase’ (Source: Reactome) along with the ‘pyrimidine metabolism’ (Source: Wikipathways) pathway, and ‘purine metabolism’ (Source: KEGG) pathway. The ‘post-translational protein modification’ (Source: Reactome) pathway was shared by brian-435 and lung-435 while the ‘metabolism of nucleotides’ (Source: Reactome) pathway was shared by brain-435 and spine-435. The vaguely defined ‘metabolism’ (Source: Reactome), ‘pyrimidine nucleotides nucleosides metabolism (Source: INOH) along with ‘DNA replication’ and ‘selenoamino acid metabolism’ (Source: Reactome) pathways were unique to brain-435. This type of pattern of shared pathways can be seen when S33–S36 Tables were compared. Unique pathways for liver-435 (S34 Table) were: the ‘superpathway of purine nucleotides salvage’ (Source: HumanCyc) pathway along with ‘DNA replication’, ‘teleomere C-strand (lagging strand) synthesis’, and ‘TCA cycle and respiratory electron transport’ (Source: Reactome) pathways. For the lung-435 cell line (S35 Table) the unique pathways were the: ‘pentose phosphate pathway’, ‘glycogenosis, type IA, von Gierke Disease’, and ‘pyrimidine metabolism’ (Source: SMPDB) pathways, and ‘interconversion of nucleotide di- and triphosphate’ and ‘asn N-linked glycosylation’ (Source: Reactome) pathways as well as the ‘glucagon signaling pathway’ (Source: KEGG). The unique down-regulated pathways for spine-435 were: ‘gluconeogenesis’, Fanconi-Bickel syndrome’, and ‘oncogenic action of succinate’ (Source: SMPDB) pathways, along with ‘citrate cycle’ and ‘aminosugars metabolism’ (Source: INOH) pathways, and the ‘pyruvate metabolism and TCA cycle’ (Source: Reactome) pathway.

#### Common metabolomic and transcriptomic pathways

These pathways are presented in S37–S40 Tables. Similar to S33–S36 Tables, no, or only one or two common up-regulated pathways were found. Of these the single up-regulated pathway: ‘arg and pro metabolism’ (Source: INOH) found for the brain-435 cell line was shared with the spine-435 cell line while the ‘metabolism of amino acids and derivatives’ (Source: Reactome) and the ‘his, lys, phe, tyr, pro, and trp catabolism’ (Source: Reactome) pathways were uniquely up-regulated in lung-435 and spine-435 cell lines respectively. Again, similar to what was found in S33–S36 Tables, several of the top 12–15 down-regulated pathways were shared between cell lines but more of the pathways were unique to each cell line. Thus, for the brain-435 cell line the unique down-regulated pathways were: ‘pyrimidine metabolism’ (Source: Wikipathways) along with ‘chromosome maintenance’, ‘telomere maintenance’, ‘extension of telomeres’, and ‘synthesis of DNA’ (Source: Reactome) pathways; for liver-435 cells the unique pathways were; ‘pyrimidine metabolism’, ‘pentose phosphate pathway’, urea cycle and metabolism of arg, pro, glu, asp, and asn’, and ‘purine metabolism’ (Source: EHMN) along with ‘interconversion of nucleotide di- and triphosphates’, ‘DNA replication initiation’, and ‘transcriptional regulation by TP53’ (Source: Reactome) and ‘pyrimidine metabolism’ (Source: KEGG); for the lung-435 cell line the unique pathways were: ‘metabolism of carbohydrates’ (Source: Reactome) and ‘glycolysis gluconeogenesis’ (Source: INOH); finally, the unique spine-435 cell line pathways were: ‘gluconeogenesis’, ‘gluconeogenesis, type IA, von Gierke disease’, ‘glycolysis’, and ‘Fanconi-Bickel syndrome’ (Source: SMPDB) along with ‘superpathway of conversion of glucose to acetyl CoA and entry into the TCA cycle’ and ‘gluconeogenesis’ (Source: HumanCyc), ‘metabolite reprogramming in colon cancer’ and ‘Cori cycle’ (Source: Wikipathways), ‘glucose metabolism’, gluconeogenesis’, and ‘glycolysis’ (Source: Reactome).

Importantly, a comparison of S33–S36 Tables with corresponding S37–S40 Tables indicated many of the pathways in these sets of tables; e.g., S33 Table vs S37 Table, *etc*., were the same. Consequently, we found identical pathways, albeit with all but one being down-regulated pathways, that had been discovered by the use of three separate data sets, i.e., proteomic, transcriptomic, and metabolomic. Thus, a comparison of these tables showed a convergence of the three data sets onto common pathways. For the brain-435 cell line these were down-regulated: ‘cell cycle’, ‘cell cycle, mitotic’, ‘pyrimidine metabolism’ (Source: Wikipathways), ‘S phase’, ‘DNA replication’, and ‘extension of telomeres’; for the liver-435 cell line these were down-regulated: ‘pyrimidine metabolism’ (Source: EHMN), ‘nucleotide di- and triphosphates’, ‘S phase’, ‘DNA replication’, ‘pyrimidine metabolism’ (Source: KEGG), ‘telomere C-strand (lagging strand) synthesis’, ‘cell cycle’, and ‘cell cycle, mitotic’; for the lung-435 cell line these were the up-regulated: ‘metabolism of amino acids and derivatives’, and the down-regulated: ‘metabolism of carbohydrates’ and, for the spine-435 cell these were the down-regulated: ‘gluconeogenesis’ (Source: SMPDB), ‘superpathway of conversion of glucose to acetyl CoA and entry into the TCA cycle’, ‘metabolic reprogramming in colon cancer’, ‘Fanconi-Bickel syndrome’, ‘glucose metabolism’ and ‘Cori cycle’.

### qRT-PCR verification

**Fig 8:**
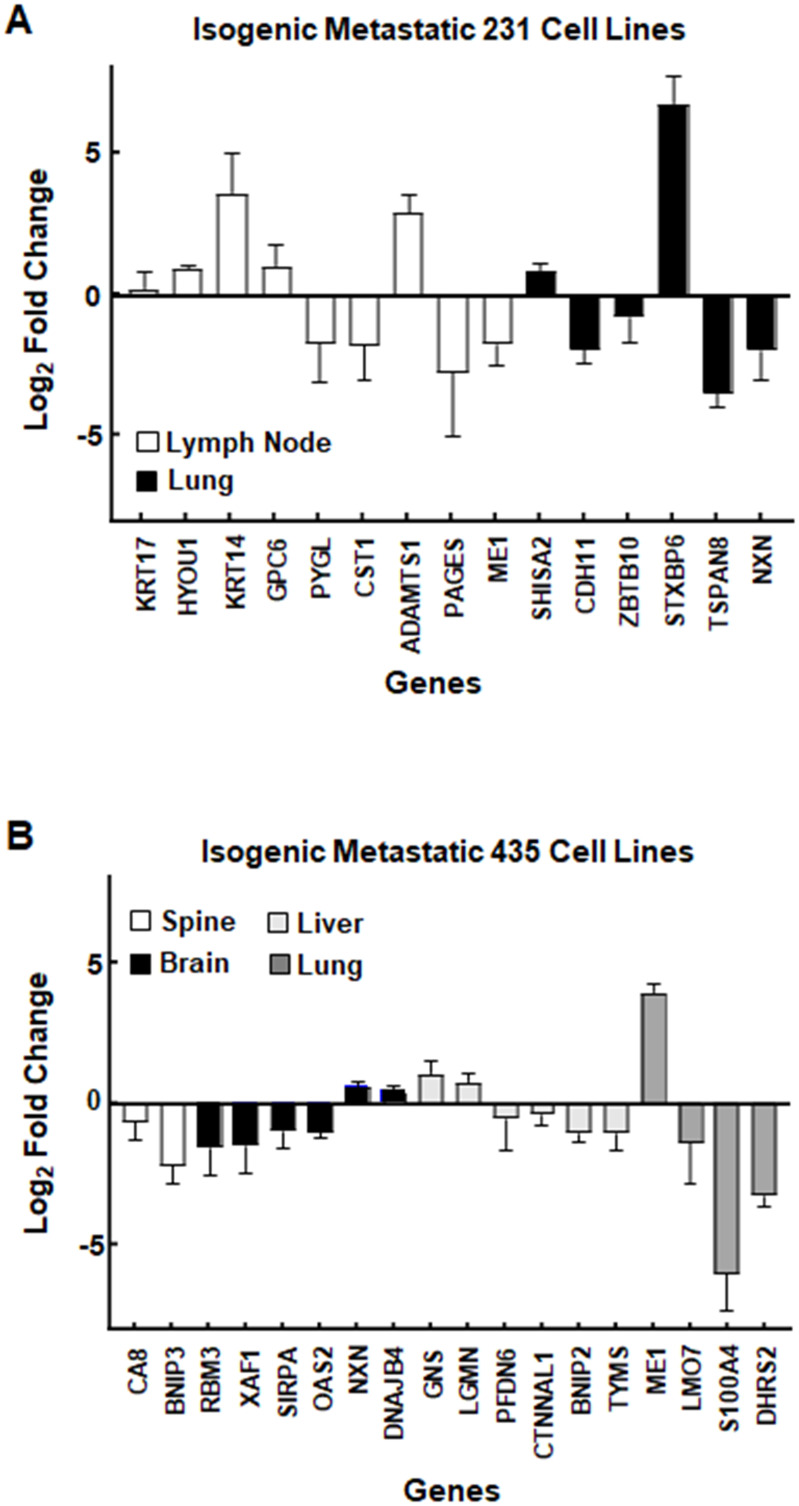
Bar plot presentations of quantitative real-time PCR (qRT-PCR) results. (A) qRT-PCR results for isogenic metastatic 231 cell lines (lung: black bars and lymph node: white bars). (B) qRT-PCR results for isogenic metastatic 435 cell lines (brain: black bars, liver: light gray bars, lung: dark gray bars, and spine: white bars). The genes (x-axis) are plotted against their log_2_ fold changes (y-axis).

It has been noted above that proteomic derived protein lists could only be sparingly matched to their corresponding transcripts from the RNAseq analyses. Our qRT-PCR results were similar in that only a small percentage of the protein and transcript levels found during the proteomics and RNAseq analyses could be confirmed with qRT-PCR. Representative genes analyzed by qRT-PCR are given in Table 7 along with their fold changes in their respective protein and transcript (gene) lists. Bar graphs of the qRT-PCR results are presented in Fig 8A and 8B for the isogenic metastatic 231 and 435 cell lines respectively. In all cases, the direction (up- or down-regulated) matched the up- and down-regulation of their proteins and transcripts (Table 13).

**Table 13:** Genes common to proteomic and transcriptomic data sets and their linear fold change (FC) relative to their 1° tumors were verified with qRT-PCR.

				Linear FC	
Cell line	Gene ID	Symbol	Description	Protein	Gene
**Brain-435**	11080	DNAJB4	Heat Shock Protein 40 Homolog4	1.40	1.25
	64359	NXN	Nucleoredoxin	1.25	1.40
	5935	RBM3	RNA Binding Motif Protein 3	-3.90	-1.40
	4939	OAS2	2’-5’-Oligoadenylate Synthetase 2	-2.35	-1.25
	54739	XAF1	X-Linked Inhibitor of Apoptosis Associated Factor 1	-2.25	-1.35
	140885	SIRPA	Signal-Regulatory Protein-α	-1.45	-1.30
**Liver-435**	5641	LGMN	Legumain	1.30	1.60
	2799	GNS	Glucosamine N-Acetyl-6-Sulfatase	1.25	1.85
	10471	PFDN6	Prefoldin Subunit 6	-1.90	-1.40
	8727	CTNNAL1	Catenin-α Like-1	-1.60	-1.25
	663	BNIP2	BCL2 Interacting Protein 2	-1.50	-1.35
	7298	TYMS	Thymidylate Synthetase	-1.50	-1.35
**Lung-435**	4199	ME1	Malic Enzyme 1	4.90	77.50
	6275	S100A4	S100 Calcium Binding Protein A4	-11.25	-26.15
	4008	LMO7	LIM Domain 7	-3.10	-3.80
	10202	DHRS2	Dehydrogenase/Reductase 2	-2.00	-3.80
**Spine-435**	767	CA8	Carbonic Anhydrase 8	1.25	1.29
	664	BNIP3	BCL2 Interacting Protein 3	-1.55	-2.50
**Lung-231**	29091	STXBP6	Syntaxin Binding Protein 6	4.50	36.60
	387914	SHISA2	Shisa Family Member 2	1.30	1.80
	7103	TSPAN8	Tetraspanin 8	-40.00	-10.30
	64359	NXN	Nucleoredoxin	-2.50	-2.10
	1009	CDH11	Cadherin 11	-2.10	-2.65
	65986	ZBTB10	Zinc Finger & BTB Domain Containing 10	-1.35	-1.50
**L.N.-231**	3872	KRT17	Keratin 17	9.85	1.50
	9510	ADAMTS1	ADAM Metallopeptidase w/Thrombospondin Type-1 Motif-1	6.10	11.20
	3861	KRT14	Keratin 14	3.00	10.20
	10525	HYOU1	Hypoxia Up-Regulated 1	2.30	1.75
	10082	GPC6	Glypican 6	1.80	3.10
	90737	PAGE5	PAGE Family Member 5	-5.05	-14.20
	4199	ME1	Malic Enzyme 1	-4.25	-2.10
	1469	CST1	Cystatin SN	-2.45	-1.58
	5836	PYGL	Glycogen Phosphorylase L	-2.06	-1.71

### *In vitro* drug testing

From our earlier studies [12, 13] and reports from other labs [5, 6, 8, 10, 11, 32], it is becoming established that metastatic lesions have diverged from their primary tumors at several levels: genetically, proteomically, and metabolically. Thus, we have shown here that isogenic cell lines derived from different organs have distinct divergences between their proteomes, transcriptomes, and metabolomes that make each of them fundamentally different biological entities. Given this, an important question arises about how cancer cells that have adapted to growth in different organs respond to the same therapeutic regimes. To ascertain whether the same drug will kill each cell line with the same efficacy, we tested 4 drugs on each of the isogenic cell lines: one RK-33 (DDX3X inhibitor) [33–35] along with three FDA approved and breast treatment established (doxorubicin (DOX) [36], gemcitabine (GEM) [37], and paclitaxel (PAC) [38] drugs, using an *in vitro* cell culture assay. Table 14 indicates that all of the drugs exhibited a range of efficacies (IC_50_ values) across cell lines. From the perspective of therapy, we were interested in knowing what significant differences (2-sided Student’s *t*-test *p* ≤ 0.001 or as indicated with *p* ≤ 0.05 considered significant) there were between metastatic cell lines and, primarily, between the metastatic cell lines and their 1° tumor cell lines. Significantly different IC_50_ values were for: RK-33 in the case of liver-435 having an IC_50_ lower-than the IC_50_ values of lung-453 (*p* = 0.028) and trending to significance in comparison to 1° tumor-435 (*p* = 0.053); GEM in the case of lung-231 with an IC_50_ higher-than those of lymph node-231, 1° tumor-231, brain-435, liver-435, lung-435, spine-435, and 1° tumor-435; PAC in the case of lung-435 with an IC_50_ higher-than that of lung-231 (*p* = 0.01); GEM in the case of spine-435 with an IC_50_ value lower-than lymph node-231 (*p* = 0.033) and brain-435 (*p* = 0.005); DOX in the case of spine-435 with an IC_50_ value higher-than those of lung-231, lymph node-231, 1° tumor-231 (*p* = 0.005), brain-435 (*p* = 0.008), liver-435 (*p* = 0.015), lung-435 (*p* = 0.003), and 1° tumor-435 (*p* = 0.027); DOX in the case of liver-435 with an IC_50_ value higher-than those of lung-231 and lymph node-231 (*p* = 0.005) and trending to significance in comparison to 1° tumor-435 (*p* = 0.056); DOX in the case of brain-435 with an IC_50_ value higher-than those of lung-231 (*p* = 0.009), lymph node-231, and 1° tumor-435; DOX in the case of lung-435 with an IC_50_ value greater-than 1° tumor-435. In addition, we determined the linear fold change in any differences in efficacy between the 1° tumors and their isogenic cell lines, which are shown in Table 15 where the values in bold-type and underlined are statistically significant (2-sided Student’s *t*-test *p* < 0.001 or as indicated with *p* ≤ 0.05 considered significant).

**Table 14:** Mean IC_50_ values for the drugs tested against each isogenic cell line.

Cell Line	RK-33 (nM)	GEM (nM)	PAC (nM)	DOX (nM)
	Mean ± SEM^1^	Mean ± SEM	Mean ± SEM	Mean ± SEM
Parental-231	2.5 ± 0.2	69 ± 22	0.74 ± 0.04	69 ± 1
1° Tumor-231^1^	2.6 ± 0.1	32 ± 2	9.8 ± 1.9	111 ± 10
Lung-231	2.7 ± 0.7	247 ± 9	3.6 ± 0.3	65 ± 3
Lymph Node-231	2.3 ± 0.7	16 ± 1	2.9 ± 0.4	44 ± 1
Parental-435	5.6 ± 0.1	13 ± 1	0.6 ± 0.2	60 ± 4
1° Tumor-435	3.1 ± 0.1	16 ± 1	1.7 ± 0.1	78 ± 0.5
Brain-435	4.2 ± 0.3	20 ± 0.4	2.0 ± 0.1	220 ± 0.7
Liver-435	1.9 ± 0.0	11 ± 4	2.4 ± 0.3	214 ± 9
Lung-435	3.1 ± 0.1	8.4 ± 2	7.3 ± 1.2	147 ± 1
Spine-435	5.5 ± 0.7	4 ± 1	1.8 ± 0.1	347 ± 8

^**1**^Abbreviations: SEM denotes standard error of the mean and 1° Tumor denotes primary tumor.

**Table 15:** Summary of linear fold change of IC_50_ values for metastatic isogenic cell lines relative to their 1° tumors.

Cell Line	RK-33	GEM	PAC	DOX
	**Fold Change vs 1**^**°**^ **Tumor-231**			
**Lung-231**	------	7.7*	-2.7	-1.7
**Lymph Node-231**	------	**-2.6** **(0.037)**	-3.3	-2.5
	**Fold Change vs 1**^**°**^ **Tumor-435**			
**Brain-435**	1.4	1.25	1.2	**2.8**
**Liver-435**	-1.6 (0.053)	-1.5	1.4	2.7 (0.056)
**Lung-435**	------	-1.9	4.3	**1.9**
**Spine-435**	**1.8** **(0.042)**	**-4.0** **(0.023)**	------	**4.4** **(0.027)**

*Bold-type that is underlined indicates that these changes are statistically signifi-cant, i.e., 2-sided Student’s *t*-test: *p* < 0.001 or at *p*-values given in parenthesis. Gray shading indicates a trending towards significance.

## Discussion

Presently, despite advances in therapies, metastatic breast cancer remains incurable [1, 39]. To address the reasons as to why this is the case, several independent laboratories have provided evidence, over the course of several decades that metastatic tumors have, to varying degrees, diverged from their primary tumors [3, 4, 6–8, 11, 32]. Consequently, it is now realized that therapies that are effective against regional breast cancer, which are based on a few molecular markers that have been used to define breast cancer subtypes, can have minimal efficacy against metastases that have diverged from the primary tumor [4, 5, 7, 11]. Accordingly, with the advent of more recent molecular (“omics”) characterizations, it is becoming accepted that careful clinical evaluations of the molecular (proteome and transcriptome) as well as metabolism of metastatic lesions would be helpful and likely necessary if efficacious treatment of metastatic disease is to be developed [9, 11].

As described in previous reports [12, 13] and extended here, our approach to this problem of gaining a better understanding of the molecular and metabolic changes that occur at metastatic lesions relative to their primary tumors, has been the development and characterization of model systems of isogenic cell lines that have been cultured directly from metastatic organ samples. The advantage of this model system with respect to those that rely on several cell lines from different individuals is the isogenic nature of the cell lines. From this perspective, phenotypic, molecular, and metabolic divergences from an isogenic primary tumor, as is the case in clinical settings, can be assessed within the context of the isogenic background of these cell lines. As described above and discussed below, we have found that these cell lines represent unique biological entities that have diverged from their primary tumors in growth characteristics in culture, proteomics, transcriptomics, as well as metabolomics. A principle goal has been to study the sensitivity of these different cell lines to chemotherapeutics as well as in future *in vivo* studies.

Here we tested a DDX3X (DEAD box helicase) inhibitor (RK-33) [33–35] and three clinically established breast cancer chemotherapeutics: gemcitabine (GEM) [37], paclitaxel (PAC) [38], and doxorubicin (DOX) [36]. As shown in Table 14, we found several differences in efficacy across drug treatments of the cell lines. For example, relative to all other cell lines, the lung-231 cell line was the least sensitive to GEM and exhibited a critical ~8-fold decrease in sensitivity as compared to its primary tumor cell line. On the other hand, lung-435 and spine-435 were the most sensitive to GEM and again differed (being more sensitive) relative to their primary tumor cell lines. In addition, lung-231 and lymph node-231 cell lines were more sensitive than their primary tumor cell line to DOX but the reverse was the case for the four metastatic 435 cell lines, which were less sensitive to DOX than their primary tumor cell line. Other distinctions were the lung-435 cell line being less sensitive to PAC than was the case for its primary tumor cell line. Although significant differences were observed in the sensitivity to RK-33 the changes were relatively less pronounced, and this latter characteristic of RK-33 may be a therapeutic advantage. That is, as there is a molecular dependency for DDX3X expression in cancer cells to maintain cellular and bioenergetic homeostasis [34, 40, 41], it is less likely to undergo marked changes during growth and establishment of metastatic tumors. This, in part, could explain why RK-33 doses required to kill the different isogeneic cell lines was the least variable, particularly evident in the 231 cell lines (Table 14), amongst the different chemotherapeutic agents used in this study.

To attempt an explanation for the observed differences in drug efficacies it needs to be noted that the cellular mechanisms involved with GEM, PAC, and DOX are complex and multiple pathways and several proteins must be taken into consideration [36–38]. This problem is exemplified from an evaluation of changes in some of the single proteins that might be involved with the decreased efficacy of GEM against lung-231 cells [37]. Thus, there was a 1.7-fold increase in a protein inhibited by GEM (ribonucleotide reductase 1: RRM1) in lung-231 cells, which might be evaluated as a requirement of more drug against this target, i.e., a decrease in sensitivity. However, there was a simultaneous 1.8-fold increase in a solute carrier (SLC29A1) that transports GEM into cells as well as decreases (-1.9-fold in each case) in inactivating enzymes (cytidine deaminase and cytosolic 5’ nucleotidase). Consequently, in this case, a simple consideration of proteins involved with GEM metabolism in these cells provides an ambiguous conclusion as to the lack of sensitivity of lung-231 cells to GEM treatment. Contributing to this ambiguity is that in cell lines more sensitive to GEM, such as brain-435 and liver-435 (Table 14), SLC29A1 was found to be decreased by -1.8 and -1.35 respectively, which when coupled with decreases in activating enzymes: deoxycytidine kinase (-1.30- and -1.67-fold in brain-435 and liver-435 respectively), UMP/CMP kinase (-1.25- and -1.35-fold in brain-435 and liver-435 respectively) and nucleoside-diphosphate kinase (-1.50-fold in both cell lines) would tend to lead to the conclusion that GEM ought to relatively ineffective in these cell lines rather than relatively more effective. Similar decreases in activating enzymes were found in the lung-435 and spine-435 cell lines and yet these were the most sensitive to GEM. A similar evaluation of DOX’s efficacy in these cell lines also produced similar findings. Thus, a consideration of 18 proteins [36] involved with the transport, export, ability to detox reactive oxidative species (ROS), or repair DNA indicated that in most cases there was minimal or no changes in the levels of these proteins across all cell lines. Exceptions were about a 1.7-fold increase in both a DOX exporting protein (ABCB1) and a DNA repair enzyme (MSH2) in lymph node-231 cells, which were the most sensitive to DOX treatment, i.e., there was not a diminished sensitivity to DOX as compared to any of 435 metastatic cell lines. Importantly, the latter cell lines showed mostly no changes in any of the evaluated proteins except for an increase (~1.35-fold) in two enzymes (SOD1 and CAT) involved with a response to ROS in brain-435 cells and thus, again, little evidence at the protein level for the observed differences in sensitivity to DOX. Finally, exploring changes in tubulins (the major target of PAC) [38] in all cell lines showed no differences that could provide a clue as to the observed differences in sensitivity to PAC. Thus, it appears that differences other than those at the single protein levels, such as at the pathway or pathway network levels, i.e., a combination of proteins and pathways that differ between cell lines brings about the variances in the observed drug sensitivities across these cell lines.

Along these lines, our drug assay results are in line with a recent report that demonstrated a link between differences in protein networks across 41 breast cancer cell lines and changes in the sensitivity of these cell lines to drug treatments [42]. However, such a result may have been expected as the cell lines used were derived from separate individuals as well as being from across all subtypes of breast cancer. Thus, it is well known that each subtype is susceptible to different therapeutics [43] and certainly different individuals often have different responses to chemotherapies, which is always recorded in clinical trial generated patient survival curves. Accordingly, as pointed out above, this was also apparent in a comparison of our isogenic cell lines (triple negative subtype) with a reversal in sensitivity to DOX when the 231 isogenic metastatic cell lines are compared to the 435 derived isogenic metastatic cell lines. More importantly, the report of the efficacy of drugs across 41 cell lines did not take into account, as we have, how metastatic spread to visceral organs likely alters the efficacy of clinical chemotherapies and the authors did not address the treatment of metastatic disease. However, findings of molecular discordances between primary tumors and their metastasis continues to provide evidence that selecting therapeutic regimes that have been based on a characterization of the primary tumor but are aimed at ablating visceral metastatic lesions will likely be ineffective [11]. It is becoming evident that treatment strategies for metastatic disease will likely be more effective if these are based on the fundamental genetic and molecular characteristics of the metastatic lesions. The latter conclusion has been put forth in a recent clinical breast cancer study (reported while our study was in progress) that described an evaluation of evolution-based mutational changes at metastatic sites that occurred independently from any primary tumor clonal evolution and as such it was suggested that organ-specific microenvironments were driving such changes [11]. Given this evidence the authors suggested that clinical characterization of metastatic lesions ought to be carried out prior to treatment determinations of metastatic cancer [11]. Thus, our hypothesis has been strongly supported by this clinical study. However, in the present study, we have collected transcriptomic, proteomic, and metabolomic data sets and focused our analyses on pathways and their networks, which is a distinct difference from gene/protein mutational analyses that have defined the cited clinical study.

Although mutational evolutionary analyses are providing important insights into the clonal (gene-based) divergences associated with tissue dependent metastatic adaptation(s) (evolution) [9, 11, 44–46], as previously reported and expanded upon here, our isogenic model system has revealed isogenic cell line specific pathways that indeed have been influenced by the microenvironment of the cell line’s organ of origin. Examples have been pointed out in the Results section (Tables 1–6). Thus, this model provides complementary evidence (relative to the clonal evolutionary evidence) that fundamental molecular and metabolic divergences of metastatic tumors from their primary tumor are an unavoidable consequence of growth within a tissue specific microenvironment that differs vastly from that of the breast epithelial microenvironment. Another finding from the pathway analyses approach has indicated that pathways don’t necessarily fit a simple binary on/off (up/down) model but instead are likely in a state of homeostasis or steady state of regulation with pathway defining proteins being both up- and down-regulated relative to the primary tumor (S41–S46 Tables). The therapeutic implications of this finding is exemplified by the EGFR1 pathway, which has been considered as a clinical therapeutic target in breast cancer [47] but was found here to not necessarily be in a overexpressed “on” mode across 5 of 6 of the isogenic cell lines studied as this pathway also exhibited down-regulated “off” protein components across all 6 isogenic cell lines (S41–S46 Tables; The down-regulated EGFR1 pathway of lung-231 was not included as these tables show only those pathways found to be simultaneously up- and down-regulated.). In fact, EGFR1 protein was not found to be up- or down-regulated in any of these cell lines with the pathway being defined by several of the other 457 protein members of the pathway, such as up-regulated ASAP1 in liver-435, spine-435, and lymph node-231 cell lines as well as PRKCZ in brain-435, lung-435, spine-435, and lymph node-231 cell lines or the down-regulated APPL2 in lung-435, spine-435, lung-231, and lymph node-231 cell lines as well as ENO1 in all six cell lines. Moreover, there were roughly twice as many down-regulated proteins relative to the up-regulated proteins of this EGFR1 pathway (S41–S46 Tables), which indicates that the identification of a single up-regulated (overexpressed) target of such a complex pathway and using a therapeutic against it may have minimal impact on the pathway, i.e., on treatment. In addition, as exemplified in Figs 4–7, several different pathways can be interconnected into large integrated networks that are likely all regulating each other. Consequently, it is apparent that targeting a single component (protein) in what might be thought of as a single ‘key’ pathway may be ineffective due to the self-regulation of the pathway or the overlapping function of the interacting network(s). These results provide a partial explanation as to why our analysis of the single proteins involved with the effectiveness of GEM. PAC, or DOX did not show a clear association to the sensitivity of the cell lines to these drugs, i.e., their efficacies are likely based on complex pathway dynamics rather than any single protein.

**Fig 9:**
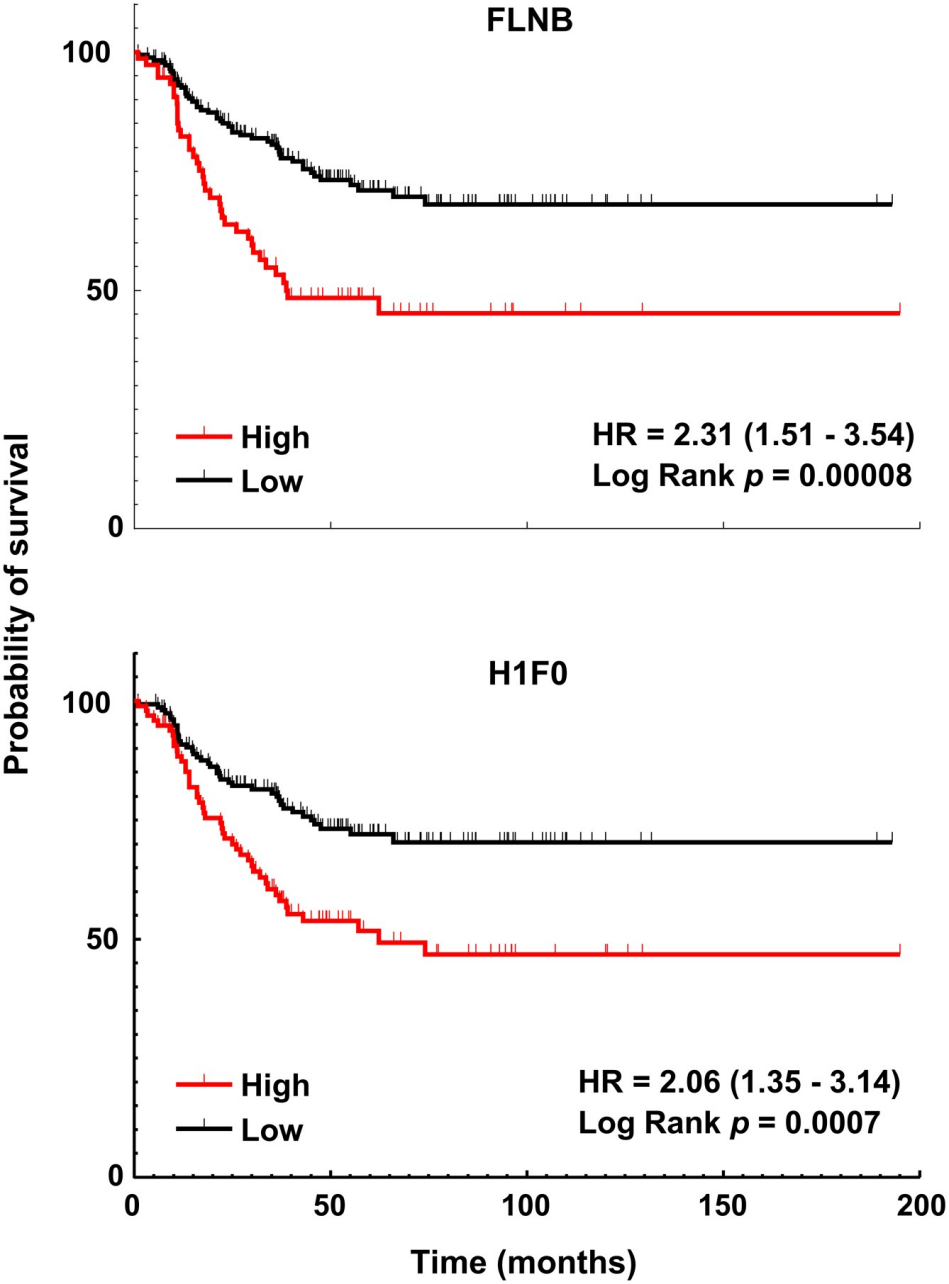
Representative survival plots of triple negative breast cancer patient data (n = 255). Genes were derived from proteomic-based up regulated pathways (Table 16) that correlated with TNBC patient relapse-free survival (RFS) datasets (Reference: PMID: 20020197). Hazard ratios indicated that high expression (red) of both FLNB and H1F0 significantly correlated with poor RFS.

Finally, it was important to analyze the possible clinical associations that our pathway approach achieved. This was hindered by the fact that available comparative human breast cancer databases (e.g., [48]) report survival as a function of an overexpression (relative to normal tissue) of markers/genes associated with the primary tumor while our studies have been focused on metastases. Nevertheless, we crossed referenced proteins from pathways that were found to be up-regulated (relative to the primary tumors) across multiple isogenic cell lines. Thus, Table 16 shows a randomly selected list of up-regulated pathways and hence proteins found to be common across 2–5 cell lines. It is noteworthy from the clinical data, i.e., elevated expression of these genes at the primary tumor, was associated with both a poor survival (e.g., Fig 9: FLNB [49] and H1F0 [50] genes) as well as an enhanced or better survival (e.g., CDC42 & HLA-A, lower portion of Table 16). Thus, this analysis indicates that an overexpression of proteins at metastatic sites, relative to primary tumor levels as well as, from the clinical data, relative to normal breast tissue levels rather than normal tissue of origin levels can complicate/contradict the interpretation of disease free survival. That is, the latter implies no metastatic progression and as such the basis of the disease free survival data does not reflect the status of the markers at metastatic sites. Consequently, this lends support to the conclusion that further studies are required to better understand how analyses of pathways at metastatic sites can contribute to a better understanding of the pathology of the metastases as well as of therapeutic options that may enhance survival.

**Table 16:** Proteomic-based up-regulated pathway proteins correlated to human patient survival.

	Cell Lines^1^ that Share the Pathway	Pathway	Gene/Protein ID	Hazard Ratio	Log Rank *p*-value^2^
**Inferior RFS**^1^	Lung-435 & Lung-231	Viral Carcinogenesis	HNRNPK	1.63 (0.96–2.77)	**0.068**
	Br-435, Li-435, Lu-435, Sp-435, & LN-231	EGFR1	FLNB	2.31 (1.51–3.54)	0.00008
	Br-435, Lu-231, & LN-231	Cellular Response to Stress	H1F0	2.06 (1.35–3.14)	0.0007
		Processing of Capped Intron-containing Pre-mRNA	ALYREF	2.40 (1.32–4.38)	0.003
			HNRNPD	1.58 (1.03–2.43)	0.036
			CWC27	1.90 (1.01–3.55)	0.042
			SNRPF	1.55 (0.99–2.44)	0.054
			SNRNP27	1.58 (0.98–2.53)	**0.057**
	Brain-435 & Lung-435	TCA Cycle	NDUFA11	1.90 (1.09–3.31)	0.021
		MIT Protein Transport	PCCB	1.61 (1.05–2.48)	0.027
			TOMM22	1.73 (0.97–3.09)	**0.062**
**Superior RFS**	Lung-435 & Lung-231	Viral Carcinogenesis	CDC42	0.56 (0.36–0.87)	0.009
			HLA-A	0.35 (0.23–0.54)	6.0E-07
			NFΚB2	0.62 (0.41–0.95)	0.027
		Metabolism of Amino Acids & Derivatives	ALDH7A1	0.61 (0.39–0.95)	0.026
	Brain-435 & Lung-435	MIT Protein Transport	PCCA	0.58 (0.36–0.93)	0.022
		Val, Leu, & Ile Metabolism	ALDH7A1	0.61 (0.39–0.95)	0.026

^**1**^Abbreviatons: RFS denotes relapse-free survival. Br-, Li-, Lu-, Sp-435, and Lu-231 & LN-231 denote Brain-435, Liver-435, Lung-435, Spine-435 and Lung-231 & Lymph Node-231 respectively.

^**2**^*p*-values in bold-type indicate that the data have been considered as trending to significance.

## Conclusions

The insights provided by these analyses indicate that the rationale of targeted treatment of metastatic disease may benefit from a consideration that the biology of metastases has diverged from the primary tumor biology and using primary tumor traits as the basis for treatment may not be ideal to design treatment strategies. Thus, exploring an interconnected integrated pathway analysis approach as an alternative to the single gene/protein marker evaluations now in use may provide a better understanding of which pathways are participating in metastatic cancer survival at a specific site. In addition, compiling normal expression levels of markers/pathways specific to different tissues would greatly aid with the discovery of changes in these levels in the metastatic lesions and pave the way for explorations as to how these changes affect treatment outcomes as well as direct future studies aimed at controlling and ablating metastatic disease.

## Materials and methods

### Generation of isogenic metastatic cell lines from specific organs

The human breast cancer cell lines: MDA-MB-435 and MDA-MB-231, were obtained from ATCC. The MDA-MB-435 cell line was established in 1976 from a pleural effusion from an untreated 31-year-old female diagnosed with adenocarcinoma of the breast [51, 52]. The MDA-MB-231 cell line was established in 1973 from a pleural effusion from an oophorectomized/chemotherapy treated 51-year-old female diagnosed with a poorly differentiated intraductal carcinoma of the breast [19]. Both cell lines were authenticated at the Johns Hopkins Genetic Resource Core Facility with the short tandem repeat marker results cross-checked against cell lines at the ATCC bank. Generation and characterization of the parental MDA-MB-435-tdTomato (hence referred to as 435) cell line has been previously described [53]. MDA-MB-231 (hence referred to as 231) cells were not genetically modified and thus were the parental cell line for this line’s isogeneic primary tumor and metastatic (lung & lymph node) cell lines. Primary tumors and subsequent tissue specific isogeneic cell lines were generated/cultured as previously described [12]. For the 231 cell lines, generation of growth curves and growth rate analysis was as previously described for the 435 cell lines [12]. All culturing was done in standard humidified incubators at 37° C and 5% CO_2_. Media were: DMEM-10% FBS for parental cell lines and DMEM:F12 (50:50)-5% FBS for all primary tumor and metastatic cell lines.

### Optical microscopy

Phase contrast microscopy was done on a Nikon ECLIPSE TS 100 microscope (Nikon Instruments, Inc.) equipped with a Photometrics CoolSnap ES digital camera (Roper Scientific). Images were collected with NIS-Elements F3.2 software and processed with ImageJ.

### Protein preparation

Total protein solutions were prepared by directly lysing cells cultured on 100 mm dishes, which, in all cases, were at about 70–80% confluency. Lysis buffer (200 μl) was: 100 mM Tris pH 6.8, 12% glycerol, and 2% SDS, 1 mM EDTA, and 1:200 dilution of a protease cocktail (Sigma, I1386) (added immediately prior to use). Lysates were placed in 0.5 ml microcentrifuge tubes and sonicated (12–15 bursts) on ice and frozen at -80° C until use.

### Protein concentration estimates

Aliquots (100 μl) from the frozen stocks (thawed on ice) of total protein preparations were placed into 0.5 ml microcentrifuge tubes. Protein concentration estimates were carried out using room-temperature samples diluted (1:10–1:15) in a phosphate-free saline solution (NaHCO_3_ (45 mM), NaCl (95 mM), KCl (4.5 mM), CaCl_2_ (0.24 mM), MgCl_2_ (0.08 mM) pH 7.35). The diluted protein solutions were assayed using a BioRad RC DC kit according the manufacturer’s protocol and BSA for the standard curve. This kit was chosen as the protein precipitate formed during step-1 of the protocol is free of compounds that interfere with the step-2 color reagent, such as EDTA, amino acids, lipids, and nucleic acids.

### Proteomics

Protein pellets (100 μg each) were submitted to the Mass Spectroscopy and Proteomics Facility at Johns Hopkins University Medical School for routine differential proteomics analyses. The Director: Dr. Robert Cole, oversaw all analyses. State-of-the-art TMTs (tandem mass tags) were applied to digested samples for direct comparison of all 10 samples in a single tandem MS experiment. The mass spectroscopy spectra output was analyzed with Proteome-Discover for peptide identification and, as such, mapped to specific protein identifiers and quantified. Data was further processed to identify fold changes in protein expression levels from isogenic metastatic cell lines relative to their primary tumor cell lines. Briefly, for each sample, the multiple spectra values for each peptide were summed to single values per unique peptide and then the many different peptide values normalized across all the samples to minimize possible technical variation. These quantile normalized log2 values were compared to determine differential peptide expression levels. In addition, all peptides were mapped to their cognate genes, which facilitated annotation and possible downstream functional analyses.

### RNAseq

RNA was prepared from frozen cell pellets (-80° C stored). The frozen stocks were from lots of cell lines that were at the same passage as the stocks that were used for protein preparations or one to two passages later. RNA concentrations and quality control spectrophotometric determinations were done on a NanoDrop microvolume spectrophotometer (ThermoFisher Scientific) and only samples with 260/280 & 260/230 ratios of 2.0–2.1 and 1.8–2.2 respectively were used.

RNASeq was performed by a commercial entity (BGI Americas, San Jose, CA). Briefly, total RNA was checked for quality (RIN > 9) and libraries were constructed. Libraries were 50bp single-end sequenced on a BGISEQ-500 instrument to a standard depth of 20 million reads per sample. Sequencing data was filtered and supplied as differential gene expression data sets.

### Metabolomics

Metabolomes were generated as previously described [12]: Briefly, metabolite data from all samples were acquired using Agilent 6540 Quadrupole–Time-of-Flight (Q-TOF) mass spectrometer with Agilent 1290 HPLC at the Metabolomics Facility at Johns Hopkins Medical Institution. Data was analyzed using Agilent Mass Hunter and Agilent Mass Profiler Professional (MPP) version 13.1.1 and Agilent Qualitative and Quantitative Analysis Software packages (version 6.00) to determine the metabolic profile of each sample. Aqueous phase metabolites were used in pathway analyses.

### Quantitative real-time polymerase chain reaction (qRT-PCR)

RNA was isolated from cell lines and transcribed into cDNA using manufacturer’s protocols (Qiagen, Germantown, MD and Bio-Rad, Hercules, CA). Diluted cDNA was used as template for qRT-PCR to amplify target genes in replicates of two on a thermal cycler with primer sequences given in S29 Spreadsheet. Relative change in target gene expression was calculated using the 36B4 gene as housekeeper [54].

### Pathway analyses

Pathways were identified by submitting protein, transcript, or metabolite data sets into an online interactive pathway search tool: ConsensusPathDB (cpdb.molgen.mpg.de) [26, 27]. Data sets were made up of members that were 1.25-fold changed from their corresponding control (primary-tumor: 1° tumor) members. ConcensusPathDB has integrated 32 human, 15 mouse, and 14 yeast databases into one platform, which provides a robust combined analysis of: protein interactions, signaling interactions, metabolic interactions, gene regulatory interactions, genetic interactions, drug-target interactions, and biochemical interactions [26]. Pathway analyses were initiated in ConsensusPathDB with expression enrichment data set determinations of protein, gene, or metabolite data sets that were then analyzed using the default setting of 11 integrated pathway databases, i.e.: the Kyoto Encyclopedia of Genes and Genomes (KEGG; www.genome.jp/kegg/) [55], Reactome (reactome.org) [56], the Small Molecule Pathway Data Base (SMPDB), Wikipathways (www.wikipathways.org/index.php/wikipathways), the Edinburgh Human Metabolic Network (EHMN) [57], the Pathway Interaction Database (PID) [58], the Integrating Network Objects with Hierarchies (INOH) database [59], the BioCarta database (NCI based; www.biocara.com/genes/), the Encyclopedia of Human Genes and Metabolism (HumanCyc) database (www.humancyc.org), and the PharmGKB database (www.pharmgkb.org) [60].

### Principal component analyses and hierarchical clustering

To reduce the complexity of the large amount of data that was generated from the proteomics (S1–S6 Spreadsheets) and RNA sequencing (S7–S12 Spreadsheets), and to infer relationships between the data sets, we performed principal component analysis (PCA) and hierarchical clustering at both the protein and transcript (gene) level. Hierarchical clustering was performed using Morpheus (https://software.broadinstitute.org/morpheus).

### *In vitro* drug assays

The established FDA approved chemotherapuetic drugs used were: paclitaxel (TSZ CHEM, Cat# RS036, Lot# 061916), doxorubicin (Cayman Chemical, Item# 15007, Lot# NA), and Gemcitabine (Sigma, Cat# G6423-10mg, Lot# 026M4704V). In all cases, cells were plated at 2000 cells per well onto 96 well plates and 24 hrs later treated with each drug over a serial dilution range of drugs: paclitaxel (PAC, 0.01–50 nM), doxorubicin (DOX, 0.01–5 μM), gemcitabine (GEM, 0.001–10 μM), and an in-house DDX3X inhibitor drug RK-33 (1–25 μM). Each concentration of drug was added to cells (wells) in quadruplicate along with no drug added control wells. Two to three biologic replicates were done. Standard colorimetric MTS assays (addition of 10% MTS reagent in medium with a 2 hr incubation) were done 72 hrs after drug treatment. Plots of the spectrophotometric outputs (absorbance vs log[drug]) were used to determine the IC_50_ values of each drug.

### Statistical methods

As described above, all **Proteomics** and **Metabolomics** source datasets were generated at core facilities at the Johns Hopkins University while an outside company generated the **RNAseq** datasets. As such, we received datasets with completed statistical analyses and all p- and or q-values presented in all Tables and Spreadsheets were obtained from the source datasets. In the case of **Pathway analyses**, we utilized the online database, ConsensusPathDB, which has published the statistical methods used [26, 27], as described above. For the ***In vitro* drug assay**-IC_50_ values dataset, we applied F tests to determine unequal or equal variances and then the appropriate two-sided Student’s *t*-tests (*p* ≤ 0.05) were utilized to evaluate significant differences.

## Supporting information

S1 Fig Phase-contrast images (2 fields of view) of the parental-435, primary tumor (1° tumor)-435, brain-435, liver-435, lung-435, and spine-435 cell lines are shown. The images were photographed using a X10 objective coupled with a X4 phase-contrast ring. This optical configuration gave 3D images. The black scale bars = 50 μM.
(TIFF)

S2 Fig Phase-contrast images (2 fields of view) of the parental-231, primary tumor (1° tumor)-231, lung-231, and lymph node-231 cell lines are shown. The images were photographed using a X10 objective coupled with a X10 phase-contrast ring. The black scale bars = 50 μM.
(TIFF)

S3 Fig Growth curves of MDA-MB-435 cell lines with the mean growth-rate given in the bottom right-hand corner of the curves except for brain where two distinct growth rates are presented near the curve. (TIFF)

S4 Fig The up- and down-regulated proteomic-based interconnected network maps of pathways unique to the liver-435 cell line. The size range of the nodes correlates to the size of the protein sets while the range of hues of the nodes correlates with the q-values, which is correlated to the size of the number of observed proteins. The edges represent the overlap of shared proteins between the connected nodes with the width of the edges representative of the size of the overlap and their color denoting the number of the observed proteins that are shared.
(TIFF)

S5 Fig The up- and down-regulated proteomic-based interconnected pathway network maps of unique to the lung-435 cell line. The size range of the nodes correlates to the size of the protein sets while the range of hues of the nodes correlates with the q-values, which is correlated to the size of the number of observed proteins. The edges represent the overlap of shared proteins between the connected nodes with the width of the edges representative of the size of the overlap and their color denoting the number of the observed proteins that are shared.
(TIFF)

S6 Fig The up- and down-regulated proteomic-based interconnected pathway network maps of unique to the spine-435 cell line. The size range of the nodes correlates to the size of the protein sets while the range of hues of the nodes correlates with the q-values, which is correlated to the size of the number of observed proteins. The edges represent the overlap of shared proteins of the connected nodes with the width of the edges representative of the size of the overlap and their color denoting the number of the observed proteins that are shared.
(TIFF)

S7 Fig The up- and down-regulated proteomic-based interconnected pathway network maps of unique to the lymph node-231 cell line. The size range of the nodes correlates to the size of the protein sets while the range of hues of the nodes correlates with the q-values, which is correlated to the size of the number of observed proteins. The edges represent the overlap of shared proteins of the connected nodes with the width of the edges representative of the size of the overlap and their color denoting the number of the observed proteins that are shared.
(TIFF)

S8 Fig The up- and down-regulated transcriptomic-based interconnected pathway network maps of unique to the liver-435 cell line. The size range of the nodes correlates to the size of the transcript (gene) sets while the range of hues of the nodes correlates with the q-values, which is correlated to the size of the number of observed transcripts. The edges represent the overlap of shared transcripts of the connected nodes with the width of the edges representative of the size of the overlap and their color denoting the number of the observed transcripts that are shared.
(TIFF)

S9 Fig The up- and down-regulated transcriptomic-based interconnected pathway network maps of unique to the lung-435 cell line. The size range of the nodes correlates to the size of the transcript (gene) sets while the range of hues of the nodes correlates with the q-values, which is correlated to the size of the number of observed transcripts. The edges represent the overlap of shared transcripts of the connected nodes with the width of the edges representative of the size of the overlap and their color denoting the number of the observed transcripts that are shared.
(TIFF)

S10 Fig The up- and down-regulated transcriptomic-based interconnected pathway network maps of unique to the spine-435 cell line. The size range of the nodes correlates to the size of the transcript (gene) sets while the range of hues of the nodes correlates with the q-values, which is correlated to the size of the number of observed transcripts. The edges represent the overlap of shared transcripts of the connected nodes with the width of the edges representative of the size of the overlap and their color denoting the number of the observed transcripts that are shared.
(TIFF)

S11 Fig The up- and down-regulated transcriptomic-based interconnected pathway network maps of unique to the lymph node-231 cell line. The size range of the nodes correlates to the size of the transcript (gene) sets while the range of hues of the nodes correlates with the q-values, which is correlated to the size of the number of observed transcripts. The edges represent the overlap of shared transcripts of the connected nodes with the width of the edges representative of the size of the overlap and their color denoting the number of the observed transcripts that are shared.
(TIFF)

S1 Table Proteomic-based pathway discovery for the metastatic brain-435 cell line. (DOCX)

S2 Table Proteomic-based pathway discovery for the metastatic liver-435 cell line. (DOCX)

S3 Table Proteomic-based pathway discovery for the metastatic lung-435 cell line. (DOCX)

S4 Table Proteomic-based pathway discovery for the metastatic spine-435 cell line. (DOCX)

S5 Table Proteomic-based pathway discovery for the metastatic Lung-231 cell line. (DOCX)

S6 Table Proteomic-based pathway discovery for the metastatic lymph node-231 cell line. (DOCX)

S7 Table Transcriptomic-based pathway discovery for the metastatic brain-435 cell line. (DOCX)

S8 Table Transcriptomic-based pathway discovery for the metastatic liver-435 cell line. (DOCX)

S9 Table Transcriptomic-based pathway discovery for the metastatic lung-435 cell line. (DOCX)

S10 Table Transcriptomic-based pathway discovery for the metastatic spine-435 cell line. (DOCX)

S11 Table Transcritomic-based pathway discovery for the metastatic lung-231 cell line. (DOCX)

S12 Table Transcriptomic-based pathway discovery for the metastatic lymph node-231 cell line. (DOCX)

S13 Table Transcriptomic-based Unique pathways for the metastatic brain-435 cell line. (DOCX)

S14 Table Transcriptomic-based Unique pathways for the metastatic liver-435 cell line. (DOCX)

S15 Table Transcriptomic-based Unique pathways for the metastatic lung-435 cell line. (DOCX)

S16 Table Transcriptomic-based Unique pathways for the metastatic spine-435 cell line. (DOCX)

S17 Table Transcriptomic-based Unique pathways for the metastatic lung-231 cell line. (DOCX)

S18 Table Transcriptomic-based Unique pathways for the metastatic lymph node-231 cell line. (DOCX)

S19 Table Common proteome and transcriptome pathways for the metastatic brain-435 cell line. (DOCX)

S20 Table Common proteome and transcriptome pathways for the metastatic liver-435 cell line. (DOCX)

S21 Table Common proteome and transcriptome pathways for the metastatic lung-435 cell line. (DOCX)

S22 Table Common proteome and transcriptome pathways for the metastatic spine-435 cell line. (DOCX)

S23 Table Common proteome and transcriptome pathways for the metastatic lung-231 cell line. (DOCX)

S24 Table Common proteome and transcriptome pathways for the metastatic lymph node-231 cell line. (DOCX)

S25 Table Metabolomic-based pathway discovery for the metastatic brain-435 cell line. (DOCX)

S26 Table Metabolomic-based pathway discovery for the metastatic liver-435 cell line. (DOCX)

S27 Table Metabolomic-based pathway discovery for the metastatic lung-435 cell line. (DOCX)

S28 Table Metabolomic-based pathway discovery for the metastatic spine-435 cell line. (DOCX)

S29 Table Metabolomic-based Unique pathways for the metastatic brain-435 cell line. (DOCX)

S30 Table Metabolomic-based Unique pathways for the metastatic liver-435 cell line. (DOCX)

S31 Table Metabolomic-based Unique pathways for the metastatic liver-435 cell line. (DOCX)

S32 Table Metabolomic-based Unique pathways for the metastatic spine-435 cell line. (DOCX)

S33 Table Common metabolomic and proteomic pathways for the metastatic brain-435 cell line. (DOCX)

S34 Table Common metabolomic and proteomic pathways for the metastatic liver-435 cell line. (DOCX)

S35 Table Common metabolomic and proteomic pathways for the metastatic lung-435 cell line. (DOCX)

S36 Table Common metabolomic and proteomic pathways for the metastatic spine-435 cell line. (DOCX)

S37 Table Common metabolomic and transcriptomic pathways for the metastatic brain-435 cell line. (DOCX)

S38 Table Common metabolomic and transcriptomic pathways for the metastatic liver-435 cell line. (DOCX)

S39 Table Common metabolomic and transcriptomic pathways for the metastatic lung-435 cell line. (DOCX)

S40 Table Common metabolomic and transcriptomic pathways for the metastatic spine-435 cell line. (DOCX)

S41 Table Proteomic-based pathways found to be up & down for the metastatic brain-435 cell line. (DOCX)

S42 Table Proteomic-based pathways found to be up & down for the metastatic liver-435 cell line. (DOCX)

S43 Table Proteomic-based pathways found to be up & down for the metastatic lung-435 cell line. (DOCX)

S44 Table Proteomic-based pathways found to be up & down for the metastatic spine-435 cell line. (DOCX)

S45 Table Proteomic-based pathways found to be up & down for the metastatic lung-231 cell line. (DOCX)

S46 Table Proteomic-based pathways found to be up & down for the metastatic lymph node-231 cell line. (DOCX)

S1 Spreadsheet Comprehensive information for proteomics for all metastatic cell lines including linear fold changes relative to the primary tumors. (XLSX)

S2 Spreadsheet Comprehensive information for proteomics for all metastatic cell lines including linear fold changes relative to the primary tumors. (XLSX)

S3 Spreadsheet Comprehensive information for proteomics for all metastatic cell lines including linear fold changes relative to the primary tumors. (XLSX)

S4 Spreadsheet Comprehensive information for proteomics for all metastatic cell lines including linear fold changes relative to the primary tumors. (XLSX)

S5 Spreadsheet Comprehensive information for proteomics for all metastatic cell lines including linear fold changes relative to the primary tumors. (XLSX)

S6 Spreadsheet Comprehensive information for proteomics for all metastatic cell lines including linear fold changes relative to the primary tumors. (XLSX)

S7 Spreadsheet Comprehensive information for transcriptomics across all metastatic cell lines including linear fold changes relative to the primary tumors. (XLSX)

S8 Spreadsheet Comprehensive information for transcriptomics across all metastatic cell lines including linear fold changes relative to the primary tumors. (XLSX)

S9 Spreadsheet Comprehensive information for transcriptomics across all metastatic cell lines including linear fold changes relative to the primary tumors. (XLSX)

S10 Spreadsheet Comprehensive information for transcriptomics across all metastatic cell lines including linear fold changes relative to the primary tumors. (XLSX)

S11 Spreadsheet Comprehensive information for transcriptomics across all metastatic cell lines including linear fold changes relative to the primary tumors. (XLSX)

S12 Spreadsheet Comprehensive information for transcriptomics across all metastatic cell lines including linear fold changes relative to the primary tumors. (XLSX)

S13 Spreadsheet Comprehensive information for proteomics for all six metastatic cell lines that fall within ≤ -1.25 and ≥ 1.25 linear fold change range relative to the primary tumors. (XLSX)

S14 Spreadsheet Comprehensive information for proteomics for all six metastatic cell lines that fall within ≤ -1.25 and ≥ 1.25 linear fold change range relative to the primary tumors. (XLSX)

S15 Spreadsheet Comprehensive information for proteomics for all six metastatic cell lines that fall within ≤ -1.25 and ≥ 1.25 linear fold change range relative to the primary tumors. (XLSX)

S16 Spreadsheet Comprehensive information for proteomics for all six metastatic cell lines that fall within ≤ -1.25 and ≥ 1.25 linear fold change range relative to the primary tumors. (XLSX)

S17 Spreadsheet Comprehensive information for proteomics for all six metastatic cell lines that fall within ≤ -1.25 and ≥ 1.25 linear fold change range relative to the primary tumors. (XLSX)

S18 Spreadsheet Comprehensive information for proteomics for all six metastatic cell lines that fall within ≤ -1.25 and ≥ 1.25 linear fold change range relative to the primary tumors. (XLSX)

S19 Spreadsheet Comparison of proteomic derived pathways across all metastatic cell lines. (XLSX)

S20 Spreadsheet Comparison of transcriptomic derived pathways across all metastatic cell lines. (XLSX)

S21 Spreadsheet All brain-435 metabolic pathways derived from metabolomics at the ≥ 1.25 linear fold change and separately common to proteomic derived pathways and RNAseq derived pathways. (XLSX)

S22 Spreadsheet All brain-435 metabolic pathways derived from metabolomics at the ≤ -1.25 linear fold change and separately common to proteomic derived pathways and RNAseq derived pathways. (XLSX)

S23 Spreadsheet All liver-435 metabolic pathways derived from metabolomics at the ≥ 1.25 linear fold change and separately common to proteomic derived pathways and RNAseq derived pathways. (XLSX)

S24 Spreadsheet All liver-435 metabolic pathways derived from metabolomics at the ≤ -1.25 linear fold change and separately common to proteomic derived pathways and RNAseq derived pathways. (XLSX)

S25 Spreadsheet All lung-435 metabolic pathways derived from metabolomics at the ≥ 1.25 linear fold change and separately common to proteomic derived pathways and RNAseq derived pathways. (XLSX)

S26 Spreadsheet All lung-435 metabolic pathways derived from metabolomics at the ≤ -1.25 linear fold change and separately common to proteomic derived pathways and RNAseq derived pathways. (XLSX)

S27 Spreadsheet All spine-435 metabolic pathways derived from metabolomics at the ≥ 1.25 linear fold change and separately common to proteomic derived pathways and RNAseq derived pathways. (XLSX)

S28 Spreadsheet All spine-435 metabolic pathways derived from metabolomics at the ≤ -1.25 linear fold change and separately common to proteomic derived pathways and RNAseq derived pathways. (XLSX)

S29 Spreadsheet qRT-PCR primer sets. (XLSX)
