# Supplementary material for: Divergent organ-specific isogenic metastatic cell lines identified using multi-omics exhibit differential drug sensitivity
Source: PLoS One. 2020 Nov 16;15(11):e0242384. doi: 10.1371/journal.pone.0242384 (PMC7668614; doi:10.1371/journal.pone.0242384)
Supplement: S1 Table — (DOCX) [file pone.0242384.s012.docx]

| **S1 Table. Proteomic-based pathway discovery for the metastatic Brain-435 cell line.** | | | | | |  |
| --- | --- | --- | --- | --- | --- | --- |
| **Source** | **Up Pathways** | **# of Proteins in Set** | **# of Obs. Proteins** | **Obs. Proteins (%)** | **q-value** | |
| SMPDB | Oncogenic Action of Succinate | 31 | 9 | 29.0 | 0.0005 | |
| SMPDB | Oncogenic Action of Fumarate | 31 | 9 | 29.0 | 0.0005 | |
| NetPath | EGFR1 | 457 | 37 | 8.1 | 0.0007 | |
| Reactome | Citric Acid Cycle | 22 | 7 | 31.8 | 0.0022 | |
| KEGG | Spliceosome | 134 | 16 | 11.9 | 0.0030 | |
| Wikipathways | Amino Acid Metabolism | 91 | 13 | 14.3 | 0.0030 | |
| Reactome | Mitochondrial Protein Import | 65 | 11 | 16.9 | 0.0030 | |
| Wikipathways | TCA Cycle | 17 | 6 | 35.3 | 0.0030 | |
| KEGG | Lysosome | 123 | 15 | 12.2 | 0.0031 | |
| EHMN | Propanoate Metabolism | 18 | 6 | 33.3 | 0.0031 | |
|  | **Down Pathways** |  |  |  |  | |
| Reactome | Cell Cycle | 564 | 94 | 16.7 | 1.93E-13 | |
| Reactome | Cell Cycle, Mitotic | 481 | 81 | 16.9 | 8.43E-12 | |
| NetPath | TNF-α | 234 | 44 | 18.8 | 2.97E-07 | |
| Wikipathways | Pyrimidine Metabolism | 84 | 24 | 28.6 | 4.25E-07 | |
| Reactome | Cell Cycle Checkpoints | 250 | 44 | 17.7 | 1.36E-06 | |
| Reactome | Translation | 310 | 50 | 16.3 | 1.57E-06 | |
| Reactome | Mitotic Spindle Checkpoint | 113 | 27 | 23.9 | 1.57E-06 | |
| PID | ATR Signaling Pathway | 37 | 15 | 40.5 | 1.57E-06 | |
| KEGG | Pyrimidine metabolism | 101 | 25 | 24.8 | 2.19E-06 | |
| Reactome | Mitotic Anaphase | 140 | 30 | 21.4 | 2.26E-06 | |
