# Supplementary material for: Divergent organ-specific isogenic metastatic cell lines identified using multi-omics exhibit differential drug sensitivity
Source: PLoS One. 2020 Nov 16;15(11):e0242384. doi: 10.1371/journal.pone.0242384 (PMC7668614; doi:10.1371/journal.pone.0242384)
Supplement: S2 Table — (DOCX) [file pone.0242384.s013.docx]

| **S2 Table.** **Proteomic-based pathway discovery for the metastatic Liver-435 cell line.** | | | | | |  |
| --- | --- | --- | --- | --- | --- | --- |
| **Source** | **Up Pathways** | **# of Proteins in Set** | **# of Obs. Proteins** | **Obs. Proteins (%)** | **q-value** | |
| KEGG | Lysosome | 123 | 16 | 13.0 | 3.85E-09 | |
| Reactome | Regulation of IGF Transport & Uptake by IGFBPs | 127 | 11 | 8.7 | 0.000201 | |
| Reactome | MHC Class II Antigen Presentation | 59 | 8 | 13.6 | 0.000201 | |
| Reactome | Vesicle-mediated Transport | 620 | 24 | 3.9 | 0.000345 | |
| Reactome | Neutrophil Degranulation | 490 | 20 | 4.1 | 0.000814 | |
| SMPDB | Vitamin K Metabolism | 4 | 3 | 75.0 | 0.000882 | |
| Reactome | Formation of Fibrin Clot | 39 | 6 | 15.4 | 0.000953 | |
| Reactome | Post-translational Protein Phosphorylation | 110 | 9 | 8.3 | 0.001048 | |
| Reactome | AUF1 (hnRNP D0) Binds & Destabilizes mRNA | 6 | 3 | 50.0 | 0.002883 | |
| Wikipathways | Ebola Virus Pathway on Host | 130 | 9 | 6.9 | 0.003410 | |
|  | **Down Pathways** |  |  |  |  | |
| Reactome | Cell Cycle | 564 | 62 | 11.0 | 7.73E-07 | |
| Wikipathways | Pyrimidine Metabolism | 84 | 20 | 23.8 | 1.12E-06 | |
| Reactome | Prefoldin Mediated Transfer of Substrate to CCT/TriC | 28 | 12 | 42.9 | 1.12E-06 | |
| KEGG | Proteasome | 45 | 14 | 31.1 | 5.14E-06 | |
| Reactome | Prefoldin & TriC/CCT in Actin & Tubulin Folding | 33 | 12 | 36.4 | 6.32E-06 | |
| Reactome | Cds1 Mediated Inactivation of Cyclin B:Cdk1 Complex | 13 | 8 | 61.5 | 8.10E-06 | |
| Wikipathways | Proteasome Degradation | 64 | 16 | 25.0 | 8.64E-06 | |
| KEGG | Pyrimidine Metabolism | 101 | 20 | 19.8 | 1.06E-05 | |
| Reactome | Folding of Actin by CCT/TriC | 10 | 7 | 70.0 | 1.17E-05 | |
| Reactome | Cell Cycle, Mitotic | 481 | 50 | 10.4 | 2.46E-05 | |
