# Supplementary material for: Divergent organ-specific isogenic metastatic cell lines identified using multi-omics exhibit differential drug sensitivity
Source: PLoS One. 2020 Nov 16;15(11):e0242384. doi: 10.1371/journal.pone.0242384 (PMC7668614; doi:10.1371/journal.pone.0242384)
Supplement: S3 Table — (DOCX) [file pone.0242384.s014.docx]

| **S3 Table. Proteomic-based pathway discovery for the metastatic Lung-435 cell line.** | | | | | |  |
| --- | --- | --- | --- | --- | --- | --- |
| **Source** | **Up Pathways** | **# of Proteins in Set** | **# of Obs. Proteins** | **Obs. Proteins (%)** | **q-value** | |
| Reactome | Interferon Signaling | 158 | 31 | 19.6 | 1.41E-10 | |
| Reactome | TCA Cycle & Respiratory Electron Transport | 173 | 31 | 17.9 | 9.03E-10 | |
| Reactome | Respiratory Electron Transport | 100 | 23 | 23.0 | 2.45E-09 | |
| KEGG | Parkinson Disease | 142 | 27 | 19.0 | 3.12E-09 | |
| Reactome | Interferon-γ Signaling | 94 | 21 | 22.3 | 2.27E-08 | |
| KEGG | Nonalcoholic Fatty Liver Disease | 149 | 26 | 17.4 | 3.78E-08 | |
| Wikipathways | Nonalcoholic Fatty Liver Disease | 155 | 26 | 16.8 | 7.95E-08 | |
| Reactome | Respiratory Electron Transport, ATP Synthesis by Chemiosmotic Coupling, & Heat Production by Uncoupling Proteins | 123 | 23 | 18.7 | 7.95E-08 | |
| Reactome | Metabolism | 1972 | 129 | 6.6 | 1.76E-07 | |
| KEGG | Alzheimer Disease | 171 | 26 | 15.2 | 4.94E-07 | |
|  | **Down Pathways** |  |  |  |  | |
| NetPath | EGFR1 | 457 | 72 | 15.8% | 5.34E-13 | |
| Reactome | Regulation of IGF transport & Uptake by Insulin-like IGFBPs | 127 | 32 | 25.4% | 1.82E-10 | |
| Reactome | Platelet Activation, Signaling & Aggregation | 260 | 47 | 18.1% | 2.65E-10 | |
| Reactome | Response to Elevated Platelet Cytosolic Ca^2+^ | 134 | 32 | 23.9% | 5.69E-10 | |
| Reactome | Post-translational Protein Phosphorylation | 110 | 28 | 25.7% | 1.81E-09 | |
| Reactome | Metabolism of Proteins | 2008 | 178 | 8.9% | 3.00E-09 | |
| Reactome | Platelet Degranulation | 129 | 30 | 23.3% | 3.47E-09 | |
| Reactome | Hemostasis | 668 | 78 | 11.7% | 4.21E-08 | |
| KEGG | Focal adhesion | 199 | 35 | 17.6% | 2.10E-07 | |
| Reactome | Neutrophil Degranulation | 490 | 61 | 12.6% | 2.36E-07 | |
