# Supplementary material for: Divergent organ-specific isogenic metastatic cell lines identified using multi-omics exhibit differential drug sensitivity
Source: PLoS One. 2020 Nov 16;15(11):e0242384. doi: 10.1371/journal.pone.0242384 (PMC7668614; doi:10.1371/journal.pone.0242384)
Supplement: S4 Table — (DOCX) [file pone.0242384.s015.docx]

| **S4 Table. Proteomic-based pathway discovery for the metastatic Spine-435 cell line.** | | | | | |  |
| --- | --- | --- | --- | --- | --- | --- |
| **Source** | **Up Pathways** | **# of Proteins in Set** | **# of Obs. Proteins** | **Obs. Proteins (%)** | **q-value** | |
| Reactome | Chromatin Modifying Enzymes | 272 | 17 | 6.2 | 0.0012 | |
| Reactome | Chromatin Organization | 272 | 17 | 6.2 | 0.0012 | |
| Wikipathways | Cholesterol Biosynthesis, Regulation & Transport | 9 | 4 | 44.4 | 0.0023 | |
| Wikipathways | Activation of Gene Expression by SREBF (SREBP) | 20 | 5 | 25.0 | 0.0030 | |
| Reactome | Cellular Responses to Stress | 345 | 16 | 4.7 | 0.0095 | |
| Reactome | Cellular Senescence | 189 | 11 | 5.9 | 0.0095 | |
| Reactome | Activation of Gene Expression by SREBF (SREBP) | 26 | 5 | 19.2 | 0.0095 | |
| Reactome | Regulation of Cholesterol Biosynthesis by SREBP | 31 | 5 | 16.1 | 0.0095 | |
| Wikipathways | Ethanol Effects on Histone Modifications | 31 | 5 | 16.1 | 0.0095 | |
| Reactome | Amino Acid Transport Across the Plasma Membrane | 32 | 5 | 15.6 | 0.0095 | |
|  | **Down Pathways** |  |  |  |  | |
| HumanCyc | Superpathway of Conversion of Glucose to Acetyl CoA & Entry into the TCA Cycle | 48 | 15 | 31.9 | 1.03E-07 | |
| HumanCyc | Glycolysis | 25 | 11 | 45.8 | 2.05E-07 | |
| Reactome | Glycolysis | 71 | 17 | 24.3 | 2.20E-07 | |
| Reactome | Metabolism of Carbohydrates | 264 | 32 | 12.2 | 9.55E-07 | |
| Reactome | Glucose Metabolism | 91 | 18 | 20.0 | 1.18E-06 | |
| INOH | Glycolysis & Gluconeogenesis | 46 | 13 | 28.9 | 1.33E-06 | |
| Reactome | Neutrophil Degranulation | 490 | 45 | 9.3 | 2.01E-06 | |
| Reactome | Common Pathway of Fibrin Clot Formation | 22 | 9 | 40.9 | 8.01E-06 | |
| Wikipathways | Glycolysis & Gluconeogenesis | 45 | 12 | 26.7 | 9.57E-06 | |
| Wikipathways | Glycolysis Pathway D (2) | 23 | 9 | 39.1 | 1.02E-05 | |
