# Supplementary material for: Divergent organ-specific isogenic metastatic cell lines identified using multi-omics exhibit differential drug sensitivity
Source: PLoS One. 2020 Nov 16;15(11):e0242384. doi: 10.1371/journal.pone.0242384 (PMC7668614; doi:10.1371/journal.pone.0242384)
Supplement: S5 Table — (DOCX) [file pone.0242384.s016.docx]

| **S5 Table. Proteomic-based pathway discovery for the metastatic Lung-231 cell line.** | | | | | |  |
| --- | --- | --- | --- | --- | --- | --- |
| **Source** | **Up Pathways** | **# of Proteins in Set** | **# of Obs. Proteins** | **Obs. Proteins (%)** | **q-value** | |
| Reactome | Metabolism of RNA | 586 | 151 | 25.9 | 1.35E-39 | |
| Reactome | Cell Cycle | 564 | 121 | 21.5 | 3.64E-23 | |
| Reactome | Cell Cycle, Mitotic | 481 | 104 | 21.7 | 5.38E-20 | |
| Reactome | Processing of Capped Intron-Containing Pre-mRNA | 240 | 68 | 28.3 | 3.28E-19 | |
| KEGG | DNA Replication | 36 | 24 | 66.7 | 3.51E-16 | |
| Wikipathways | Retinoblastoma Gene in Cancer | 89 | 37 | 41.6 | 4.12E-16 | |
| Reactome | mRNA Splicing - Major Pathway | 178 | 53 | 29.8 | 6.46E-16 | |
| Reactome | Translation | 310 | 71 | 23.1 | 4.28E-15 | |
| Reactome | mRNA Splicing | 186 | 53 | 28.5 | 4.28E-15 | |
| KEGG | Ribosome | 153 | 45 | 29.4 | 2.92E-13 | |
|  | **Down Pathways** |  |  |  |  | |
| NetPath | EGFR1 | 457 | 85 | 18.7 | 2.15E-16 | |
| Reactome | Neutrophil Degranulation | 490 | 81 | 16.7 | 8.01E-13 | |
| Reactome | Metabolism | 1972 | 206 | 10.5 | 2.20E-11 | |
| Reactome | Vesicle-mediated Transport | 620 | 88 | 14.2 | 3.94E-10 | |
| Reactome | Membrane Trafficking | 582 | 82 | 14.1 | 3.03E-09 | |
| Reactome | Response to Elevated Platelet Cytosolic Ca^2+^ | 134 | 33 | 24.6 | 3.92E-09 | |
| Reactome | Platelet Degranulation | 129 | 32 | 24.8 | 5.63E-09 | |
| Wikipathways | miR-targeted Genes in Muscle Cell - TarBase | 400 | 62 | 15.5 | 1.59E-08 | |
| Reactome | Post-translational Protein Phosphorylation | 110 | 28 | 25.7 | 3.34E-08 | |
| Reactome | Innate Immune System | 1077 | 121 | 11.4 | 3.69E-08 | |
