# Supplementary material for: Divergent organ-specific isogenic metastatic cell lines identified using multi-omics exhibit differential drug sensitivity
Source: PLoS One. 2020 Nov 16;15(11):e0242384. doi: 10.1371/journal.pone.0242384 (PMC7668614; doi:10.1371/journal.pone.0242384)
Supplement: S6 Table — (DOCX) [file pone.0242384.s017.docx]

| **S6 Table**. Proteomic-based pathway discovery for the metastatic Lymph Node-231 cell line. | | | | | |  |
| --- | --- | --- | --- | --- | --- | --- |
| **Source** | **Up Pathways** | **# of Proteins in Set** | **# of Obs. Proteins** | **Obs. Proteins (%)** | **q-value** | |
| Reactome | Processing of Capped Intron-Containing Pre-mRNA | 240 | 82 | 34.2 | 6.66E-35 | |
| Reactome | mRNA Splicing - Major Pathway | 178 | 69 | 38.8 | 2.71E-33 | |
| Reactome | mRNA Splicing | 186 | 69 | 37.1 | 4.95E-32 | |
| Reactome | Metabolism of RNA | 586 | 123 | 21.1 | 2.50E-30 | |
| Reactome | Cell Cycle | 564 | 115 | 20.4 | 5.57E-27 | |
| KEGG | Spliceosome | 134 | 52 | 38.8 | 4.38E-25 | |
| Reactome | Cell Cycle, Mitotic | 481 | 99 | 20.6 | 2.30E-23 | |
| Wikipathways | mRNA Processing | 127 | 42 | 33.1 | 4.83E-17 | |
| Reactome | Cleavage of Growing Transcript in the Termination Region | 67 | 27 | 40.3 | 3.91E-13 | |
| Reactome | RNA Polymerase II Transcription Termination | 67 | 27 | 40.3 | 3.91E-13 | |
|  | **Down Pathways** |  |  |  |  | |
| Reactome | Metabolism | 1972 | 300 | 15.3 | 1.03E-19 | |
| Reactome | Vesicle-mediated Transport | 620 | 125 | 20.2 | 9.31E-16 | |
| Reactome | Membrane Trafficking | 582 | 120 | 20.6 | 9.31E-16 | |
| NetPath | EGFR1 | 457 | 102 | 22.4 | 9.31E-16 | |
| KEGG | Lysosome | 123 | 45 | 36.6 | 1.87E-14 | |
| Reactome | Neutrophil Degranulation | 490 | 101 | 20.8 | 1.69E-13 | |
| Reactome | Metabolism of Carbohydrates | 264 | 65 | 24.7 | 8.40E-12 | |
| Reactome | Asparagine N-Linked Glycosylation | 286 | 66 | 23.2 | 1.11E-10 | |
| Reactome | Post-translational Protein Phosphorylation | 110 | 37 | 33.9 | 1.11E-10 | |
| Reactome | Regulation of IGF Transport & Uptake by Insulin-like IGFBPs | 127 | 39 | 31.0 | 5.82E-10 | |
