# Supplementary material for: Divergent organ-specific isogenic metastatic cell lines identified using multi-omics exhibit differential drug sensitivity
Source: PLoS One. 2020 Nov 16;15(11):e0242384. doi: 10.1371/journal.pone.0242384 (PMC7668614; doi:10.1371/journal.pone.0242384)
Supplement: S7 Table — (DOCX) [file pone.0242384.s018.docx]

| **S7 Table**. Transcriptomic-based pathway discovery for the metastatic Brain-435 cell line. | | | | | | |  |
| --- | --- | --- | --- | --- | --- | --- | --- |
| **Source** | **Up Pathways** | **# of Genes in Set** | **# of Obs. Genes** | **Obs. Genes (%)** | | **q-value** | |
| KEGG | ECM-receptor Interaction | 82 | 19 | 23.2 | | 6.76E-08 | |
| PID | β1-Integrin Cell Surface Interactions | 66 | 16 | 24.2 | | 3.79E-07 | |
| Reactome | Collagen Chain Trimerization | 44 | 12 | 27.3 | | 2.87E-06 | |
| Reactome | Extracellular Matrix Organization | 294 | 36 | 12.2 | | 5.22E-06 | |
| Wikipathways | MAPK Signaling Pathway | 246 | 30 | 12.2 | | 3.41E-05 | |
| Reactome | Cardiac Conduction | 131 | 20 | 15.3 | | 2.98E-05 | |
| Reactome | Amyloid Fiber Formation | 82 | 15 | 18.5 | | 2.97E-05 | |
| Reactome | Meiotic Recombination | 65 | 13 | 20.3 | | 3.63E-05 | |
| Reactome | Laminin Interactions | 23 | 8 | 34.8 | | 1.87E-05 | |
| Reactome | Presynaptic Depolarization & Calcium Channel Opening | 13 | 6 | 46.2 | | 3.32E-05 | |
|  | **Down Pathways** |  |  |  |  | |  |
| Reactome | DNA Strand Elongation | 32 | 13 | 40.6 | 8.91E-10 | |  |
| Reactome | DNA Replication | 80 | 18 | 22.5 | 1.27E-09 | |  |
| Wikipathways | DNA Replication | 42 | 14 | 33.3 | 1.27E-09 | |  |
| Reactome | Synthesis of DNA | 75 | 17 | 22.7 | 3.37E-09 | |  |
| Reactome | Cell Cycle | 564 | 46 | 8.2 | 3.70E-09 | |  |
| Reactome | Cell Cycle, Mitotic | 481 | 42 | 8.8 | 3.70E-09 | |  |
| Wikipathways | Retinoblastoma Gene in Cancer | 89 | 18 | 20.2 | 3.70E-09 | |  |
| Reactome | Activation of the Pre-Replicative Complex | 33 | 11 | 33.3 | 8.31E-08 | |  |
| Reactome | S Phase | 103 | 17 | 16.5 | 2.42E-07 | |  |
| Reactome | DNA Replication Pre-Initiation | 37 | 11 | 29.7 | 2.42E-07 | |  |
