# Supplementary material for: Divergent organ-specific isogenic metastatic cell lines identified using multi-omics exhibit differential drug sensitivity
Source: PLoS One. 2020 Nov 16;15(11):e0242384. doi: 10.1371/journal.pone.0242384 (PMC7668614; doi:10.1371/journal.pone.0242384)
Supplement: S8 Table — (DOCX) [file pone.0242384.s019.docx]

| **S8 Table. Transcriptomic-based pathway discovery for the metastatic Liver-435 cell line.** | | | | | |
| --- | --- | --- | --- | --- | --- |
| **Source** | **Up Pathways** | **# of Genes in Set** | **# of Obs. Genes** | **Obs. Genes (%)** | **q-value** |
| Reactome | Interferon Signaling | 158 | 56 | 35.4 | 1.18E-20 |
| Reactome | Extracellular Matrix Organization | 294 | 71 | 24.1 | 2.01E-15 |
| Reactome | Interferon-γ Signaling | 94 | 36 | 38.3 | 5.84E-15 |
| Reactome | Interferon α/β Signaling | 70 | 30 | 42.9 | 3.06E-14 |
| Reactome | Cytokine Signaling in Immune system | 458 | 88 | 19.2 | 1.51E-12 |
| Wikipathways | Interferon α−β Signaling | 21 | 15 | 71.4 | 4.79E-12 |
| Reactome | Immune System | 1840 | 242 | 13.2 | 8.13E-12 |
| KEGG | Lysosome | 123 | 35 | 28.5 | 2.52E-10 |
| KEGG | Antigen Processing & Presentation | 77 | 26 | 33.8 | 8.86E-10 |
| Wikipathways | Ebola Virus Pathway on Host | 130 | 35 | 26.9 | 1.31E-09 |
|  | **Down Pathways** |  |  |  |  |
| Reactome | Cell Cycle | 564 | 159 | 28.2 | 3.16E-56 |
| Reactome | Cell Cycle, Mitotic | 481 | 138 | 28.8 | 2.09E-49 |
| Wikipathways | Retinoblastoma Gene in Cancer | 89 | 49 | 55.1 | 1.53E-31 |
| Reactome | Cell Cycle Checkpoints | 250 | 78 | 31.3 | 1.09E-29 |
| Reactome | Mitotic Prometaphase | 186 | 67 | 36.0 | 1.72E-29 |
| Reactome | M Phase | 340 | 87 | 25.7 | 2.24E-26 |
| Reactome | DNA Repair | 320 | 77 | 24.3 | 1.50E-21 |
| Reactome | S Phase | 103 | 42 | 40.8 | 1.40E-20 |
| Reactome | DNA Double-Strand Break Repair | 169 | 53 | 31.7 | 2.96E-20 |
| Reactome | HDR through Homologous Recombination (HRR) | 69 | 34 | 50.0 | 4.43E-20 |
