# Supplementary material for: Divergent organ-specific isogenic metastatic cell lines identified using multi-omics exhibit differential drug sensitivity
Source: PLoS One. 2020 Nov 16;15(11):e0242384. doi: 10.1371/journal.pone.0242384 (PMC7668614; doi:10.1371/journal.pone.0242384)
Supplement: S9 Table — (DOCX) [file pone.0242384.s020.docx]

| **S9** **Table.** **Transcriptomic-based pathway discovery for the metastatic Lung-435 cell line.** | | | | | |
| --- | --- | --- | --- | --- | --- |
| **Source** | **Up Pathways** | **# of Genes in Set** | **# of Obs. Genes** | **Obs. Genes (%)** | **q-value** |
| Reactome | Metabolism of RNA | 586 | 175 | 30.0 | 4.77E-16 |
| Reactome | Gene Expression (Transcription) | 1373 | 331 | 24.3 | 5.49E-16 |
| Reactome | RNA Polymerase II Transcription | 1236 | 300 | 24.4 | 8.99E-15 |
| Reactome | Generic Transcription Pathway | 1107 | 267 | 24.3 | 1.26E-12 |
| Reactome | rRNA Modification in the Nucleus & Cytosol | 59 | 33 | 56.9 | 2.60E-10 |
| Reactome | rRNA Processing in the Nucleus & Cytosol | 59 | 33 | 56.9 | 2.60E-10 |
| Reactome | rRNA Processing | 65 | 33 | 51.6 | 8.68E-09 |
| Reactome | Cellular Responses to External Stimuli | 414 | 109 | 26.4 | 2.20E-06 |
| Reactome | Chromatin Modifying Enzymes | 272 | 79 | 29.0 | 2.65E-06 |
| Reactome | Chromatin Organization | 272 | 79 | 29.0 | 2.65E-06 |
|  | **Down Pathways** |  |  |  |  |
| Reactome | Extracellular Matrix Organization | 294 | 89 | 30.3 | 1.32E-08 |
| Reactome | Collagen Formation | 92 | 38 | 41.3 | 7.07E-07 |
| Reactome | Collagen Biosynthesis & Modifying Enzymes | 68 | 30 | 44.1 | 4.92E-06 |
| Reactome | O-Linked Glycosylation | 116 | 36 | 31.0 | 0.004207 |
| PID | β1-Integrin Cell Surface Interactions | 66 | 24 | 36.4 | 0.004539 |
| PID | Fanconi Anemia Pathway | 46 | 19 | 41.3 | 0.004539 |
| Wikipathways | DNA Replication | 42 | 18 | 42.9 | 0.004539 |
| Reactome | DNA Strand Elongation | 32 | 15 | 46.9 | 0.005394 |
| Reactome | Axon Guidance | 358 | 82 | 23.0 | 0.005429 |
| KEGG | Axon Guidance | 175 | 47 | 26.9 | 0.005585 |
