# Supplementary material for: Divergent organ-specific isogenic metastatic cell lines identified using multi-omics exhibit differential drug sensitivity
Source: PLoS One. 2020 Nov 16;15(11):e0242384. doi: 10.1371/journal.pone.0242384 (PMC7668614; doi:10.1371/journal.pone.0242384)
Supplement: S10 Table — (DOCX) [file pone.0242384.s021.docx]

| **S10 Table. Transcriptomic-based pathway discovery for the metastatic Spine-435 cell line.** | | | | | |
| --- | --- | --- | --- | --- | --- |
| **Source** | **Up Pathways** | **# of Genes in Set** | **# of Obs. Genes** | **Obs. Genes (%)** | **q-value** |
| Wikipathways | Striated Muscle Contraction Pathway | 38 | 14 | 36.8 | 5.61E-06 |
| Reactome | Muscle Contraction | 195 | 32 | 16.4 | 7.09E-06 |
| Reactome | Striated Muscle Contraction | 35 | 12 | 34.3 | 6.42E-05 |
| HumanCyc | Superpathway of Cholesterol Biosynthesis | 25 | 9 | 36.0 | 0.000539 |
| Reactome | Cholesterol Biosynthesis | 25 | 9 | 36.0 | 0.000539 |
| SMPDB | Simvastatin Action Pathway | 22 | 8 | 36.4 | 0.000539 |
| SMPDB | Hyper-IgD Syndrome | 22 | 8 | 36.4 | 0.000539 |
| SMPDB | Cholesteryl Ester Storage Disease | 22 | 8 | 36.4 | 0.000539 |
| SMPDB | Lysosomal Acid Lipase Deficiency (Wolman Disease) | 22 | 8 | 36.4 | 0.000539 |
| SMPDB | Mevalonic Aciduria | 22 | 8 | 36.4 | 0.000539 |
|  | **Down Pathways** |  |  |  |  |
| Reactome | Collagen Formation | 92 | 26 | 28.3 | 5.21E-09 |
| Reactome | Collagen Biosynthesis & Modifying Enzymes | 68 | 19 | 27.9 | 2.82E-06 |
| Reactome | Cell Cycle | 564 | 66 | 11.7 | 3.92E-06 |
| PID | HIF-1α Transcription Factor Network | 66 | 18 | 27.3 | 5.59E-06 |
| HumanCyc | Glycolysis | 25 | 11 | 45.8 | 7.13E-06 |
| Reactome | Cell Cycle, Mitotic | 481 | 57 | 11.9 | 1.42E-05 |
| Reactome | Extracellular Matrix Organization | 294 | 40 | 13.6 | 3.73E-05 |
| Wikipathways | Glycolysis Pathway D (2) | 23 | 10 | 43.5 | 3.73E-05 |
| Wikipathways | Retinoblastoma Gene in Cancer | 89 | 19 | 21.3 | 6.98E-05 |
| Reactome | Collagen Chain Trimerization | 44 | 13 | 29.5 | 8.04E-05 |
