# Supplementary material for: Divergent organ-specific isogenic metastatic cell lines identified using multi-omics exhibit differential drug sensitivity
Source: PLoS One. 2020 Nov 16;15(11):e0242384. doi: 10.1371/journal.pone.0242384 (PMC7668614; doi:10.1371/journal.pone.0242384)
Supplement: S11 Table — (DOCX) [file pone.0242384.s022.docx]

| **S11 Table**. Transcritomic-based pathway discovery for the metastatic Lung-231 cell line. | | | | | |  |  |
| --- | --- | --- | --- | --- | --- | --- | --- |
| **Source** | **Up Pathways** | **# of Genes in Set** | **# of Obs. Genes** | **Obs. Genes (%)** | **q-value** |  |  |
| KEGG | Systemic Lupus Erythematosus | 133 | 39 | 29.5 | 3.07E-05 | |  |
| HumanCyc | Superpathway of Cholesterol Biosynthesis | 25 | 14 | 56.0 | 6.89E-05 | |  |
| Reactome | Cholesterol Biosynthesis | 25 | 14 | 56.0 | 6.89E-05 | |  |
| SMPDB | Simvastatin Action Pathway | 22 | 12 | 54.5 | 9.47E-05 | |  |
| SMPDB | Hyper-IgD Syndrome | 22 | 12 | 54.5 | 9.47E-05 | |  |
| SMPDB | Cholesteryl Ester Storage Disease | 22 | 12 | 54.5 | 9.47E-05 | |  |
| SMPDB | Lysosomal Acid Lipase Deficiency (Wolman Disease) | 22 | 12 | 54.5 | 9.47E-05 | |  |
| SMPDB | Mevalonic Aciduria | 22 | 12 | 54.5 | 9.47E-05 | |  |
| SMPDB | Wolman Disease | 22 | 12 | 54.5 | 9.47E-05 | |  |
| SMPDB | Smith-Lemli-Opitz Syndrome | 22 | 12 | 54.5 | 9.47E-05 | |  |
|  | **Down Pathways** |  |  |  |  | |  |
| Reactome | Extracellular Matrix Organization | 294 | 67 | 22.8 | 0.002189 | | |
| Reactome | Collagen Formation | 92 | 28 | 30.4 | 0.007379 | | |
| PID | β1-Integrin Cell Surface Interactions | 66 | 22 | 33.3 | 0.007463 | | |
| Reactome | Assembly of Collagen Fibrils & other Multimeric Structures | 48 | 18 | 37.5 | 0.007462 | | |
| Wikipathways | Nuclear Receptors Meta-Pathway | 316 | 66 | 20.9 | 0.011199 | | |
| Wikipathways | Fatty Acid Biosynthesis | 22 | 11 | 50.0 | 0.011199 | | |
| Reactome | Neutrophil Degranulation | 490 | 92 | 19.0 | 0.012927 | | |
| PID | Integrins in Angiogenesis | 63 | 20 | 31.7 | 0.020661 | | |
| Reactome | Response to Elevated Platelet Cytosolic Ca^2+^ | 134 | 33 | 24.6 | 0.028604 | | |
| Reactome | Platelet Degranulation | 129 | 32 | 24.8% | 0.028604 | | |
