# Supplementary material for: Divergent organ-specific isogenic metastatic cell lines identified using multi-omics exhibit differential drug sensitivity
Source: PLoS One. 2020 Nov 16;15(11):e0242384. doi: 10.1371/journal.pone.0242384 (PMC7668614; doi:10.1371/journal.pone.0242384)
Supplement: S12 Table — (DOCX) [file pone.0242384.s023.docx]

| **S12** **Table. Transcriptomic-based pathway discovery for the metastatic Lymph Node-231 cell line.** | | | | | |
| --- | --- | --- | --- | --- | --- |
| **Source** | **Up Pathways** | **# of Genes in Set** | **# of Obs. Genes** | **Obs. Genes (%)** | **q-value** |
| Reactome | Extracellular Matrix Organization | 294 | 114 | 38.8% | 1.01E-12 |
| SMPDB | Simvastatin Action Pathway | 22 | 18 | 81.8% | 3.39E-08 |
| SMPDB | Hyper-IgD Syndrome | 22 | 18 | 81.8% | 3.39E-08 |
| SMPDB | Cholesteryl Ester Storage Disease | 22 | 18 | 81.8% | 3.39E-08 |
| SMPDB | Lysosomal Acid Lipase Deficiency (Wolman Disease) | 22 | 18 | 81.8% | 3.39E-08 |
| SMPDB | Mevalonic Aciduria | 22 | 18 | 81.8% | 3.39E-08 |
| SMPDB | Wolman Disease | 22 | 18 | 81.8% | 3.39E-08 |
| SMPDB | Smith-Lemli-Opitz Syndrome | 22 | 18 | 81.8% | 3.39E-08 |
| SMPDB | Chondrodysplasia Punctata II, X Linked Dominant (CDPX2) | 22 | 18 | 81.8% | 3.39E-08 |
| SMPDB | CHILD Syndrome | 22 | 18 | 81.8% | 3.39E-08 |
|  | **Down Pathways** |  |  |  |  |
| Reactome | Cell Cycle | 564 | 151 | 26.8% | 2.68E-08 |
| Wikipathways | DNA Replication | 42 | 26 | 61.9% | 2.71E-08 |
| Reactome | DNA Replication | 80 | 38 | 47.5% | 3.12E-08 |
| Reactome | Cell Cycle, Mitotic | 481 | 131 | 27.3% | 3.37E-08 |
| Reactome | S Phase | 103 | 44 | 42.7% | 3.37E-08 |
| Reactome | Synthesis of DNA | 75 | 36 | 48.0% | 3.37E-08 |
| Reactome | Activation of the Pre-Replicative Complex | 33 | 22 | 66.7% | 3.37E-08 |
| Wikipathways | Retinoblastoma Gene in Cancer | 89 | 39 | 43.8% | 1.33E-07 |
| Reactome | DNA Replication Pre-Initiation | 37 | 22 | 59.5% | 5.96E-07 |
| Reactome | M/G1 Transition | 37 | 22 | 59.5% | 5.96E-07 |
