# Supplementary material for: Divergent organ-specific isogenic metastatic cell lines identified using multi-omics exhibit differential drug sensitivity
Source: PLoS One. 2020 Nov 16;15(11):e0242384. doi: 10.1371/journal.pone.0242384 (PMC7668614; doi:10.1371/journal.pone.0242384)
Supplement: S13 Table — (DOCX) [file pone.0242384.s024.docx]

| **S13** **Table. Transcriptomic-based Unique pathways for the metastatic Brain-435 cell line.** | | | | | |
| --- | --- | --- | --- | --- | --- |
| **Source** | **Up Pathways** | **# of Genes in Set** | **# of Obs. Genes** | **Obs. Genes (%)** | **q-value** |
| Reactome | Collagen Chain Trimerization | 44 | 12 | 27.3 | 0.00151 |
| Reactome | Presynaptic Depolarization & Calcium Channel Opening | 13 | 6 | 46.2 | 0.00574 |
| Wikipathways | MAPK Signaling Pathway | 246 | 30 | 12.2 | 0.00574 |
| Reactome | NCAM1 Interactions | 37 | 9 | 24.3 | 0.01132 |
| PID | α6-β4-Integrin-ligand Interactions | 11 | 5 | 45.5 | 0.01312 |
| HumanCyc | Phenylethylamine Degradation I | 4 | 3 | 75.0 | 0.03149 |
| PharmGKB | Celecoxib Pathway, Pharmacodynamics | 58 | 10 | 17.2 | 0.04524 |
| KEGG | Protein Digestion & Absorption | 90 | 13 | 14.4 | 0.04741 |
| Reactome | Neurotoxicity of Clostridium Toxins | 10 | 4 | 40.0 | 0.05040 |
|  | **Down Pathways** |  |  |  |  |
| Reactome | Metabolism of Cofactors | 19 | 7 | 36.8 | 2.35E-05 |
| PID | Calcineurin-regulated NFAT-Dependent Transcription in Lymphocytes | 49 | 8 | 16.3 | 0.001613 |
| SMPDB | Ubiquinone Biosynthesis | 5 | 3 | 60.0 | 0.005187 |
| HumanCyc | Ubiquinol-10 Biosynthesis | 7 | 3 | 42.9 | 0.012044 |
| Reactome | Ubiquinol Biosynthesis | 8 | 3 | 37.5 | 0.017334 |
| Reactome | Tetrahydrobiopterin (BH4) Synthesis, Recycling, Salvage & Regulation | 10 | 3 | 30.0 | 0.031080 |
| Reactome | Sodium/Calcium Exchangers | 11 | 3 | 27.3 | 0.039259 |
| KEGG | Ubiquinone & Other Terpenoid-quinone Biosynthesis | 11 | 3 | 27.3 | 0.039259 |
