# Supplementary material for: Divergent organ-specific isogenic metastatic cell lines identified using multi-omics exhibit differential drug sensitivity
Source: PLoS One. 2020 Nov 16;15(11):e0242384. doi: 10.1371/journal.pone.0242384 (PMC7668614; doi:10.1371/journal.pone.0242384)
Supplement: S14 Table — (DOCX) [file pone.0242384.s025.docx]

| **S14 Table. Transcriptomic-based Unique pathways for the metastatic Liver-435 cell line.** | | | | | |
| --- | --- | --- | --- | --- | --- |
| **Source** | **Up Pathways** | **# of Genes in Set** | **# of Obs. Genes** | **Obs. Genes (%)** | **q-value** |
| Reactome | Interferon-α/β Signaling | 70 | 30 | 42.9 | 1.62E-11 |
| Wikipathways | Interferon-α/β Signaling | 21 | 15 | 71.4 | 1.69E-09 |
| KEGG | Lysosome | 123 | 35 | 28.5 | 6.68E-08 |
| Wikipathways | Type II Interferon Signaling | 37 | 15 | 40.5 | 2.04E-05 |
| Wikipathways | Human Immune Response to Tuberculosis | 22 | 11 | 50.0 | 5.32E-05 |
| Reactome | Keratan Sulfate/Keratin Metabolism | 33 | 13 | 39.4 | 0.00013 |
| Reactome | Endosomal/Vacuolar Pathway | 13 | 8 | 61.5 | 0.00019 |
| Reactome | Retinoid Metabolism & Transport | 45 | 15 | 33.3 | 0.00023 |
| Reactome | Chondroitin Sulfate/Dermatan Sulfate Metabolism | 50 | 15 | 30.0 | 0.00085 |
| BioCarta | Antigen Processing & Presentation | 12 | 7 | 58.3 | 0.00110 |
|  | **Down Pathways** |  |  |  |  |
| Reactome | Regulation of TP53 Activity through Phosphorylation | 93 | 35 | 37.6% | 4.81E-16 |
| Reactome | Transcriptional Regulation by TP53 | 374 | 70 | 18.9% | 7.60E-14 |
| Reactome | Regulation of TP53 Activity | 162 | 42 | 25.9% | 5.62E-13 |
| Reactome | Mitotic G2-G2/M phases | 139 | 34 | 24.5% | 8.48E-10 |
| Reactome | G2/M Transition | 137 | 33 | 24.1% | 2.31E-09 |
| PID | PLK1 Signaling Events | 44 | 18 | 40.9% | 3.93E-09 |
| BioCarta | Role of BRCA1, BRCA2, & ATR in Cancer Susceptibility | 22 | 13 | 59.1% | 4.47E-09 |
| Reactome | Processing of DNA Double-strand Break Ends | 99 | 27 | 27.6% | 4.47E-09 |
| PID | BARD1 Signaling Events | 29 | 14 | 48.3% | 2.85E-08 |
| Reactome | G2/M DNA Damage Checkpoint | 96 | 25 | 26.3% | 5.43E-08 |
