# Supplementary material for: Divergent organ-specific isogenic metastatic cell lines identified using multi-omics exhibit differential drug sensitivity
Source: PLoS One. 2020 Nov 16;15(11):e0242384. doi: 10.1371/journal.pone.0242384 (PMC7668614; doi:10.1371/journal.pone.0242384)
Supplement: S15 Table — (DOCX) [file pone.0242384.s026.docx]

| **S15 Table. Transcriptomic-based Unique pathways for the metastatic Lung-435 cell line.** | | | | | |  |
| --- | --- | --- | --- | --- | --- | --- |
| **Source** | **Up Pathways** | **# of Genes in Set** | **# of Obs. Genes** | **Obs. Genes (%)** | **q-value** | |
| Reactome | Metabolism of RNA | 586 | 175 | 30.0 | 4.77E-16 | |
| Reactome | rRNA Modification in the Nucleus & Cytosol | 59 | 33 | 56.9 | 2.60E-10 | |
| Reactome | rRNA Processing in the Nucleus & Cytosol | 59 | 33 | 56.9 | 2.60E-10 | |
| Reactome | rRNA Processing | 65 | 33 | 51.6 | 8.68E-09 | |
| Reactome | Cellular Responses to External Stimuli | 414 | 109 | 26.4 | 2.20E-06 | |
| Reactome | Chromatin Modifying Enzymes | 272 | 79 | 29.0 | 2.65E-06 | |
| Reactome | Chromatin Organization | 272 | 79 | 29.0 | 2.65E-06 | |
| Reactome | Cytosolic tRNA Aminoacylation | 24 | 16 | 66.7 | 6.31E-06 | |
| Reactome | HATs Acetylate Histones | 142 | 48 | 33.8 | 1.24E-05 | |
| Reactome | tRNA Processing | 103 | 37 | 35.9 | 6.84E-05 | |
|  | **Down Pathways** |  |  |  |  | |
| Reactome | O-Linked Glycosylation | 116 | 36 | 31.0 | 0.004207 | |
| Reactome | LGI-ADAM Interactions | 14 | 9 | 64.3 | 0.007771 | |
| KEGG | Calcium Signaling Pathway | 186 | 47 | 25.4 | 0.014871 | |
| Reactome | NCAM1 Interactions | 37 | 15 | 40.5 | 0.017541 | |
| Reactome | NCAM Signaling for Neurite Out-growth | 59 | 20 | 33.9 | 0.021798 | |
| Reactome | O-Glycosylation of TSR Domain-Containing Proteins | 39 | 15 | 38.5 | 0.025734 | |
| Reactome | Collagen Degradation | 35 | 14 | 40.0 | 0.025734 | |
| PID | Signaling Mediated by p38-γ/δ | 12 | 7 | 63.6 | 0.028928 | |
| Reactome | Integrin Cell Surface Interactions | 67 | 21 | 31.3 | 0.034845 | |
| Reactome | Invadopodia Formation | 4 | 4 | 100.0 | 0.034845 | |
