# Supplementary material for: Divergent organ-specific isogenic metastatic cell lines identified using multi-omics exhibit differential drug sensitivity
Source: PLoS One. 2020 Nov 16;15(11):e0242384. doi: 10.1371/journal.pone.0242384 (PMC7668614; doi:10.1371/journal.pone.0242384)
Supplement: S16 Table — (DOCX) [file pone.0242384.s027.docx]

| **S16 Table. Transcriptomic-based Unique pathways for the metastatic Spine-435 cell line.** | | | | | |
| --- | --- | --- | --- | --- | --- |
| **Source** | **Up Pathways** | **# of Genes in Set** | **# of Obs. Genes** | **Obs. Genes (%)** | **q-value** |
| Wikipathways | Striated Muscle Contraction Pathway | 38 | 14 | 36.8 | 5.61E-06 |
| Reactome | Striated Muscle Contraction | 35 | 12 | 34.3 | 6.42E-05 |
| KEGG | Val, Leu, & Ile degradation | 48 | 10 | 20.8 | 0.008216 |
| Reactome | His, Lys, Phe, Tyr, Pro, & Trp Catabolism | 49 | 10 | 20.8 | 0.008216 |
| HumanCyc | Ketogenesis | 8 | 4 | 50.0 | 0.019014 |
| INOH | Nicotinate Nicotinamide Metabolism | 24 | 6 | 26.1 | 0.026291 |
| INOH | Val, Leu, & Ile Degradation | 32 | 7 | 21.9 | 0.030188 |
| Reactome | Ion Channel Transport | 179 | 20 | 11.2 | 0.030396 |
| Wikipathways | Catalytic Cycle of Mammalian Flavin-containing Mono-Oxygenase | 5 | 3 | 60.0 | 0.032800 |
| Reactome | Melanin Biosynthesis | 5 | 3 | 60.0 | 0.032800 |
|  | **Down Pathways** |  |  |  |  |
| INOH | CD4 T Cell Receptor Signaling-JNK Cascade | 55 | 14 | 25.5 | 0.000172 |
| KEGG | HIF-1 Signaling Pathway | 100 | 18 | 18.0 | 0.000837 |
| Wikipathways | HIF-1α & PPARG Regulation of Glycolysis | 8 | 5 | 62.5 | 0.001704 |
| HumanCyc | Superpathway of Conversion of Glucose to Acetyl CoA & Entry into the TCA Cycle | 48 | 11 | 23.4 | 0.002374 |
| KEGG | Glycolysis/Gluconeogenesis | 68 | 13 | 19.1 | 0.003805 |
| SMPDB | Glycogenosis, Type VII. Tarui Disease | 15 | 6 | 40.0 | 0.003959 |
| SMPDB | Fanconi-bickel Syndrome | 15 | 6 | 40.0 | 0.003959 |
| KEGG | Phagosome | 152 | 21 | 13.9 | 0.003959 |
| KEGG | Rheumatoid Arthritis | 90 | 15 | 16.9 | 0.004380 |
| SMPDB | Glycogenosis, Type IA. Von Gierke Disease | 22 | 7 | 31.8 | 0.004380 |
