# Supplementary material for: Divergent organ-specific isogenic metastatic cell lines identified using multi-omics exhibit differential drug sensitivity
Source: PLoS One. 2020 Nov 16;15(11):e0242384. doi: 10.1371/journal.pone.0242384 (PMC7668614; doi:10.1371/journal.pone.0242384)
Supplement: S17 Table — (DOCX) [file pone.0242384.s028.docx]

| **S17 Table. Transcriptomic-based Unique pathways for the metastatic Lung-231 cell line.** | | | | | |
| --- | --- | --- | --- | --- | --- |
| **Source** | **Up Pathways** | **# of Genes in Set** | **# of Obs. Genes** | **Obs. Genes (%)** | **q-value** |
| HumanCyc | Lys Degradation II (Pipecolate Pathway) | 6 | 5 | 83.3 | 0.005380 |
| Reactome | TP53 Regulates Transcription of Death Receptors & Ligands | 12 | 7 | 58.3 | 0.005836 |
| Reactome | TCF Dependent Signaling in Response to WNT | 190 | 37 | 19.7 | 0.020855 |
| Reactome | Vitamin B1 Metabolism | 5 | 4 | 80.0 | 0.025780 |
| Reactome | Positive Epigenetic Regulation of rRNA Expression | 109 | 24 | 22.2 | 0.030405 |
| Reactome | Lys Catabolism | 12 | 6 | 50.0 | 0.034577 |
| Reactome | Signaling by WNT | 278 | 48 | 17.4 | 0.053002 |
| Reactome | Purine Salvage | 13 | 6 | 46.2 | 0.053002 |
|  | **Down Pathways** |  |  |  |  |
| Wikipathways | Nuclear Receptors Meta-Pathway | 316 | 66 | 20.9 | 0.011200 |
| Wikipathways | Fatty Acid Biosynthesis | 22 | 11 | 50.0 | 0.011200 |
| Reactome | Neutrophil Degranulation | 490 | 92 | 19.0 | 0.012926 |
| PID | Integrins in Angiogenesis | 63 | 20 | 31.7 | 0.020661 |
| Reactome | Response to Elevated Platelet Cytosolic Ca^2+^ | 134 | 33 | 24.6 | 0.028604 |
| KEGG | Lysosome | 123 | 31 | 25.2 | 0.028604 |
| Reactome | Platelet degranulation | 129 | 32 | 24.8 | 0.028604 |
| PID | β3-Integrin Cell Surface Interactions | 44 | 15 | 34.1 | 0.043613 |
| Reactome | EPH-Ephrin Signaling | 74 | 21 | 28.4 | 0.046178 |
