# Supplementary material for: Divergent organ-specific isogenic metastatic cell lines identified using multi-omics exhibit differential drug sensitivity
Source: PLoS One. 2020 Nov 16;15(11):e0242384. doi: 10.1371/journal.pone.0242384 (PMC7668614; doi:10.1371/journal.pone.0242384)
Supplement: S18 Table — (DOCX) [file pone.0242384.s029.docx]

| **S18 Table.** **Transcriptomic-based Unique pathways for the metastatic Lymph Node-231 cell line.** | | | | | |
| --- | --- | --- | --- | --- | --- |
| **Source** | **Up Pathways** | **# of Genes in Set** | **# of Obs. Genes** | **Obs. Genes (%)** | **q-value^1^** |
| Reactome | O-Glycosylation of TSR Domain-containing Proteins | 39 | 20 | 51.3 | 0.000302 |
| Reactome | Fatty Acid Metabolism | 189 | 54 | 29.2 | 0.013375 |
| PID | EPHA Forward Signaling | 33 | 15 | 45.5 | 0.015860 |
| Reactome | Activation of Ca-permeable Kainate Receptor | 10 | 7 | 70.0 | 0.020852 |
| Reactome | Ionotropic Activity of Kainate Receptors | 10 | 7 | 70.0 | 0.020852 |
| KEGG | Parathyroid Hormone Synthesis, Secretion, & Action | 106 | 34 | 32.1 | 0.023528 |
| Reactome | Linoleic Acid Metabolism | 8 | 6 | 75.0 | 0.028261 |
| Wikipathways | Non-genomic Actions of 1,25-Dihydroxyvitamin D3 | 68 | 24 | 35.3 | 0.028880 |
| Reactome | Regulation of TLR by Endogenous Ligand | 16 | 9 | 56.2 | 0.028894 |
| PID | Osteopontin-mediated Events | 32 | 14 | 43.8 | 0.030820 |
|  | **Down Pathways** |  |  |  |  |
| Reactome | E2F-Enabled Inhibition of Pre-Replication Complex Formation | 9 | 7 | 77.8 | 0.004146 |
| Reactome | CLEC7A (Dectin-1) Signaling | 40 | 15 | 37.5 | 0.028510 |
| Reactome | C-Type Lectin Receptors (CLRs) | 83 | 25 | 30.1 | 0.030374 |
| EHMN | Vitamin E Metabolism | 43 | 15 | 34.9 | 0.054502 |
| SMPDB | Familial Lipoprotein Lipase Deficiency | 13 | 7 | 53.8 | 0.054502 |
| SMPDB | Glycerolipid Metabolism | 13 | 7 | 53.8 | 0.054502 |
| SMPDB | Glycerol Kinase Deficiency | 13 | 7 | 53.8 | 0.054502 |
| SMPDB | D-Glyceric Acidura | 13 | 7 | 53.8 | 0.054502 |
| **^1^**Gray shading of values indicates that the pathways are trending to significance. | | | | | |
