# Supplementary material for: Divergent organ-specific isogenic metastatic cell lines identified using multi-omics exhibit differential drug sensitivity
Source: PLoS One. 2020 Nov 16;15(11):e0242384. doi: 10.1371/journal.pone.0242384 (PMC7668614; doi:10.1371/journal.pone.0242384)
Supplement: S19 Table — (DOCX) [file pone.0242384.s030.docx]

| **S19 Table. Common proteome and transcriptome pathways for the metastatic Brain-435 cell line.** | | | | | |
| --- | --- | --- | --- | --- | --- |
| **Source** | **Up Pathways** | **# of Genes in Set** | **# of Obs. Genes** | **Obs. Genes (%)** | **q-value** |
| Reactome | Developmental Biology | 620 | 6 | 1.0 | 0.020124 |
| Wikipathways | MAPK Signaling Pathway | 246 | 4 | 1.6 | 0.020124 |
| KEGG | MAPK signaling pathway | 295 | 4 | 1.4 | 0.020124 |
| Reactome | Integrin Cell Surface Interactions | 67 | 3 | 4.5 | 0.020124 |
| KEGG | C-Type Lectin Receptor Signaling Pathway | 104 | 3 | 2.9 | 0.020124 |
| KEGG | Apelin Signaling Pathway | 137 | 3 | 2.2 | 0.020124 |
| KEGG | Phospholipase D Signaling Pathway | 146 | 3 | 2.1 | 0.020124 |
| Wikipathways | Regulation of Actin Cytoskeleton | 151 | 3 | 2.0 | 0.020124 |
| Wikipathways | Inhibition of Exosome Biogenesis & Secretion by Manumycin A in CRPC Cells | 18 | 2 | 11.1 | 0.020124 |
| PID | Plexin-D1 Signaling | 24 | 2 | 8.3 | 0.020124 |
|  | **Down Pathways** |  |  |  |  |
| Reactome | Cell Cycle | 564 | 20 | 3.6 | 8.28E-11 |
| Reactome | Cell Cycle, Mitotic | 481 | 18 | 3.8 | 2.16E-10 |
| Wikipathways | Mitotic G1-G1-S phases | 20 | 7 | 35.0 | 2.16E-10 |
| Wikipathways | DNA Replication | 42 | 7 | 16.7 | 3.74E-08 |
| Reactome | Mitotic G1-G1/S Phases | 104 | 9 | 8.7 | 4.17E-08 |
| Reactome | G1/S Transition | 76 | 8 | 10.5 | 6.44E-08 |
| Wikipathways | Retinoblastoma Gene in Cancer | 89 | 8 | 9.0 | 1.87E-07 |
| Reactome | Activation of the Pre-replicative Complex | 33 | 6 | 18.2 | 1.87E-07 |
| Reactome | Activation of E2F1 Target Genes at G1/S | 17 | 5 | 29.4 | 2.05E-07 |
| Reactome | G1/S-Specific Transcription | 17 | 5 | 29.4 | 2.05E-07 |
