# Supplementary material for: Divergent organ-specific isogenic metastatic cell lines identified using multi-omics exhibit differential drug sensitivity
Source: PLoS One. 2020 Nov 16;15(11):e0242384. doi: 10.1371/journal.pone.0242384 (PMC7668614; doi:10.1371/journal.pone.0242384)
Supplement: S20 Table — (DOCX) [file pone.0242384.s031.docx]

| **S20 Table.** **Common proteome and transcriptome pathways for the metastatic Liver-435 cell line.** | | | | | |
| --- | --- | --- | --- | --- | --- |
| **Source** | **Up Pathways** | **# of Genes in Set** | **# of Obs. Genes** | **Obs. Genes (%)** | **q-value** |
| KEGG | Lysosome | 123 | 9 | 7.3 | 2.51E-08 |
| Reactome | Neutrophil Degranulation | 490 | 11 | 2.3 | 2.11E-05 |
| Reactome | Innate Immune System | 1077 | 13 | 1.2 | 0.001011 |
| Reactome | MHC Class II Antigen Presentation | 59 | 4 | 6.8 | 0.001519 |
| KEGG | Antigen Processing & Presentation | 77 | 4 | 5.2 | 0.003464 |
| Reactome | Hemostasis | 668 | 9 | 1.3 | 0.006010 |
| Reactome | Immune System | 1840 | 15 | 0.8 | 0.008389 |
| Reactome | Metabolism of Fat-soluble Vitamins | 49 | 3 | 6.1 | 0.010501 |
| Wikipathways | Ebola Virus Pathway on Host | 130 | 4 | 3.1 | 0.011678 |
| BioCarta | Antigen Processing & Presentation | 12 | 2 | 16.7 | 0.011678 |
|  | **Down Pathways** |  |  |  |  |
| Reactome | Cell Cycle | 564 | 13 | 2.3 | 7.58E-06 |
| Wikipathways | Retinoblastoma Gene in Cancer | 89 | 6 | 6.7 | 5.50E-05 |
| Reactome | Cell Cycle, Mitotic | 481 | 10 | 2.1 | 0.000223 |
| Reactome | Termination of Translesion DNA Synthesis | 32 | 4 | 12.5 | 0.000223 |
| Reactome | Translesion Synthesis by Y Family DNA Polymerases Bypasses Lesions on DNA Template | 39 | 4 | 10.3 | 0.000347 |
| Wikipathways | DNA Replication | 42 | 4 | 9.5 | 0.000391 |
| Reactome | Cell Cycle Checkpoints | 250 | 7 | 2.8 | 0.000545 |
| Reactome | DNA Damage Bypass | 49 | 4 | 8.2 | 0.000545 |
| Reactome | S Phase | 103 | 5 | 4.9 | 0.000561 |
| Reactome | Chromosome Maintenance | 112 | 5 | 4.5 | 0.000693 |
