# Supplementary material for: Divergent organ-specific isogenic metastatic cell lines identified using multi-omics exhibit differential drug sensitivity
Source: PLoS One. 2020 Nov 16;15(11):e0242384. doi: 10.1371/journal.pone.0242384 (PMC7668614; doi:10.1371/journal.pone.0242384)
Supplement: S21 Table — (DOCX) [file pone.0242384.s032.docx]

| **S21 Table. Common proteome and transcriptome pathways for the metastatic Lung-435 cell line.** | | | | | |
| --- | --- | --- | --- | --- | --- |
| **Source** | **Up Pathways** | **# of Genes in Set** | **# of Obs. Genes** | **Obs. Genes (%)** | **q-value^1^** |
| Wikipathways | miR-targeted genes in muscle cell - TarBase | 400 | 12 | 3.0 | 0.055336 |
| KEGG | Herpes simplex infection - | 185 | 8 | 4.3 | 0.055336 |
| Reactome | rRNA modification in the nucleus and cytosol | 59 | 5 | 8.6 | 0.055336 |
| Reactome | rRNA processing in the nucleus and cytosol | 59 | 5 | 8.6 | 0.055336 |
| Reactome | rRNA processing | 65 | 5 | 7.8 | 0.055336 |
|  | **Down Pathways** |  |  |  |  |
| Reactome | Collagen Formation | 92 | 9 | 9.8 | 0.000118 |
| Reactome | Extracellular Matrix Organization | 294 | 14 | 4.8 | 0.000260 |
| HumanCyc | Glycolysis | 25 | 5 | 20.8 | 0.000395 |
| Reactome | Assembly of Collagen Fibrils & Other Multimeric Structures | 48 | 6 | 12.5 | 0.000684 |
| Reactome | Post-translational Protein Phosphorylation | 110 | 8 | 7.3 | 0.000940 |
| Wikipathways | Photodynamic Therapy-induced HIF-1 Survival Signaling | 36 | 5 | 13.9 | 0.001590 |
| Reactome | Regulation of IGF Transport & uptake by IGFBPs | 127 | 8 | 6.3 | 0.001923 |
| Reactome | Collagen Biosynthesis & Modifying Enzymes | 68 | 6 | 8.8 | 0.002588 |
| Reactome | Collagen Chain Trimerization | 44 | 5 | 11.4 | 0.002614 |
| INOH | Glycolysis Gluconeogenesis | 46 | 5 | 11.1 | 0.002614 |
| **^1^**Gray shading of values indicates that the pathways are trending to significance. | | | | | |
