# Supplementary material for: Divergent organ-specific isogenic metastatic cell lines identified using multi-omics exhibit differential drug sensitivity
Source: PLoS One. 2020 Nov 16;15(11):e0242384. doi: 10.1371/journal.pone.0242384 (PMC7668614; doi:10.1371/journal.pone.0242384)
Supplement: S22 Table — (DOCX) [file pone.0242384.s033.docx]

| **S22 Table.** **Common proteome and transcriptome pathways for the metastatic Spine-435 cell line.** | | | | | |
| --- | --- | --- | --- | --- | --- |
| **Source** | **Up Pathways** | **# of Genes in Set** | **# of Obs. Genes** | **Obs. Genes (%)** | **q-value** |
| Wikipathways | Cholesterol Biosynthesis, Regulation & Transport | 9 | 3 | 33.3 | 2.64E-05 |
| SMPDB | Simvastatin Action Pathway | 22 | 3 | 13.6 | 2.64E-05 |
| SMPDB | Hyper-IgD Syndrome | 22 | 3 | 13.6 | 2.64E-05 |
| SMPDB | Cholesteryl Ester Storage Disease | 22 | 3 | 13.6 | 2.64E-05 |
| SMPDB | Lysosomal Acid Lipase Deficiency (Wolman Disease) | 22 | 3 | 13.6 | 2.64E-05 |
| SMPDB | Mevalonic Aciduria | 22 | 3 | 13.6 | 2.64E-05 |
| SMPDB | Wolman Disease | 22 | 3 | 13.6 | 2.64E-05 |
| SMPDB | Smith-Lemli-Opitz Syndrome | 22 | 3 | 13.6 | 2.64E-05 |
| SMPDB | Chondrodysplasia Punctata II, X Linked Dominant (CDPX2) | 22 | 3 | 13.6 | 2.64E-05 |
| SMPDB | CHILD Syndrome | 22 | 3 | 13.6 | 2.64E-05 |
|  | **Down Pathways** |  |  |  |  |
| HumanCyc | Glycolysis | 25 | 8 | 33.3 | 5.61E-11 |
| Wikipathways | Glycolysis & Gluconeogenesis | 45 | 9 | 20.0 | 1.46E-10 |
| Wikipathways | Glycolysis Pathway D (2) | 23 | 7 | 30.4 | 1.31E-09 |
| KEGG | Glycolysis/Gluconeogenesis | 68 | 9 | 13.2 | 3.01E-09 |
| INOH | Glycolysis Gluconeogenesis | 46 | 8 | 17.8 | 3.01E-09 |
| HumanCyc | Superpathway of Conversion of Glucose to Acetyl CoA Entry into the TCA Cycle | 48 | 8 | 17.0 | 3.63E-09 |
| Wikipathways | Pathways in Clear Cell Renal Cell Carcinoma | 86 | 9 | 10.5 | 1.82E-08 |
| PID | HIF-1α Transcription Factor Network | 66 | 8 | 12.1 | 4.59E-08 |
| Reactome | Glycolysis | 71 | 8 | 11.4 | 5.94E-08 |
| HumanCyc | Gluconeogenesis | 26 | 6 | 24.0 | 5.94E-08 |
