# Supplementary material for: Divergent organ-specific isogenic metastatic cell lines identified using multi-omics exhibit differential drug sensitivity
Source: PLoS One. 2020 Nov 16;15(11):e0242384. doi: 10.1371/journal.pone.0242384 (PMC7668614; doi:10.1371/journal.pone.0242384)
Supplement: S23 Table — (DOCX) [file pone.0242384.s034.docx]

| **S23 Table. Common proteome and transcriptome pathways for the metastatic Lung-231 cell line.** | | | | | |
| --- | --- | --- | --- | --- | --- |
| **Source** | **Up Pathways** | **# of Genes in Set** | **# of Obs. Genes** | **Obs. Genes (%)** | **q-value** |
| Reactome | Signaling by Rho GTPases | 435 | 21 | 4.9 | 0.000603 |
| Reactome | Vitamin B5 Metabolism | 14 | 4 | 28.6 | 0.010842 |
| Reactome | EPHA-mediated Growth Cone Collapse | 15 | 4 | 26.7 | 0.010842 |
| Reactome | RHO GTPase Effectors | 301 | 13 | 4.3 | 0.017389 |
| KEGG | Tight Junction | 170 | 10 | 5.9 | 0.017389 |
| Reactome | Cellular Senescence | 189 | 10 | 5.3 | 0.017389 |
| Wikipathways | Regulation of Actin Cytoskeleton | 151 | 9 | 6.0 | 0.017389 |
| Reactome | Metabolism of Nucleotides | 105 | 8 | 7.6 | 0.017389 |
| PID | β3-Integrin Cell Surface Interactions | 44 | 5 | 11.4 | 0.017389 |
| KEGG | Pantothenate & CoA Biosynthesis | 19 | 4 | 21.1 | 0.017389 |
|  | **Down Pathways** |  |  |  |  |
| Reactome | Neutrophil Degranulation | 490 | 40 | 8.2 | 3.14E-07 |
| NetPath | EGFR1 | 457 | 37 | 8.1 | 1.15E-06 |
| Reactome | Metabolism of Carbohydrates | 264 | 26 | 9.9 | 4.36E-06 |
| KEGG | Lysosome | 123 | 16 | 13.0 | 5.79E-05 |
| Reactome | Innate Immune System | 1077 | 57 | 5.4 | 9.22E-05 |
| Reactome | Metabolism | 1972 | 87 | 4.4 | 0.000182 |
| Wikipathways | miR-targeted Genes in Muscle Cell - TarBase | 400 | 29 | 7.2 | 0.000195 |
| Wikipathways | Fatty Acid Biosynthesis | 22 | 7 | 31.8 | 0.000195 |
| Reactome | Clathrin Derived Vesicle Budding | 72 | 11 | 15.3 | 0.000395 |
| Reactome | Trans-Golgi Network Vesicle Budding | 72 | 11 | 15.3 | 0.000395 |
