# Supplementary material for: Divergent organ-specific isogenic metastatic cell lines identified using multi-omics exhibit differential drug sensitivity
Source: PLoS One. 2020 Nov 16;15(11):e0242384. doi: 10.1371/journal.pone.0242384 (PMC7668614; doi:10.1371/journal.pone.0242384)
Supplement: S24 Table — (DOCX) [file pone.0242384.s035.docx]

| **S24 Table. Common proteome and transcriptome pathways for the metastatic Lymph Node-231 cell line.** | | | | | |
| --- | --- | --- | --- | --- | --- |
| **Source** | **Up Pathways** | **# of Genes in Set** | **# of Obs. Genes** | **Obs. Genes (%)** | **q-value** |
| KEGG | Tight Junction | 170 | 14 | 8.2 | 0.001838 |
| Reactome | EPHA-mediated Growth Cone Collapse | 15 | 5 | 33.3 | 0.001933 |
| Reactome | Sema4D Induced Cell Migration & Growth-Cone Collapse | 20 | 5 | 25.0 | 0.006185 |
| Reactome | Signaling by Rho GTPases | 435 | 21 | 4.9 | 0.006316 |
| Reactome | EPH-Ephrin Signaling | 74 | 8 | 10.8 | 0.007998 |
| Reactome | Sema4D in Semaphorin Signaling | 24 | 5 | 20.8 | 0.007998 |
| KEGG | Platelet Activation - | 123 | 10 | 8.1 | 0.008556 |
| PID | Regulation of RhoA activity | 48 | 6 | 13.0 | 0.016773 |
| NetPath | EGFR1 | 457 | 20 | 4.4 | 0.017595 |
| Reactome | Rho GTPase Cycle | 144 | 10 | 7.1 | 0.017595 |
|  | **Down Pathways** |  |  |  |  |
| Reactome | Metabolism | 1972 | 111 | 5.7 | 6.74E-07 |
| Reactome | Metabolism of Carbohydrates | 264 | 27 | 10.3 | 6.77E-05 |
| Reactome | Membrane Trafficking | 582 | 42 | 7.2 | 0.000344 |
| Reactome | Vesicle-mediated Transport | 620 | 43 | 6.9 | 0.000362 |
| NetPath | EGFR1 | 457 | 35 | 7.7 | 0.000362 |
| INOH | Glycolysis Gluconeogenesis | 46 | 10 | 22.2 | 0.000362 |
| EHMN | Mono-Unsaturated Fatty Acid β-oxidation | 21 | 7 | 33.3 | 0.000507 |
| PID | PDGFR-β Signaling Pathway | 127 | 16 | 12.6 | 0.000510 |
| HumanCyc | Glycolysis | 25 | 7 | 29.2 | 0.001080 |
| Reactome | Gluconeogenesis | 35 | 8 | 23.5 | 0.001194 |
