# Supplementary material for: Divergent organ-specific isogenic metastatic cell lines identified using multi-omics exhibit differential drug sensitivity
Source: PLoS One. 2020 Nov 16;15(11):e0242384. doi: 10.1371/journal.pone.0242384 (PMC7668614; doi:10.1371/journal.pone.0242384)
Supplement: S25 Table — (DOCX) [file pone.0242384.s036.docx]

| **S25 Table. Metabolomic-based pathway discovery for the metastatic Brain-435 cell line.** | | | | | |
| --- | --- | --- | --- | --- | --- |
| **Source** | **Up Pathways** | **# of Meta-bolites in Set** | **# of Obs. Meta-**  **bolites** | **Obs. Meta-**  **bolites (%)** | **q-value** |
| Wikipathways | Biochemical Pathways Part I | 467 | 8 | 1.8 | 0.032886 |
| Wikipathways | Monoamine Transport | 14 | 2 | 15.4 | 0.033343 |
| KEGG | Pyrimidine Metabolism | 66 | 3 | 5.7 | 0.033343 |
| INOH | Arginine Proline Metabolism | 68 | 3 | 5.4 | 0.033343 |
| Wikipathways | Biogenic Amine Synthesis | 17 | 2 | 11.8 | 0.033343 |
| Reactome | Transport of Nucleosides & Free Purine & Pyrimidine Bases Across the Plasma Membrane | 18 | 2 | 11.1 | 0.033343 |
| HumanCyc | Noradrenaline & Adrenaline Degradation | 20 | 2 | 10.5 | 0.033343 |
| Reactome | Metabolism of Amino Acids & Derivatives | 285 | 5 | 2.2 | 0.033343 |
| Reactome | Pyrimidine Salvage | 23 | 2 | 8.7 | 0.042579 |
| KEGG | Val, Leu, & Ile Degradation | 42 | 2 | 7.7 | 0.043016 |
|  | **Down Pathways** |  |  |  |  |
| Wikipathways | Biochemical Pathways Part I | 467 | 55 | 12.6 | 2.41E-14 |
| Reactome | Metabolism of Carbohydrates | 137 | 26 | 26.8 | 4.38E-13 |
| Reactome | Metabolism of Nucleotides | 152 | 28 | 22.4 | 2.99E-12 |
| Reactome | Nucleobase Catabolism | 100 | 22 | 27.2 | 3.89E-11 |
| Reactome | Metabolism | 1384 | 71 | 8.2 | 1.43E-10 |
| Wikipathways | Nucleobase Catabolism | 100 | 20 | 25.6 | 1.29E-09 |
| EHMN | Pyrimidine Metabolism | 77 | 19 | 26.8 | 1.75E-09 |
| SMPDB | Pyrimidine Metabolism | 57 | 16 | 28.6 | 1.82E-08 |
| SMPDB | UMP Synthase Deiciency (Orotic Aciduria) | 57 | 16 | 28.6 | 1.82E-08 |
| SMPDB | MNGIE (MIT Neurogastro- intestinal Encephalopathy) | 57 | 16 | 28.6 | 1.82E-08 |
