# Supplementary material for: Divergent organ-specific isogenic metastatic cell lines identified using multi-omics exhibit differential drug sensitivity
Source: PLoS One. 2020 Nov 16;15(11):e0242384. doi: 10.1371/journal.pone.0242384 (PMC7668614; doi:10.1371/journal.pone.0242384)
Supplement: S26 Table — (DOCX) [file pone.0242384.s037.docx]

| **S26 Table. Metabolomic-based pathway discovery for the metastatic Liver-435 cell line.** | | | | | |
| --- | --- | --- | --- | --- | --- |
| **Source** | **Up Pathways** | **# of Meta-bolites in Set** | **# of Obs. Meta-**  **bolites** | **Obs. Meta-**  **bolites (%)** | **q-value** |
| Wikipathways | Cholesterol Biosynthesis Pathway | 14 | 3 | 21.4 | 0.019043 |
| HumanCyc | Mevalonate Pathway | 17 | 3 | 20.0 | 0.019043 |
| INOH | Tyrosine Metabolism | 44 | 4 | 9.1 | 0.019043 |
| HumanCyc | Superpathway of Geranyl- geranyldiphosphate biosynthesis I (*via* mevalonate) | 21 | 3 | 15.8 | 0.019043 |
| KEGG | Terpenoid Backbone Biosynthesis | 45 | 3 | 15.0 | 0.019043 |
| HumanCyc | Trans, Trans-Farnesyl Diphosphate Biosynthesis | 5 | 2 | 40.0 | 0.019043 |
| EHMN | Tyrosine Metabolism | 105 | 5 | 5.3 | 0.021992 |
| INOH | Steroids Metabolism | 31 | 3 | 10.7 | 0.021992 |
| KEGG | Tyrosine Metabolism | 78 | 4 | 6.3 | 0.021992 |
| Reactome | Metabolism | 1384 | 15 | 1.7 | 0.021992 |
|  | **Down Pathways** |  |  |  |  |
| Wikipathways | Biochemical Pathways Part I | 467 | 67 | 15.3 | 1.91E-22 |
| Reactome | Metabolism of Nucleotides | 152 | 35 | 28.0 | 2.88E-18 |
| Reactome | Metabolism of Carbohydrates | 137 | 30 | 30.9 | 9.46E-17 |
| Reactome | Metabolism | 1384 | 80 | 9.3 | 4.03E-14 |
| EHMN | Pyrimidine Metabolism | 77 | 24 | 33.8 | 4.03E-14 |
| Reactome | Nucleobase Catabolism | 100 | 25 | 30.9 | 8.47E-14 |
| Wikipathways | Nucleobase Catabolism | 100 | 24 | 30.8 | 3.39E-13 |
| SMPDB | Warburg Effect | 58 | 20 | 39.2 | 4.19E-13 |
| Reactome | Nucleotide Salvage | 45 | 17 | 38.6 | 8.05E-11 |
| EHMN | Glycolysis & Gluconeogenesis | 52 | 16 | 41.0 | 1.12E-10 |
