# Supplementary material for: Divergent organ-specific isogenic metastatic cell lines identified using multi-omics exhibit differential drug sensitivity
Source: PLoS One. 2020 Nov 16;15(11):e0242384. doi: 10.1371/journal.pone.0242384 (PMC7668614; doi:10.1371/journal.pone.0242384)
Supplement: S27 Table — (DOCX) [file pone.0242384.s038.docx]

| **S27 Table. Metabolomic-based pathway discovery for the metastatic Lung-435 cell line.** | | | | | |
| --- | --- | --- | --- | --- | --- |
| **Source** | **Up Pathways** | **# of Meta-**  **bolites in Set** | **# of Obs. Meta-**  **bolites** | **Obs. Meta-**  **bolites (%)** | **q-value** |
| Wikipathways | Biochemical Pathways Part I | 467 | 17 | 3.9 | 2.97E-05 |
| Reactome | Metabolism | 1384 | 21 | 2.4 | 0.000427 |
| SMPDB | Pyrimidine Metabolism | 57 | 6 | 10.7 | 0.000427 |
| SMPDB | UMP Synthase Deiciency (Orotic Aciduria) | 57 | 6 | 10.7 | 0.000427 |
| SMPDB | MNGIE (MIT Neurogastro- intestinal Encephalopathy) | 57 | 6 | 10.7 | 0.000427 |
| SMPDB | β-Ureidopropionase Deficiency | 57 | 6 | 10.7 | 0.000427 |
| SMPDB | Dihydropyrimidinase Deficiency | 57 | 6 | 10.7 | 0.000427 |
| Reactome | Metabolism of nucleotides | 152 | 7 | 5.6 | 0.004745 |
| Reactome | Transport of nucleotide sugars | 13 | 3 | 23.1 | 0.005356 |
| Wikipathways | Pyrimidine metabolism | 40 | 4 | 11.1 | 0.007892 |
|  | **Down Pathways** |  |  |  |  |
| Wikipathways | Biochemical Pathways Part I | 467 | 57 | 13.0 | 1.76E-16 |
| Reactome | Metabolism of Carbohydrates | 137 | 24 | 24.7 | 2.46E-11 |
| SMPDB | Warburg Effect | 58 | 17 | 33.3 | 6.05E-10 |
| EHMN | Pentose Phosphate Pathway | 37 | 13 | 43.3 | 3.85E-09 |
| Reactome | Metabolism of Nucleotides | 152 | 24 | 19.2 | 3.85E-09 |
| SMPDB | Pentose Phosphate Pathway | 29 | 12 | 46.2 | 5.32E-09 |
| SMPDB | Glucose-6-phosphate Dehydrogenase Deficiency | 29 | 12 | 46.2 | 5.32E-09 |
| SMPDB | Ribose-5-phosphate Isomerase Deficiency | 29 | 12 | 46.2 | 5.32E-09 |
| SMPDB | Transaldolase Deficiency | 29 | 12 | 46.2 | 5.32E-09 |
| Reactome | Nucleobase Catabolism | 100 | 19 | 23.5 | 8.46E-09 |
