# Supplementary material for: Divergent organ-specific isogenic metastatic cell lines identified using multi-omics exhibit differential drug sensitivity
Source: PLoS One. 2020 Nov 16;15(11):e0242384. doi: 10.1371/journal.pone.0242384 (PMC7668614; doi:10.1371/journal.pone.0242384)
Supplement: S28 Table — (DOCX) [file pone.0242384.s039.docx]

| **S28 Table. Metabolomic-based pathway discovery for the metastatic Spine-435 cell line.** | | | | | |
| --- | --- | --- | --- | --- | --- |
| **Source** | **Up Pathways** | **# of Meta-**  **bolites in Set** | **# of Obs. Meta-**  **bolites** | **Obs. Meta-**  **bolites (%)** | **q-value** |
| Reactome | Na+/Cl- Dependent Neurotransmitter Transporters | 31 | 6 | 21.4 | 0.000275 |
| Reactome | Amine Compound SLC Transporters | 35 | 6 | 19.4 | 0.000275 |
| EHMN | Urea Cycle & Metabolism of Arg, Pro, Glu, Asp & Asn | 125 | 9 | 9.9 | 0.000275 |
| Reactome | SLC-mediated Transmembrane Transport | 166 | 11 | 7.2 | 0.000325 |
| Wikipathways | Biochemical Pathways Part I | 467 | 18 | 4.1 | 0.000519 |
| Reactome | Amino acid & Oligopeptide SLC Transporters | 50 | 6 | 14.3 | 0.000845 |
| EHMN | Tyrosine metabolism | 105 | 8 | 8.5 | 0.001222 |
| Reactome | Transport of Inorganic Cations/Anions & Amino Acids/Oligopeptides | 56 | 6 | 12.5 | 0.001222 |
| Reactome | Metabolism of Amino Acids & Derivatives | 285 | 12 | 5.3 | 0.001222 |
| Reactome | Transport of Small Molecules | 226 | 11 | 5.7 | 0.001222 |
|  | **Down Pathways** |  |  |  |  |
| Wikipathways | Biochemical Pathways Part I | 467 | 66 | 15.1 | 4.14E-23 |
| Reactome | Metabolism of Carbohydrates | 137 | 32 | 33.0 | 1.17E-19 |
| SMPDB | Warburg Effect | 58 | 22 | 43.1 | 7.86E-16 |
| Reactome | Metabolism of Nucleotides | 152 | 29 | 23.2 | 1.87E-13 |
| SMPDB | Gluconeogenesis | 34 | 16 | 53.3 | 1.87E-13 |
| SMPDB | Glycogenosis, Type IA. Von Gierke Disease | 34 | 16 | 53.3 | 1.87E-13 |
| SMPDB | Glycogenosis, Type IC | 34 | 16 | 53.3 | 1.87E-13 |
| SMPDB | Glycogen Storage Disease Type 1A or Von Gierke Disease | 34 | 16 | 53.3 | 1.87E-13 |
| SMPDB | Triosephosphate Isomerase | 34 | 16 | 53.3 | 1.87E-13 |
| SMPDB | Fructose-1,6-Diphosphatase Deficiency | 34 | 16 | 53.3 | 1.87E-13 |
