# Supplementary material for: Divergent organ-specific isogenic metastatic cell lines identified using multi-omics exhibit differential drug sensitivity
Source: PLoS One. 2020 Nov 16;15(11):e0242384. doi: 10.1371/journal.pone.0242384 (PMC7668614; doi:10.1371/journal.pone.0242384)
Supplement: S29 Table — (DOCX) [file pone.0242384.s040.docx]

| **S29 Table. Metabolomic-based Unique pathways for the metastatic Brain-435 cell line.** | | | | | |
| --- | --- | --- | --- | --- | --- |
| **Source** | **Up Pathways** | **# of Meta-**  **bolites in Set** | **# of Obs. Meta-bolites** | **Obs. Meta-**  **bolites (%)** | **q-value** |
| Wikipathways | Monoamine Transport | 14 | 2 | 15.4 | 0.033343 |
| Reactome | Transport of Nucleosides & Free Purine & Pyrimidine Bases Across the Plasma Membrane | 18 | 2 | 11.1 | 0.033343 |
| HumanCyc | Noradrenaline & Adrenaline Degradation | 20 | 2 | 10.5 | 0.033343 |
| Reactome | Pyrimidine Salvage | 23 | 2 | 8.7 | 0.042579 |
| KEGG | Val, Leu, & Ile Degradation | 42 | 2 | 7.7 | 0.043016 |
| KEGG | cAMP Signaling Pathway | 40 | 2 | 6.7 | 0.043016 |
|  | **Down Pathways** |  |  |  |  |
| Wikipathways | mRNA, Protein, & Metabolite Inducation Pathway by Cyclosporin A | 13 | 4 | 30.8 | 0.005064 |
| HumanCyc | L-dopachrome biosynthesis | 8 | 3 | 42.9 | 0.006755 |
| Wikipathways | Arylamine Metabolism | 4 | 2 | 66.7 | 0.012948 |
| Reactome | Metabolism of Ingested SeMet, Sec, MeSec into H2Se | 32 | 5 | 17.2 | 0.013717 |
| KEGG | Phenylalanine metabolism | 72 | 7 | 12.7 | 0.014637 |
| Reactome | Reactions Specific to the Complex N-Glycan Synthesis Pathway | 19 | 3 | 27.3 | 0.018665 |
| Wikipathways | Glucose Homeostasis | 21 | 4 | 19.0 | 0.018665 |
| HumanCyc | Protein O-N-Acetyl-Glucosylation | 5 | 2 | 50.0 | 0.018665 |
| Reactome | N-Glycan Trimming & Elongation in the Cis-Golgi | 13 | 2 | 50.0 | 0.018665 |
| KEGG | Glycosylphosphatidylinositol (GPI)-Anchor Biosynthesis | 16 | 2 | 50.0 | 0.018665 |
