# Supplementary material for: Divergent organ-specific isogenic metastatic cell lines identified using multi-omics exhibit differential drug sensitivity
Source: PLoS One. 2020 Nov 16;15(11):e0242384. doi: 10.1371/journal.pone.0242384 (PMC7668614; doi:10.1371/journal.pone.0242384)
Supplement: S30 Table — (DOCX) [file pone.0242384.s041.docx]

| **S30 Table.** **Metabolomic-based Unique pathways for the metastatic Liver-435 cell line.** | | | | | |
| --- | --- | --- | --- | --- | --- |
| **Source** | **Up Pathways** | **# of Meta-**  **bolites in Set** | **# of Obs. Meta-bolites** | **Obs. Meta-**  **bolites (%)** | **q-value** |
| Wikipathways | Cholesterol Biosynthesis Pathway | 14 | 3 | 21.4 | 0.019043 |
| HumanCyc | Mevalonate pathway | 17 | 3 | 20.0 | 0.019043 |
| HumanCyc | Superpathway of Geranyl- geranyldiphosphate Biosynthesis I (*via* Mevalonate) | 21 | 3 | 15.8 | 0.019043 |
| KEGG | Terpenoid Backbone Biosynthesis | 45 | 3 | 15.0 | 0.019043 |
| INOH | Steroids Metabolism | 31 | 3 | 10.7 | 0.021992 |
| Wikipathways | Cholesterol Biosynthesis, Regulation & Transport | 11 | 2 | 20.0 | 0.021992 |
| KEGG | Regulation of Lipolysis in Adipocytes | 15 | 2 | 18.2 | 0.021992 |
| EHMN | Squalene & Cholesterol Biosynthesis | 42 | 3 | 8.1 | 0.021992 |
| HumanCyc | Superpathway of Cholesterol Biosynthesis | 62 | 3 | 8.1 | 0.021992 |
| SMPDB | Hyper-IgD Syndrome | 45 | 3 | 7.3 | 0.021992 |
|  | **Down Pathways** |  |  |  |  |
| SMPDB | Hyperornithinemia with Gyrate Atrophy (HOGA) | 52 | 11 | 22.4 | 3.23E-05 |
| SMPDB | Creatine Deficiency, Guanidinoacetate Methyltransferase Deficiency | 52 | 11 | 22.4 | 3.23E-05 |
| SMPDB | L-Arg:Gly Amidinotransferase Deficiency | 52 | 11 | 22.4 | 3.23E-05 |
| SMPDB | Hyperornithinemia-Hyper- ammonemia-Homocitrullinuria – HHH-syndrome | 52 | 11 | 22.4 | 3.23E-05 |
| SMPDB | Guanidinoacetate Methyl- transferase Deficiency | 52 | 11 | 22.4 | 3.23E-05 |
| SMPDB | Prolinemia Type II | 52 | 11 | 22.4 | 3.23E-05 |
| SMPDB | Prolidase Deficiency (PD) | 52 | 11 | 22.4 | 3.23E-05 |
| SMPDB | Arg & Pro Metabolism | 52 | 11 | 22.4 | 3.23E-05 |
| SMPDB | Hyperprolinemia Type I | 52 | 11 | 22.4 | 3.23E-05 |
| SMPDB | Hyperprolinemia Type II | 52 | 11 | 22.4 | 3.23E-05 |
