# Supplementary material for: Divergent organ-specific isogenic metastatic cell lines identified using multi-omics exhibit differential drug sensitivity
Source: PLoS One. 2020 Nov 16;15(11):e0242384. doi: 10.1371/journal.pone.0242384 (PMC7668614; doi:10.1371/journal.pone.0242384)
Supplement: S31 Table — (DOCX) [file pone.0242384.s042.docx]

| **S31 Table.** **Metabolomic-based Unique pathways for the metastatic Liver-435 cell line.** | | | | | |
| --- | --- | --- | --- | --- | --- |
| **Source** | **Up Pathways** | **# of Meta-**  **bolites in Set** | **# of Obs. Meta-bolites** | **Obs. Meta-**  **bolites (%)** | **q-value** |
| SMPDB | UMP Synthase Deiciency (Orotic Aciduria) | 57 | 6 | 10.7 | 0.000427 |
| SMPDB | MNGIE (MIT Neurogastro- intestinal Encephalopathy) | 57 | 6 | 10.7 | 0.000427 |
| SMPDB | β-Ureidopropionase Deficiency | 57 | 6 | 10.7 | 0.000427 |
| SMPDB | Dihydropyrimidinase Deficiency | 57 | 6 | 10.7 | 0.000427 |
| Reactome | Transport of Nucleotide Sugars | 13 | 3 | 23.1 | 0.005356 |
| HumanCyc | Rapoport-Luebering Glycolytic Shunt | 7 | 2 | 50.0 | 0.009228 |
| Reactome | Pyrimidine Biosynthesis | 22 | 3 | 14.3 | 0.012456 |
| HumanCyc | Guanine & Guanosine Salvage | 7 | 2 | 28.6 | 0.016529 |
| HumanCyc | L-Dopachrome Biosynthesis | 8 | 2 | 28.6 | 0.016529 |
| Wikipathways | Dopamine Metabolism | 32 | 3 | 10.7 | 0.018653 |
|  | **Down Pathways** |  |  |  |  |
| KEGG | Gly, Ser, & Thr Metabolism | 50 | 6 | 15.4 | 0.015701 |
| SMPDB | Carnosinuria, Carnosinemia | 34 | 5 | 15.2 | 0.027247 |
| SMPDB | Ureidopropionase Deficiency | 34 | 5 | 15.2 | 0.027247 |
| SMPDB | GABA-Transaminase Deficiency | 34 | 5 | 15.2 | 0.027247 |
| SMPDB | β-Alanine Metabolism | 34 | 5 | 15.2 | 0.027247 |
| HumanCyc | Adenosine Nucleotides Degradation | 13 | 3 | 25.0 | 0.030001 |
| SMPDB | Plasmalogen Synthesis | 14 | 3 | 25.0 | 0.030001 |
