# Supplementary material for: Divergent organ-specific isogenic metastatic cell lines identified using multi-omics exhibit differential drug sensitivity
Source: PLoS One. 2020 Nov 16;15(11):e0242384. doi: 10.1371/journal.pone.0242384 (PMC7668614; doi:10.1371/journal.pone.0242384)
Supplement: S32 Table — (DOCX) [file pone.0242384.s043.docx]

| **S32 Table. Metabolomic-based Unique pathways for the metastatic Spine-435 cell line.** | | | | | |
| --- | --- | --- | --- | --- | --- |
| **Source** | **Up Pathways** | **# of Meta-**  **bolites in Set** | **# of Obs. Meta-bolites** | **Obs. Meta-**  **bolites (%)** | **q-value** |
| EHMN | Urea cycle & Metabolism of Arg, Pro, Glu, Asp, & Asn | 125 | 9 | 9.9 | 0.000275 |
| Reactome | SLC-Mediated Transmembrane Transport | 166 | 11 | 7.2 | 0.000325 |
| Reactome | Amino Acid & Oligopeptide SLC Transporters | 50 | 6 | 14.3 | 0.000845 |
| Reactome | Transport of Inorganic Cations/Anions & Amino Acids/Oligopeptides | 56 | 6 | 12.5 | 0.001222 |
| Reactome | Transport of Small Molecules | 226 | 11 | 5.7 | 0.001222 |
| INOH | Ala, Asp, & Asn Metabolism | 45 | 5 | 12.8 | 0.003454 |
| KEGG | Ala, Asp, & Glut Metabolism | 28 | 4 | 17.4 | 0.004162 |
| KEGG | Protein Digestion & Absorption | 47 | 5 | 11.1 | 0.004282 |
| Reactome | Transport of Bile Salts & Organic Acids, Metal Ions & Amine Compounds | 78 | 6 | 8.6 | 0.004282 |
| HumanCyc | γ-Glutamyl Cycle | 29 | 4 | 14.3 | 0.004282 |
|  | **Down Pathways** |  |  |  |  |
| Wikipathways | TCA Cycle and Deficiency of Pyruvate Dehydrogenase Complex (PDHc) | 19 | 5 | 38.5 | 0.000678 |
| Reactome | MicroRNA (miRNA) Biogenesis | 6 | 3 | 60.0 | 0.00298 |
| Reactome | Mitochondrial Translation Initiation | 8 | 3 | 60.0 | 0.00298 |
| PID | Netrin-mediated Signaling Events | 5 | 3 | 60.0 | 0.00298 |
| Reactome | Activation of GABA-B Receptors | 6 | 3 | 50.0 | 0.004756 |
| Reactome | GABA-B Receptor Activation | 6 | 3 | 50.0 | 0.004756 |
| SMPDB | Insulin Signaling | 6 | 3 | 50.0 | 0.004756 |
| PID | IL2 Signaling Events Mediated by PI3K | 6 | 3 | 50.0 | 0.004756 |
| BioCarta | Visual Signal Transduction | 6 | 3 | 50.0 | 0.004756 |
| HumanCyc | Terminal O-Glycans residues Modification | 9 | 3 | 50.0 | 0.004756 |
