# Supplementary material for: Divergent organ-specific isogenic metastatic cell lines identified using multi-omics exhibit differential drug sensitivity
Source: PLoS One. 2020 Nov 16;15(11):e0242384. doi: 10.1371/journal.pone.0242384 (PMC7668614; doi:10.1371/journal.pone.0242384)
Supplement: S33 Table — (DOCX) [file pone.0242384.s044.docx]

| **S33 Table. Common metabolomic and proteomic pathways for the metastatic Brain-435 cell line.** | | | | | | | | | |  |
| --- | --- | --- | --- | --- | --- | --- | --- | --- | --- | --- |
| **Source** | **Up Pathways** | **# of Metabo-**  **lites in**  **Set** | **# of**  **Obs.**  **Metabo-**  **lites** | **Obs.**  **Metabo-**  **lites**  **(%)** | **q-value** | **# of Proteins in Set** | **# of Obs. Proteins** | **Obs. Proteins (%)** | **q-value** | |
| Wikipathways | Amino Acid Metabolism | 108 | 3 | 3.3 | 0.043015 | 91 | 13 | 14.3 | 0.0030 | |
| KEGG | Val, Leu, & Ile Degradation | 42 | 2 | 7.7 | 0.043015 | 48 | 8 | 16.7 | 0.0072 | |
|  | **Down Pathways** |  |  |  |  |  |  |  |  | |
| Reactome | Cell Cycle | 33 | 8 | 26.7 | 0.000188 | 564 | 94 | 16.7 | 1.93E-13 | |
| Reactome | Cell Cycle, Mitotic | 30 | 8 | 27.6 | 0.000161 | 481 | 81 | 16.9 | 8.43E-12 | |
| Wikipathways | Pyrimidine Metabolism | 40 | 10 | 25.0 | 2.2E-05 | 84 | 24 | 28.6 | 4.25E-07 | |
| Reactome | Translation | 78 | 5 | 16.7 | 0.005589 | 310 | 50 | 16.3 | 1.57E-06 | |
| KEGG | Pyrimidine Metabolism | 66 | 13 | 24.5 | 2.5E-06 | 101 | 25 | 24.8 | 2.19E-06 | |
| Reactome | Metabolism of Nucleotides | 152 | 28 | 22.4 | 2.99E-12 | 105 | 17 | 16.2 | 0.007536 | |
| Reactome | Metabolism | 1384 | 71 | 8.2 | 1.43E-10 | 1972 | 163 | 8.3 | 0.017658 | |
| EHMN | Pyrimidine Metabolism | 77 | 19 | 26.8 | 1.75E-09 | 136 | 18 | 13.3 | 0.029783 | |
| Reactome | Post-translational Protein Modification | 176 | 18 | 19.8 | 2.94E-07 | 1383 | 118 | 8.6 | 0.025374 | |
| INOH | Pyrimidine Nucleotides Nucleosides Metabolism | 56 | 14 | 25.5 | 4.92E-07 | 51 | 10 | 19.6 | 0.017658 | |
| Reactome | S Phase | 17 | 8 | 47.1 | 3.24E-06 | 103 | 19 | 18.4 | 0.001254 | |
| Reactome | DNA Replication | 14 | 7 | 50.0 | 1.18E-05 | 8 | 5 | 62.5 | 0.001512 | |
| KEGG | Purine metabolism | 95 | 14 | 18.2 | 3.38E-05 | 174 | 22 | 12.7 | 0.023862 | |
| Reactome | Extension of Telomeres | 14 | 6 | 46.2 | 8.75E-05 | 30 | 9 | 30.0 | 0.002221 | |
| Reactome | Selenoamino Acid Metabolism | 61 | 9 | 20.5 | 0.000498 | 130 | 18 | 14.0 | 0.021678 | |
