# Supplementary material for: Divergent organ-specific isogenic metastatic cell lines identified using multi-omics exhibit differential drug sensitivity
Source: PLoS One. 2020 Nov 16;15(11):e0242384. doi: 10.1371/journal.pone.0242384 (PMC7668614; doi:10.1371/journal.pone.0242384)
Supplement: S34 Table — (DOCX) [file pone.0242384.s045.docx]

| **S34 Table. Common metabolomic and proteomic pathways for the metastatic Liver-435 cell line.** | | | | | | | | | |  |
| --- | --- | --- | --- | --- | --- | --- | --- | --- | --- | --- |
| **Source** | **Up Pathways** | **# of Metabo-**  **lites in**  **Set** | **# of**  **Obs.**  **Metabo-**  **lites** | **Obs.**  **Metabo-**  **lites**  **(%)** | **q-value** | **# of Proteins in Set** | **# of Obs. Proteins** | **Obs. Proteins (%)** | **q-value** | |
|  | No Pathways |  |  |  |  |  |  |  |  | |
|  | **Down Pathways** |  |  |  |  |  |  |  |  | |
| EHMN | Pyrimidine Metabolism | 77 | 24 | 33.8 | 4.03E-14 | 136 | 17 | 12.6 | 0.004035 | |
| Reactome | Nucleotide Salvage | 45 | 17 | 38.6 | 8.05E-11 | 23 | 6 | 26.1 | 0.007890 | |
| EHMN | Glycolysis & Gluconeogenesis | 52 | 16 | 41.0 | 1.12E-10 | 67 | 9 | 13.6 | 0.029166 | |
| Reactome | Interconversion of Nucleotide Di- & Triphosphates | 52 | 16 | 36.4 | 2.89E-10 | 34 | 7 | 20.6 | 0.011154 | |
| Reactome | S Phase | 17 | 10 | 58.8 | 6.18E-09 | 103 | 16 | 15.5 | 0.001168 | |
| HumanCyc | Superpathway of Purine Nucleotides Salvage | 30 | 12 | 41.4 | 1.39E-08 | 59 | 10 | 16.9 | 0.006104 | |
| Reactome | DNA Replication | 14 | 9 | 64.3 | 1.14E-08 | 80 | 13 | 16.2 | 0.001931 | |
| KEGG | Purine Metabolism | 95 | 18 | 23.4 | 2.39E-08 | 174 | 18 | 10.4 | 0.015470 | |
| KEGG | Pyrimidine Metabolism | 66 | 15 | 28.3 | 3.55E-08 | 101 | 20 | 19.8 | 1.06E-06 | |
| Reactome | Telomere C-strand (Lagging Strand) Synthesis | 14 | 8 | 61.5 | 1.51E-07 | 24 | 7 | 29.2 | 0.001902 | |
| Reactome | TCA Cycle & Respiratory Electron Transport | 56 | 13 | 27.7 | 4.64E-07 | 173 | 18 | 10.4 | 0.015470 | |
| Reactome | Cell Cycle | 33 | 10 | 33.3 | 2.51E-06 | 564 | 62 | 11.0 | 7.73E-07 | |
| Reactome | Cell Cycle, Mitotic | 30 | 10 | 34.5 | 1.81E-06 | 481 | 50 | 10.4 | 2.46E-05 | |
| Wikipathways | Pyrimidine Metabolism | 40 | 10 | 27.8 | 1.43E-05 | 84 | 20 | 23.8 | 1.12E-06 | |
| Reactome | Translation | 78 | 6 | 20.0 | 0.001207 | 310 | 37 | 12.1 | 2.46E-05 | |
