# Supplementary material for: Divergent organ-specific isogenic metastatic cell lines identified using multi-omics exhibit differential drug sensitivity
Source: PLoS One. 2020 Nov 16;15(11):e0242384. doi: 10.1371/journal.pone.0242384 (PMC7668614; doi:10.1371/journal.pone.0242384)
Supplement: S35 Table — (DOCX) [file pone.0242384.s046.docx]

| **S35 Table.** **Common metabolomic and proteomic pathways for the metastatic Lung-435 cell line.** | | | | | | | | | |  |
| --- | --- | --- | --- | --- | --- | --- | --- | --- | --- | --- |
| **Source** | **Up Pathways** | **# of Metabo-**  **lites in**  **Set** | **# of**  **Obs.**  **Metabo-**  **lites** | **Obs.**  **Metabo-**  **lites**  **(%)** | **q-value** | **# of Proteins in Set** | **# of Obs. Proteins** | **Obs. Proteins (%)** | **q-value** | |
| SMPDB | Pyrimidine Metabolism | 57 | 6 | 10.7 | 0.000427 | 23 | 5 | 21.7 | 0.035408 | |
| Reactome | Metabolism of Amino Acids & Derivatives | 285 | 8 | 3.5 | 0.015904 | 342 | 26 | 7.7 | 0.190700 | |
|  | **Down Pathways** |  |  |  |  |  |  |  |  | |
| Reactome | Metabolism of Carbohydrates | 137 | 24 | 24.7 | 2.46E-11 | 264 | 29 | 11.0 | 0.003890 | |
| SMPDB | Warburg Effect | 58 | 17 | 33.3 | 6.05E-10 | 45 | 9 | 20.0 | 0.006704 | |
| SMPDB | Pentose Phosphate Pathway | 29 | 12 | 46.2 | 5.32E-09 | 14 | 5 | 35.7 | 0.006704 | |
| EHMN | Glycolysis & Gluconeo-genesis | 52 | 12 | 30.8 | 9.06E-07 | 67 | 10 | 15.2 | 0.019833 | |
| SMPDB | Glycogenosis, Type IA, von Grieke Disease | 34 | 10 | 33.3 | 3.39E-06 | 22 | 5 | 22.7 | 0.030960 | |
| Reactome | Interconversion of Nucleotide Di- & Triphosphate | 52 | 7 | 15.9 | 0.007558 | 34 | 7 | 20.6 | 0.021339 | |
| SMPDB | Pyrimidine Metabolism | 57 | 12 | 21.4 | 1.73E-05 | 23 | 5 | 21.7 | 0.015304 | |
| HumanCyc | Superpathway of Conversion of Glucose to Acetyl CoA & Entry into the TCA Cycle | 36 | 9 | 30.0 | 2.05E-05 | 48 | 11 | 23.4 | 0.000819 | |
| KEGG | Pyrimidine Metabolism | 66 | 11 | 20.3 | 6.32E-05 | 101 | 15 | 1409 | 0.004964 | |
| Reactome | Glucose Metabolism | 44 | 9 | 24.3 | 0.000114 | 91 | 12 | 13.3 | 0.022924 | |
| Wikipathways | Metabolic Reprogramming in Colon Cancer | 35 | 8 | 26.7 | 0.000170 | 42 | 7 | 16.7 | 0.035408 | |
| Reactome | Post-translational Protein Modification | 176 | 12 | 13.2 | 0.001378 | 1383 | 123 | 8.9 | 4.85E-06 | |
| Wikipathways | Cori Cycle | 24 | 6 | 30.0 | 0.000767 | 16 | 6 | 37.5 | 0.002216 | |
| Reactome | Asn N-linked Glycosylation | 119 | 10 | 16.7 | 0.000776 | 286 | 26 | 9.1 | 0.036800 | |
| KEGG | Glucagon Signaling Pathway | 26 | 6 | 28.6 | 0.000952 | 103 | 13 | 12.6 | 0.025181 | |
