# Supplementary material for: Divergent organ-specific isogenic metastatic cell lines identified using multi-omics exhibit differential drug sensitivity
Source: PLoS One. 2020 Nov 16;15(11):e0242384. doi: 10.1371/journal.pone.0242384 (PMC7668614; doi:10.1371/journal.pone.0242384)
Supplement: S36 Table — (DOCX) [file pone.0242384.s047.docx]

| **S36 Table. Common metabolomic and proteomic pathways for the metastatic Spine-435 cell line.** | | | | | | | | | |  |
| --- | --- | --- | --- | --- | --- | --- | --- | --- | --- | --- |
| **Source** | **Up Pathways** | **# of Metabo-**  **lites in**  **Set** | **# of**  **Obs.**  **Metabo-**  **lites** | **Obs.**  **Metabo-**  **lites**  **(%)** | **q-value** | **# of Proteins in Set** | **# of Obs. Proteins** | **Obs. Proteins (%)** | **q-value** | |
| Reactome | Amino Acid & Oligopeptide SLC Transporters | 50 | 6 | 14.3 | 0.000845 | 52 | 5 | 9.6 | 0.0282 | |
| Reactome | Amino Acid Transport Across the Plasma Membrane | 45 | 5 | 12.8 | 0.003454 | 32 | 5 | 15.6 | 0.0095 | |
|  | **Down Pathways** |  |  |  |  |  |  |  |  | |
| Reactome | Metabolism of Carbohydrates | 137 | 32 | 33 | 1.17E-19 | 264 | 32 | 12.2 | 9.55E-07 | |
| SMPDB | Warburg Effect | 58 | 22 | 43.1 | 7.86E-16 | 45 | 9 | 20.0 | 0.000819 | |
| Reactome | Metabolism of Nucleotides | 152 | 29 | 23.2 | 1.87E-13 | 105 | 11 | 10.5 | 0.013628 | |
| SMPDB | Gluconeogenesis | 34 | 16 | 47.1 | 1.87E-13 | 22 | 6 | 27.3 | 0.002041 | |
| EHMN | Glycolysis & Gluconeogenesis | 52 | 17 | 43.6 | 1.63E-12 | 67 | 11 | 16.7 | 0.000709 | |
| HumanCyc | Superpathway of Conversion of Glucose to Acetyl CoA & Entry into the TCA | 36 | 13 | 43.3 | 1.96E-09 | 48 | 15 | 31.9 | 1.03E-07 | |
| Wikipathways | Metabolic Reprogramming in Colon Cancer | 35 | 13 | 43.3 | 1.96E-09 | 42 | 8 | 21.4 | 0.000517 | |
| SMPDB | Fanconi-Bickel Syndrome | 24 | 11 | 50 | 7.27E-09 | 15 | 5 | 33.3 | 0.002514 | |
| SMPDB | Oncogenic Action of Succinate | 41 | 13 | 36.1 | 1.89E-08 | 31 | 5 | 16.1 | 0.027449 | |
| Reactome | Glucose Metabolism | 44 | 13 | 35.1 | 2.45E-08 | 71 | 17 | 24.3 | 2.20E-07 | |
| Wikipathways | Cori Cycle | 24 | 7 | 35.0 | 7.96E-05 | 16 | 7 | 43.8 | 5.24E-05 | |
| INOH | Citrate Cycle | 35 | 13 | 41.9 | 2.95E-09 | 32 | 8 | 25.0 | 0.000467 | |
| INOH | Aminosugars Metabolism | 32 | 7 | 23.3 | 0.000910 | 20 | 5 | 25.0 | 0.007542 | |
| Reactome | Cell Cycle, Mitotic | 30 | 7 | 24.1 | 0.000740 | 481 | 31 | 6.5 | 0.013469 | |
| Reactome | Pyruvate Metabolism & TCA Cycle | 44 | 12 | 30.0 | 4.23E-07 | 54 | 7 | 13.0 | 0.021418 | |
