# Supplementary material for: Divergent organ-specific isogenic metastatic cell lines identified using multi-omics exhibit differential drug sensitivity
Source: PLoS One. 2020 Nov 16;15(11):e0242384. doi: 10.1371/journal.pone.0242384 (PMC7668614; doi:10.1371/journal.pone.0242384)
Supplement: S37 Table — (DOCX) [file pone.0242384.s048.docx]

| **S37 Table. Common metabolomic and transcriptomic pathways for the metastatic Brain-435 cell line.** | | | | | | | | | | |
| --- | --- | --- | --- | --- | --- | --- | --- | --- | --- | --- |
| **Source** | **Up Pathways** | **# of Metabo-**  **lites in**  **Set** | **# of**  **Obs.**  **Metabo-**  **lites** | **Obs.**  **Metabo-**  **lites**  **(%)** | **q-value** | **# of Proteins in Set** | **# of Obs. Proteins** | **Obs. Proteins (%)** | **q-value** |  |
| INOH | Arg & Pro Metabolism | 68 | 3 | 5.4 | 0.033343 | 55 | 8 | 14.5 | 0.009856 |  |
|  | **Down Pathways** |  |  |  |  |  |  |  |  |  |
| Reactome | S Phase | 17 | 8 | 47.1 | 3.24E-06 | 103 | 17 | 16.5 | 2.42E-07 |  |
| Reactome | DNA Replication | 14 | 7 | 50.0 | 1.18E-05 | 80 | 18 | 22.5 | 1.27E-09 |  |
| Wikipathways | Pyrimidine Metabolism | 40 | 10 | 27.8 | 2.20E-05 | 84 | 10 | 11.9 | 0.002685 |  |
| Reactome | Chromosome Maintenance | 15 | 6 | 46.2 | 8.75E-05 | 112 | 15 | 13.5 | 1.98E-05 |  |
| Reactome | Telomere C-strand (Lagging Strand) Synthesis | 14 | 6 | 46.2 | 8.75E-05 | 24 | 8 | 33.3 | 9.84E-06 |  |
| Reactome | Telomere Maintenance | 14 | 6 | 46.2 | 8.75E-05 | 84 | 10 | 12.0 | 0.002489 |  |
| Reactome | Extension of Telemeres | 14 | 6 | 46.2 | 8.75E-05 | 30 | 8 | 26.7 | 4.21E-05 |  |
| Reactome | Synthesis of DNA | 13 | 6 | 46.2 | 8.75E-05 | 75 | 17 | 22.7 | 3.37E-09 |  |
| Reactome | Cell Cycle, Mitotic | 30 | 8 | 27.6 | 0.000161 | 481 | 42 | 8.8 | 3.70E-09 |  |
| Reactome | Cell Cycle | 33 | 8 | 26.7 | 0.000188 | 564 | 46 | 8.2 | 3.70E-09 |  |
| Reactome | DNA Repair | 48 | 8 | 18.6 | 0.002007 | 320 | 20 | 6.3 | 0.009710 |  |
| Reactome | Base Excision Repair | 22 | 5 | 25.0 | 0.004286 | 37 | 5 | 13.5 | 0.042310 |  |
