# Supplementary material for: Divergent organ-specific isogenic metastatic cell lines identified using multi-omics exhibit differential drug sensitivity
Source: PLoS One. 2020 Nov 16;15(11):e0242384. doi: 10.1371/journal.pone.0242384 (PMC7668614; doi:10.1371/journal.pone.0242384)
Supplement: S38 Table — (DOCX) [file pone.0242384.s049.docx]

| **S38 Table. Common metabolomic and transcriptomic pathways for the metastatic Liver-435 cell line.** | | | | | | | | | |  |
| --- | --- | --- | --- | --- | --- | --- | --- | --- | --- | --- |
| **Source** | **Up Pathways** | **# of Metabo-**  **lites in**  **Set** | **# of**  **Obs.**  **Metabo-**  **lites** | **Obs.**  **Metabo-**  **lites**  **(%)** | **q-value** | **# of Proteins in Set** | **# of Obs. Proteins** | **Obs. Proteins (%)** | **q-value** | |
|  | No Pathways |  |  |  |  |  |  |  |  | |
|  | **Down Pathways** |  |  |  |  |  |  |  |  | |
| EHMN | Pyrimidine Metabolism | 77 | 24 | 33.8 | 4.03E-14 | 136 | 22 | 16.3 | 0.001165 | |
| Reactome | Interconversion of Nucleotide Di- & Triphosphates | 52 | 16 | 36.4 | 2.89E-10 | 34 | 7 | 20.6 | 0.499990 | |
| EHMN | Pentose Phosphate Pathway | 37 | 13 | 43.3 | 1.79E-09 | 29 | 7 | 24.1 | 0.022426 | |
| Reactome | S Phase | 17 | 10 | 58.5 | 6.81E-09 | 103 | 42 | 40.8 | 1.40E-20 | |
| Reactome | DNA Replication | 14 | 9 | 64.3 | 1.41E-09 | 80 | 36 | 45.0 | 1.64E-19 | |
| KEGG | Pyrimidine Metabolism | 66 | 15 | 28.3 | 3.55E-08 | 101 | 17 | 16.8 | 0.004088 | |
| Reactome | Telomere C-strand (Lagging Strand) Synthesis | 14 | 8 | 61.5 | 1.51E-07 | 24 | 16 | 66.7 | 3.00E-12 | |
| EHMN | Urea Cycle & Metabolism of Arg, Pro, Glu, Asp, & Asn | 125 | 18 | 19.8 | 2.89E-07 | 106 | 15 | 14.3 | 0.035273 | |
| EHMN | Purine Metabolism | 94 | 17 | 20.7 | 3.52E-07 | 223 | 26 | 11.7 | 0.032856 | |
| Reactome | Cell Cycle, Mitotic | 30 | 10 | 34.5 | 1.81E-06 | 481 | 138 | 28.8 | 2.09E-49 | |
| Reactome | Cell Cycle | 33 | 10 | 33.3 | 2.51E-06 | 564 | 159 | 28.2 | 3.16E-56 | |
| Reactome | DNA Repair | 48 | 11 | 25.6 | 1.05E-05 | 320 | 77 | 24.3 | 1.50E-21 | |
| Reactome | DNA Replication Initiation | 8 | 5 | 62.5 | 5.52E-05 | 8 | 6 | 75.0 | 4.02E-05 | |
| Reactome | Transcriptional Regulation by TP53 | 37 | 8 | 28.6 | 9.77E-05 | 374 | 70 | 18.9 | 7.60E-13 | |
| Reactome | Base Excision Repair | 22 | 13 | 35.1 | 0.000544 | 37 | 13 | 35.1 | 8.18E-06 | |
