# Supplementary material for: Divergent organ-specific isogenic metastatic cell lines identified using multi-omics exhibit differential drug sensitivity
Source: PLoS One. 2020 Nov 16;15(11):e0242384. doi: 10.1371/journal.pone.0242384 (PMC7668614; doi:10.1371/journal.pone.0242384)
Supplement: S39 Table — (DOCX) [file pone.0242384.s050.docx]

| **S39 Table. Common metabolomic and transcriptomic pathways for the metastatic Lung-435 cell line.** | | | | | | | | | |  |
| --- | --- | --- | --- | --- | --- | --- | --- | --- | --- | --- |
| **Source** | **Up Pathways** | **# of Metabo-**  **lites in**  **Set** | **# of**  **Obs.**  **Metabo-**  **lites** | **Obs.**  **Metabo-**  **lites**  **(%)** | **q-value** | **# of Proteins in Set** | **# of Obs. Proteins** | **Obs. Proteins (%)** | **q-value** | |
| Reactome | Metabolism of Amino Acids & Derivatives | 285 | 8 | 3.5 | 0.015904 | 285 | 78 | 23.0 | 0.008223 | |
|  | **Down Pathways** |  |  |  |  |  |  |  |  | |
| Reactome | Metabolism of Carbohydrates | 137 | 24 | 24.7 | 2.46E-11 | 264 | 62 | 23.6 | 0.014486 | |
| INOH | Glycolysis Gluconeogenesis | 34 | 10 | 32.3 | 4.53E-06 | 46 | 17 | 37.8 | 0.017541 | |
| Reactome | Gluconeogenesis | 26 | 6 | 30.0 | 0.000767 | 35 | 14 | 41.2 | 0.021710 | |
