# Supplementary material for: Divergent organ-specific isogenic metastatic cell lines identified using multi-omics exhibit differential drug sensitivity
Source: PLoS One. 2020 Nov 16;15(11):e0242384. doi: 10.1371/journal.pone.0242384 (PMC7668614; doi:10.1371/journal.pone.0242384)
Supplement: S40 Table — (DOCX) [file pone.0242384.s051.docx]

| **S40 Table. Common metabolomic and transcriptomic pathways for the metastatic Spine-435 cell line.** | | | | | | | | | |  |
| --- | --- | --- | --- | --- | --- | --- | --- | --- | --- | --- |
| **Source** | **Up Pathways** | **# of Metabo-**  **lites in**  **Set** | **# of**  **Obs.**  **Metabo-**  **lites** | **Obs.**  **Metabo-**  **lites**  **(%)** | **q-value** | **# of Proteins in Set** | **# of Obs. Proteins** | **Obs. Proteins (%)** | **q-value** | |
| INOH | Arg & Pro Metabolism | 68 | 5 | 8.9 | 0.001058 | 55 | 9 | 16.4 | 0.048257 | |
| Reactome | His, Lys, Phe, Tyr, Pro, & Trp Catabolism | 91 | 5 | 6.2 | 0.005166 | 49 | 10 | 20.8 | 0.008216 | |
|  | **Down Pathways** |  |  |  |  |  |  |  |  | |
| SMPDB | Gluconeogenesis | 34 | 16 | 53.3 | 1.87E-13 | 22 | 7 | 31.8 | 0.004380 | |
| SMPDB | Glucogenosis, Type IA, von Gierke Disease | 34 | 6 | 53.3 | 1.87E-13 | 22 | 7 | 31.8 | 0.004380 | |
| HumanCyc | Superpathway of Conversion of Glucose to Acetyl CoA & Entry into the TCA Cycle | 36 | 13 | 43.3 | 1.96E-09 | 48 | 11 | 23.4 | 0.002374 | |
| Wikipathways | Metabolite Reprogramming in Colon Cancer | 35 | 13 | 43.3 | 1.96E-09 | 42 | 9 | 21.4 | 0.011440 | |
| SMPDB | Glycolysis | 24 | 11 | 50.0 | 7.27E-09 | 15 | 6 | 40.0 | 0.003959 | |
| SMPDB | Fanconi-Bickel Syndrome | 24 | 11 | 50.0 | 7.27E-07 | 15 | 6 | 40.0 | 0.003959 | |
| Reactome | Glucose Metabolsim | 44 | 13 | 35.1 | 2.45E-08 | 91 | 15 | 16.7 | 0.004380 | |
| HumanCyc | Gluconeogenesis | 26 | 10 | 38.5 | 3.84E-08 | 26 | 8 | 32.0 | 0.002374 | |
| Reactome | Gluconeogenesis | 34 | 11 | 32.4 | 1.25E-07 | 35 | 10 | 29.4 | 0.000929 | |
| Reactome | Glycolysis | 32 | 9 | 34.6 | 5.62E-06 | 71 | 13 | 18.6 | 0.004380 | |
| Reactome | Cell Cycle | 33 | 10 | 33.3 | 2.51E-06 | 564 | 159 | 28.2 | 3.16E-56 | |
| Reactome | S Phase | 17 | 7 | 41.2 | 2.49E-05 | 103 | 17 | 16.5 | 0.002776 | |
| KEGG | Glycolysis/Gluconeogenesis | 31 | 7 | 36.8 | 5.47E-05 | 68 | 13 | 19.1 | 0.003805 | |
| Wkipathways | Cori Cycle | 24 | 7 | 35.0 | 5.47E-05 | 16 | 6 | 37.5 | 0.004632 | |
| Reactome | DNA Replication | 14 | 6 | 42.9 | 9.28E-05 | 80 | 15 | 18.8 | 0.001931 | |
