# Supplementary material for: Divergent organ-specific isogenic metastatic cell lines identified using multi-omics exhibit differential drug sensitivity
Source: PLoS One. 2020 Nov 16;15(11):e0242384. doi: 10.1371/journal.pone.0242384 (PMC7668614; doi:10.1371/journal.pone.0242384)
Supplement: S41 Table — (DOCX) [file pone.0242384.s052.docx]

| **S41 Table. Proteomic-based pathways found to be up & down for the metastatic Brain-435 cell line.** | | | | | |  |
| --- | --- | --- | --- | --- | --- | --- |
| **Source** | **Pathways** | **# of Proteins in Set** | **# of Obs. Up/DN Proteins** | **Obs. Up/DN**  **Proteins (%)** | **Up/DN**  **q-values** | |
| NetPath | EGFR1 | 457 | 37/60 | 8.1/13.2 | 0.0007/3.2E-06 | |
| Reactome | Vesicle-mediated Transport | 620 | 37/58 | 6.0/9.4 | 0.014/0.04 | |
| Reactome | Neutrophil Degranulation | 490 | 34/51 | 7.0/10.5 | 0.004/0.01 | |
| Reactome | Apoptosis Execution Phase | 52 | 8/10 | 17.3/19.2 | 0.004/0.02 | |
| Wikipathways | Gene & Protein Expression by JAK-STAT Signaling after IL-12 Stiumulation | 36 | 5/8 | 13.9/22.2 | 0.07/0.02 | |
