# Supplementary material for: Divergent organ-specific isogenic metastatic cell lines identified using multi-omics exhibit differential drug sensitivity
Source: PLoS One. 2020 Nov 16;15(11):e0242384. doi: 10.1371/journal.pone.0242384 (PMC7668614; doi:10.1371/journal.pone.0242384)
Supplement: S42 Table — (DOCX) [file pone.0242384.s053.docx]

| **S42 Table. Proteomic-based pathways found to be up & down for the metastatic Liver-435 cell line.** | | | | | | |
| --- | --- | --- | --- | --- | --- | --- |
| **Source** | **Pathways** | **# of Proteins in Set** | **# of Obs. Up/DN Proteins** | **Obs. Up/DN**  **Proteins (%)** | **Up/DN**  **q-values** |  |
| NetPath | EGFR1 | 457 | 17/46 | 3.7/10.1 | 0.005/9.4E-05 |  |
| Reactome | Innate Immune System | 1077 | 27/69 | 2.5/6.5 | 0.013/0.033 |  |
| Reactome | Vesicle-mediated Transport | 620 | 24/51 | 3.9/8.7 | 0.00035/0.002 |  |
| Reactome | Neutrophil Degranulation | 490 | 20/43 | 4.1/8.9 | 0.0008/0.0015 |  |
| Reactome | Apoptosis | 118 | 7/17 | 5.9/14.4 | 0.014/0.0012 |  |
