# Supplementary material for: Divergent organ-specific isogenic metastatic cell lines identified using multi-omics exhibit differential drug sensitivity
Source: PLoS One. 2020 Nov 16;15(11):e0242384. doi: 10.1371/journal.pone.0242384 (PMC7668614; doi:10.1371/journal.pone.0242384)
Supplement: S43 Table — (DOCX) [file pone.0242384.s054.docx]

| **S43 Table. Proteomic-based pathways found to be up & down for the metastatic Lung-435 cell line.** | | | | | |  |
| --- | --- | --- | --- | --- | --- | --- |
| **Source** | **Pathways** | **# of Proteins in Set** | **# of Obs. Up/DN Proteins** | **Obs. Up/DN**  **Proteins (%)** | **Up/DN**  **q-values** | |
| NetPath | EGFR1 | 457 | 31/72 | 3.7/15.8 | 0.03/5.3E-13 | |
| KEGG | Endocytosis | 244 | 18/25 | 7.4/10.2 | 0.065/0.015 | |
| SMPDB | Warburg Effect | 45 | 9/9 | 20.0/20.0 | 0.003/0.007 | |
| EHMN | Glycolysis/ Glyconeogenesis | 67 | 9/10 | 13.6/15.2 | 0.02/0.02 | |
| Wikipathways | AGE-RAGE | 66 | 8/11 | 12.1/16.7 | 0.046/0.008 | |
