# Supplementary material for: Divergent organ-specific isogenic metastatic cell lines identified using multi-omics exhibit differential drug sensitivity
Source: PLoS One. 2020 Nov 16;15(11):e0242384. doi: 10.1371/journal.pone.0242384 (PMC7668614; doi:10.1371/journal.pone.0242384)
Supplement: S44 Table — (DOCX) [file pone.0242384.s055.docx]

| **S44 Table. Proteomic-based pathways found to be up & down for the metastatic Spine-435 cell line.** | | | | | |  |
| --- | --- | --- | --- | --- | --- | --- |
| **Source** | **Pathways** | **# of Proteins in Set** | **# of Obs. Up/DN Proteins** | **Obs. Up/DN**  **Proteins (%)** | **Up/DN**  **q-values** | |
| NetPath | EGFR1 | 457 | 16/34 | 3.5/7.5 | 0.05/0.0015 | |
| Reactome | Cycle Cycle, Mitotic | 481 | 16/31 | 3.3/6.5 | 0.066/0.014 | |
| Wikipathways | TGF-β Signaling | 132 | 7/12 | 5.3/9.1 | 0.066/0.02 | |
| Reactome | G2/M Transition | 137 | 7/15 | 5.1/10.9 | 0.074/0.0025 | |
| Reactome | Mitotic G2-G2/M Phase | 139 | 7/15 | 5.0/10.8 | 0.077/0.0028 | |
