# Supplementary material for: Divergent organ-specific isogenic metastatic cell lines identified using multi-omics exhibit differential drug sensitivity
Source: PLoS One. 2020 Nov 16;15(11):e0242384. doi: 10.1371/journal.pone.0242384 (PMC7668614; doi:10.1371/journal.pone.0242384)
Supplement: S45 Table — (DOCX) [file pone.0242384.s056.docx]

| **S45 Table.** **Proteomic-based pathways found to be up & down for the metastatic Lung-231 cell line.** | | | | | |  |
| --- | --- | --- | --- | --- | --- | --- |
| **Source** | **Pathways** | **# of Proteins in Set** | **# of Obs. Up/DN Proteins** | **Obs. Up/DN**  **Proteins (%)** | **Up/DN**  **q-values** | |
| Wikipathways | miRNA-targeted Genes in Lymphocytes - TarBase | 489 | 60/66 | 12.3/13.5 | 0.0025/7.2E-07 | |
| Wikipathways | miRNA-targeted Genes in Muscle – TarBase | 400 | 48/62 | 12.0/15.5 | 0.012/1.6E-08 | |
| PID | PDGFR-β Signaling Pathway | 127 | 18/20 | 14.2/15.7 | 0.05/0.0033 | |
