# Supplementary material for: Divergent organ-specific isogenic metastatic cell lines identified using multi-omics exhibit differential drug sensitivity
Source: PLoS One. 2020 Nov 16;15(11):e0242384. doi: 10.1371/journal.pone.0242384 (PMC7668614; doi:10.1371/journal.pone.0242384)
Supplement: S46 Table — (DOCX) [file pone.0242384.s057.docx]

| **S46 Table.** Proteomic-based pathways found to be up & down for the metastatic Lymph Node-231 cell line. | | | | | |  |
| --- | --- | --- | --- | --- | --- | --- |
| **Source** | **Pathways** | **# of Proteins in Set** | **# of Obs. Up/DN Proteins** | **Obs. Up/DN**  **Proteins (%)** | **Up/DN**  **q-values** | |
| NetPath | EGFR1 | 457 | 45/102 | 9.9/22.4 | 0.024/9.3E-16 | |
| Reactome | Post-translational Protein Modifications | 1383 | 111/170 | 8.1/12.3 | 0.05/0.0002 | |
| Wikipathways | miRNA-targeted Genes in Lymphocytes – TarBase | 489 | 57/86 | 11.7/17.6 | 0.0002/1.3E-07 | |
| Wikipathways | miRNA-targeted Genes in Muscle - TarBase | 400 | 39/67 | 9.8/20.1 | 0.05/4.2E-08 | |
| Wikipathways | miRNA-targeted Genes in Epithelium- TarBase | 333 | 39/75 | 11.7/18.8 | 0.003/9.2E-8 | |
| Reactome | Apoptotic Execution Phase | 52 | 13/11 | 25.0/21.2 | 0.0004/0.04 | |
